# Supplementary material for: Mild Copper-Catalyzed, l-Proline-Promoted Cross-Coupling of Methyl 3-Amino-1-benzothiophene-2-carboxylate
Source: Molecules. 2021 Nov 11;26(22):6822. doi: 10.3390/molecules26226822 (PMC8621900; doi:10.3390/molecules26226822)
Supplement: Supplementary file 1 [file molecules-26-06822-s001.zip › molecules-1424220-supplementary.pdf]

Supplementary Materials

Mild Copper-Catalyzed, *L*-Proline-Promoted Cross Coupling of Methyl 3-Amino-1-benzothiophene-2-carboxylate

Vilija Kederienė [1,*], Indrė Jaglinskaitė [1], Paulina Voznikaitė [1], Jolanta Rousseau [2], Patrick Rollin [3], Algirdas Šačkus [1] and Arnaud Tatibouët [3,*]

[1] Department of Organic Chemistry, Kaunas University of Technology, Radvilėnų pl. 19, LT-50254 Kaunas, Lithuania;

[2] Univ. Artois, CNRS, Centrale Lille, Univ. Lille, UMR 8181 – UCCS – Unité de Catalyse et Chimie du Solide, F-62300 Lens, France;

[3] Université d'Orléans et CNRS, ICOA, UMR 7311, BP 6759, F-45067 Orléans France.

***** Corresponding authors

E-mail address: vilija.kederiene@ktu.lt (V. Kederienė); arnaud.tatibouet@univ-orleans.fr (A. Tatibouët)

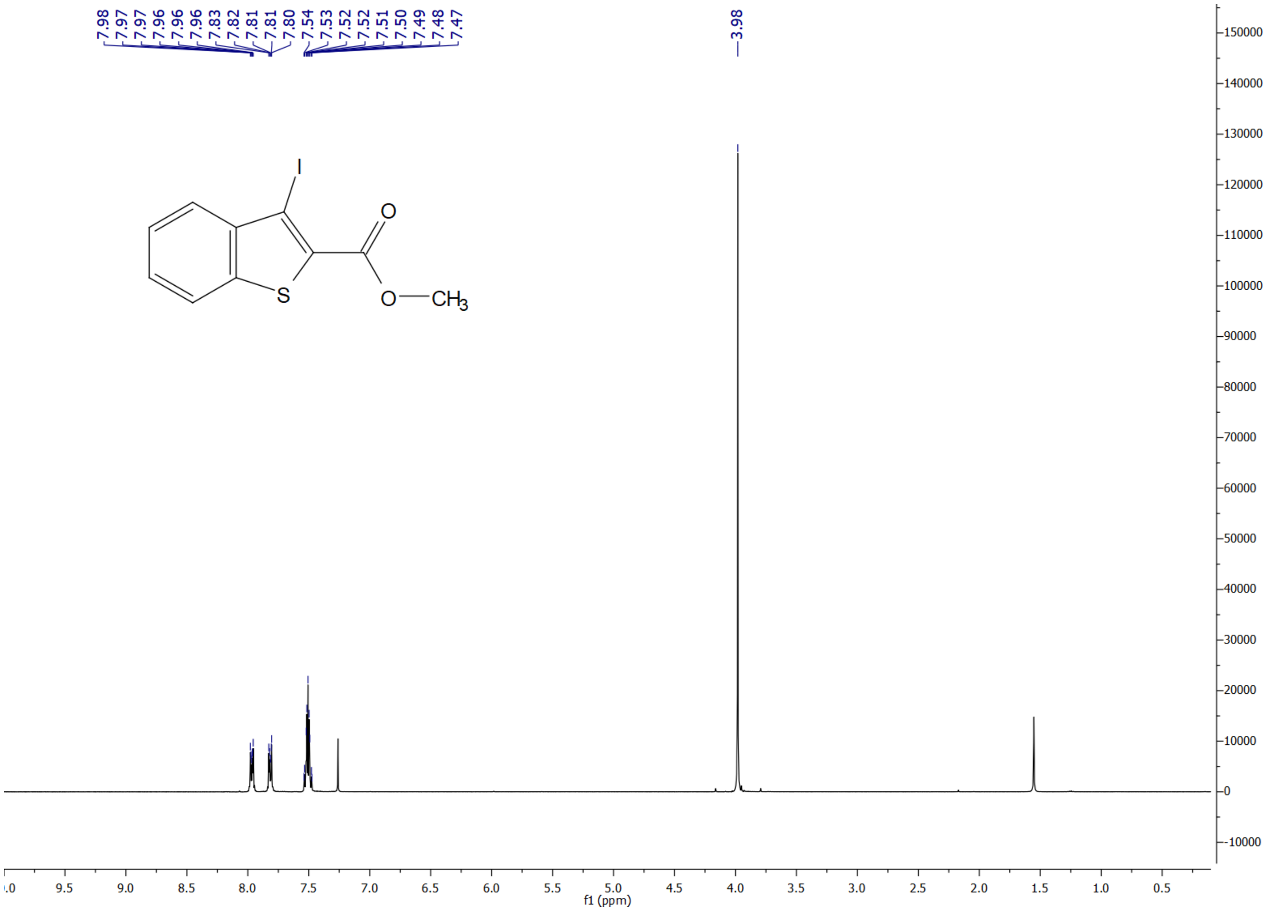

Figure S1. Methyl-3-iodo-1-benzothiophene-2-carboxylate (2). ^{1}H NMR spectrum (400 MHz, $CDCl_3$).

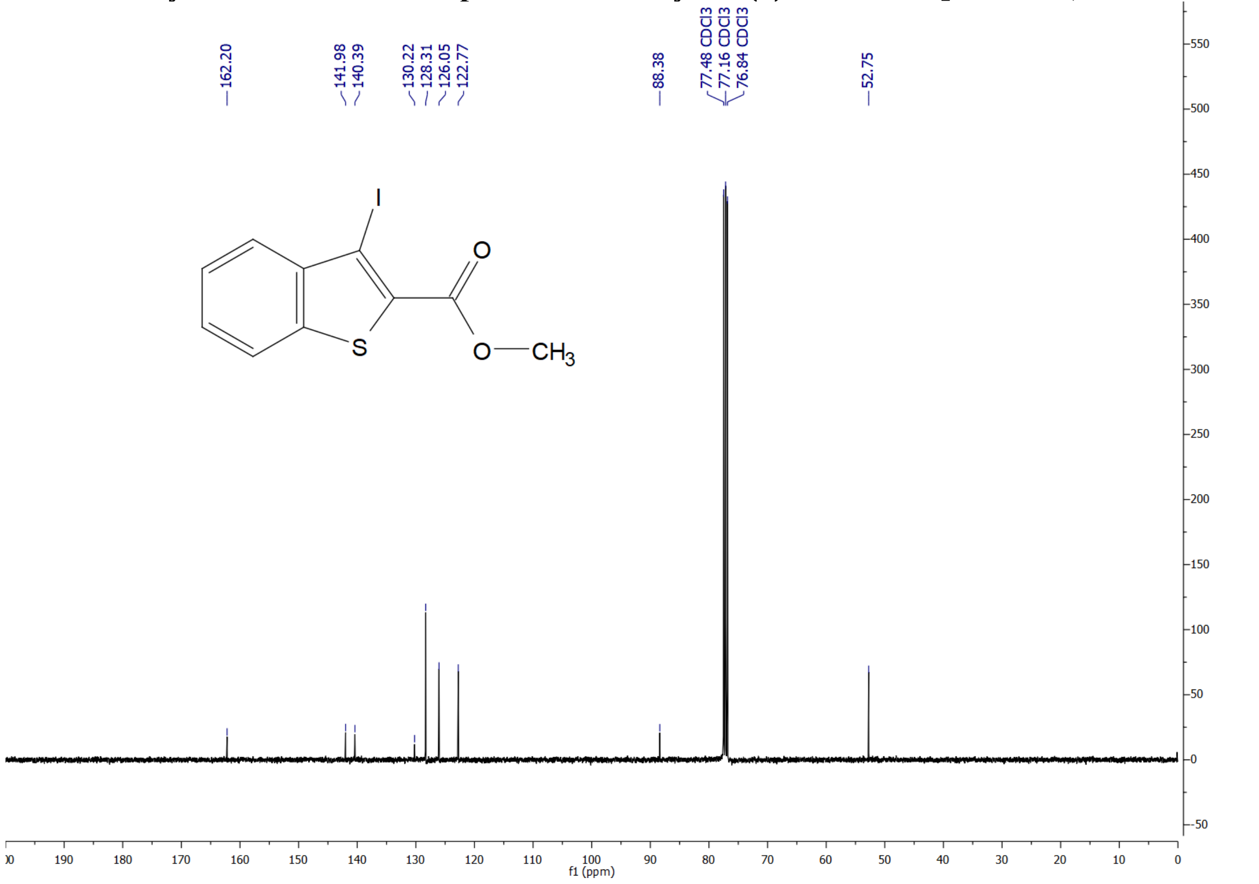

Figure S2. Methyl-3-iodo-1-benzothiophene-2-carboxylate (2). ^{13}C NMR spectrum (100 MHz, $CDCl_3$).

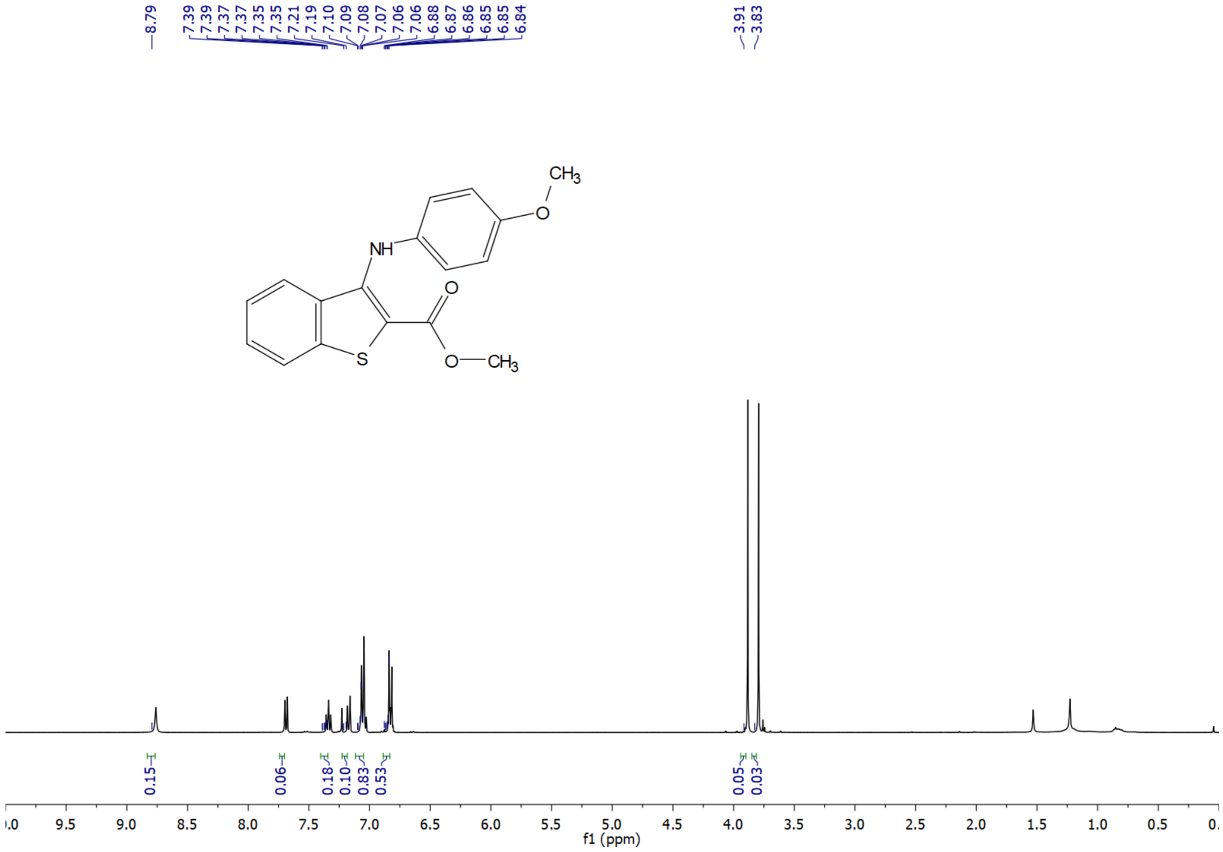

Figure S3. Methyl 3-[(4-methoxyphenyl)amino]-1-benzothiophene-2-carboxylate (3a). ^{1}H NMR spectrum (400 MHz, $CDCl_3$).

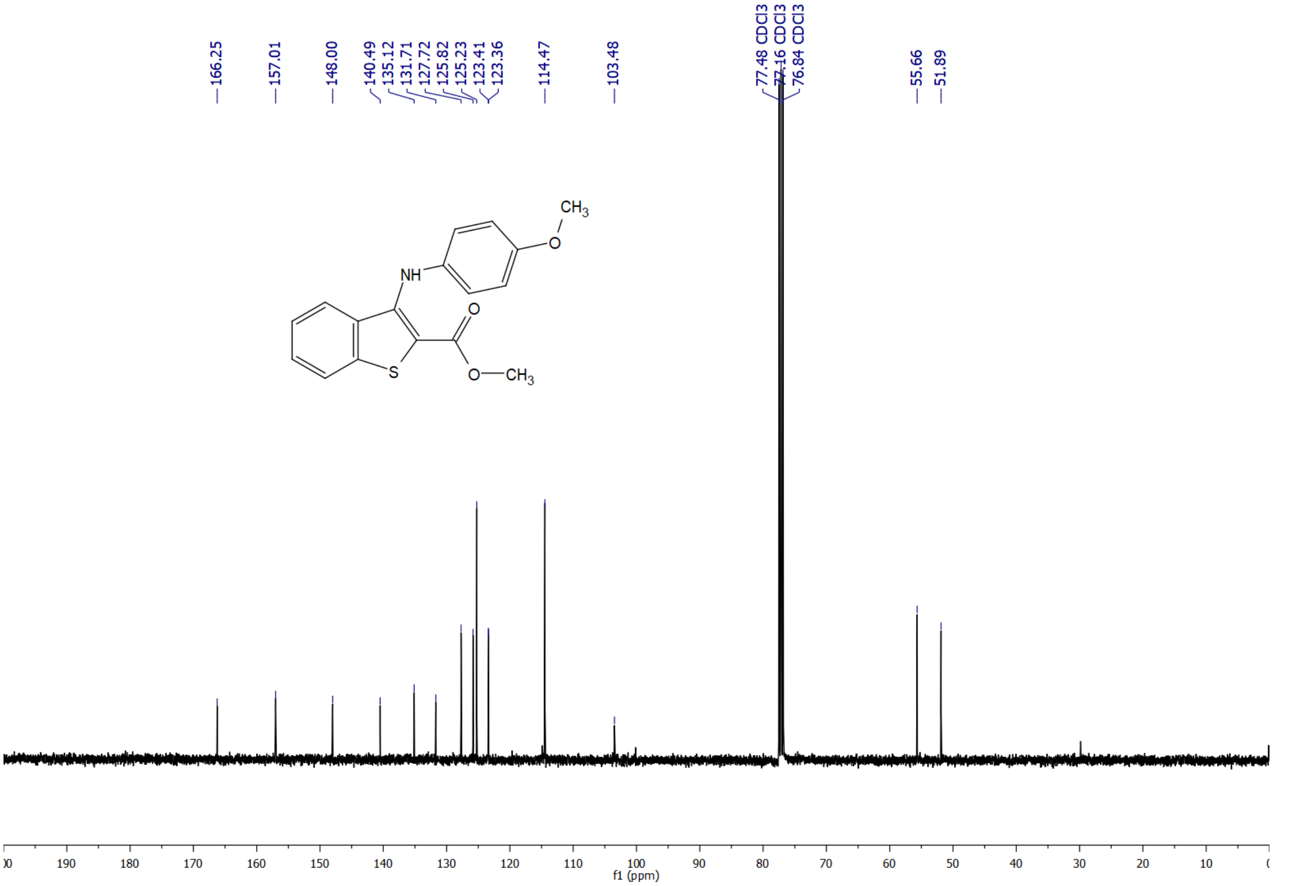

Figure S4. Methyl 3-[(4-methoxyphenyl)amino]-1-benzothiophene-2-carboxylate (3a). ^{13}C NMR spectrum (100 MHz, $CDCl_3$).

Acquisition Parameter

Source Type	ESI	Ion Polarity	Positive	Set Nebulizer	0.6 Bar
Focus	Active	Set Capillary	4500 V	Set Dry Heater	200 °C
Scan Begin	50 m/z	Set End Plate Offset	-500 V	Set Dry Gas	7.0 l/min
Scan End	1850 m/z	Set Collision Cell RF	2500.0 Vpp	Set Divert Valve	Waste

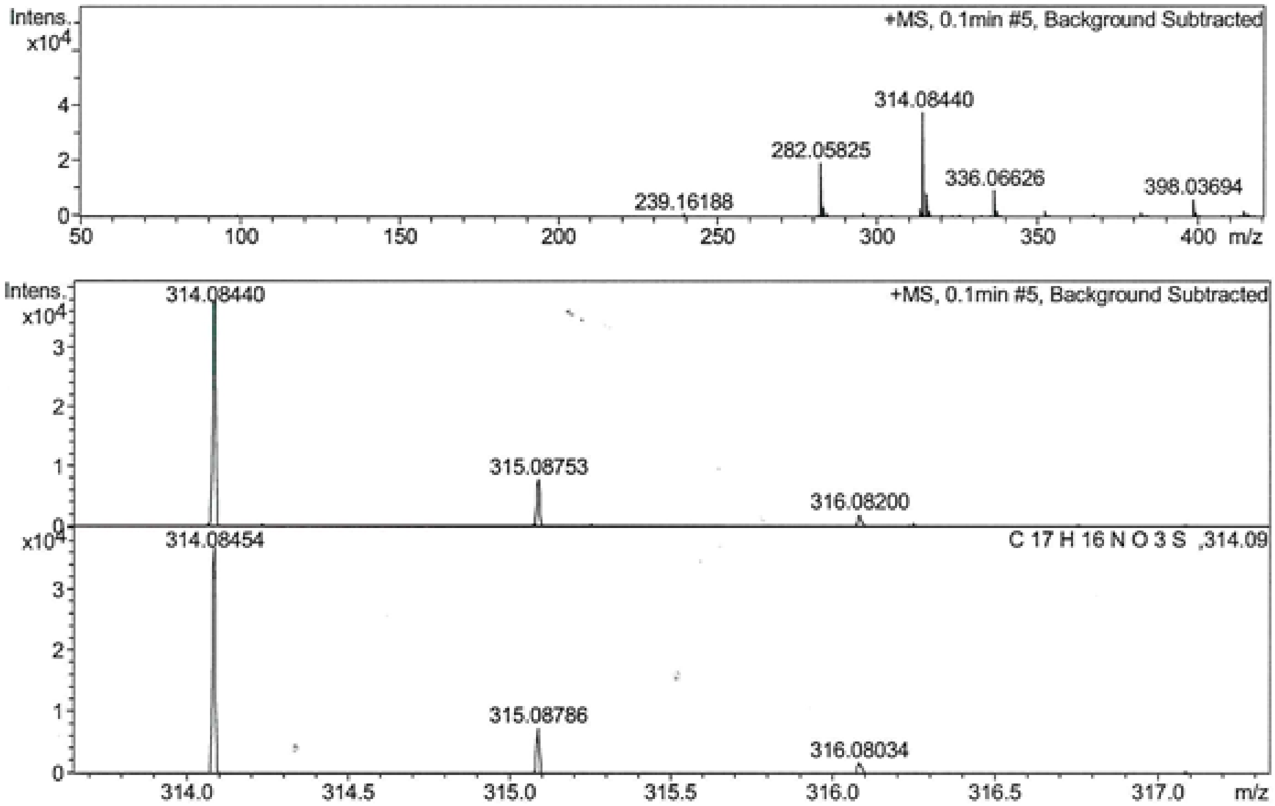

Meas. m/z	#	Formula	m/z	err [ppm]	mSigma	rdb	e⁻ Conf	N-Rule
282.05825	1	C 16 H 12 N O 2 S	282.05833	0.27	33.6	11.5	even	ok
314.08440	1	C 17 H 16 N O 3 S	314.08454	0.46	11.6	10.5	even	ok
336.06626	1	C 17 H 15 N Na O 3 S	336.06649	0.68	35.6	10.5	even	ok

Figure S5. Methyl 3-[(4-methoxyphenyl)amino]-1-benzothiophene-2-carboxylate (3a). HRMS (ESI).

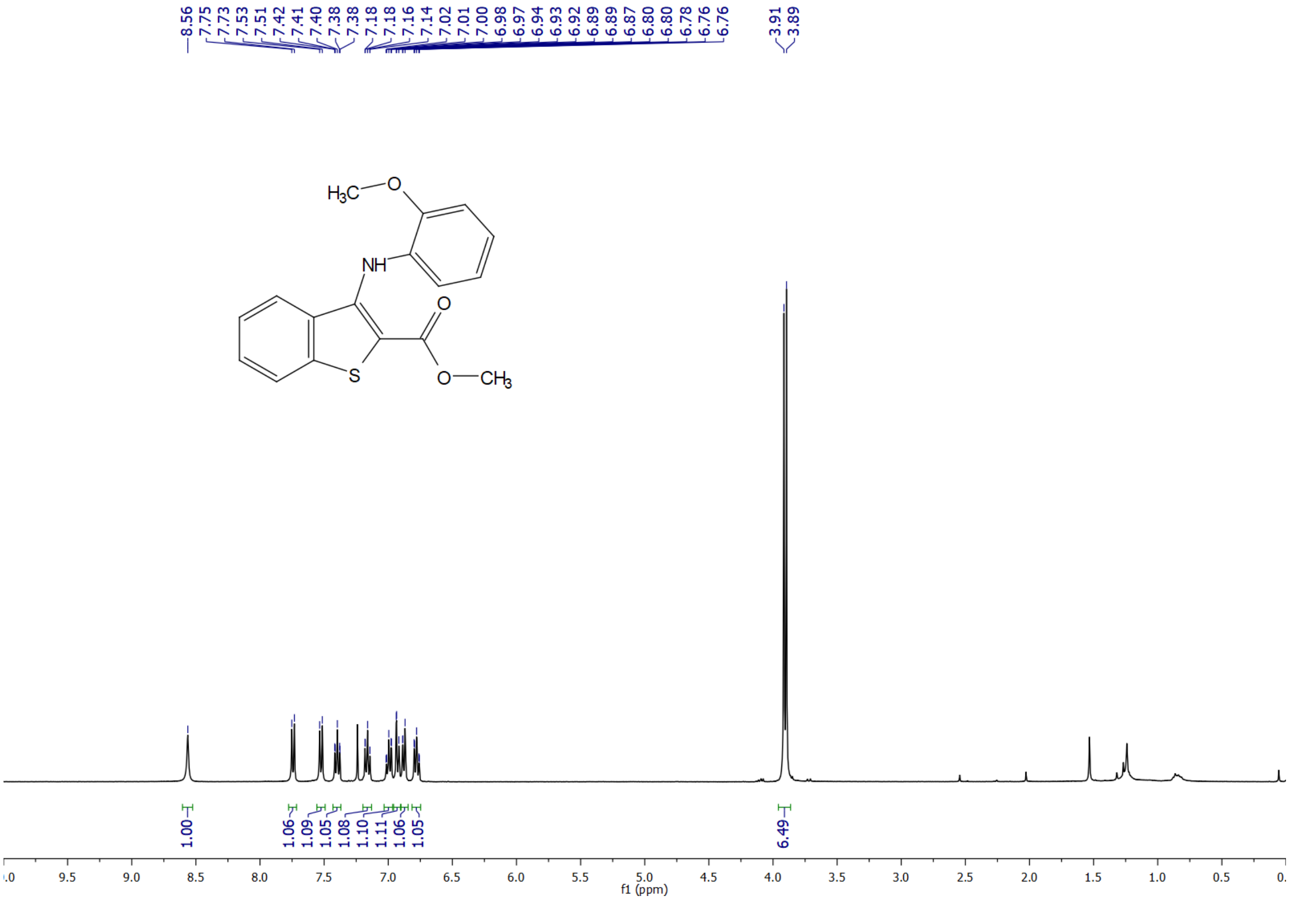

Figure S6. Methyl 3-[(2-methoxyphenyl)amino]-1-benzothiophene-2-carboxylate (3b). ^{1}H NMR spectrum (400 MHz, $CDCl_3$).

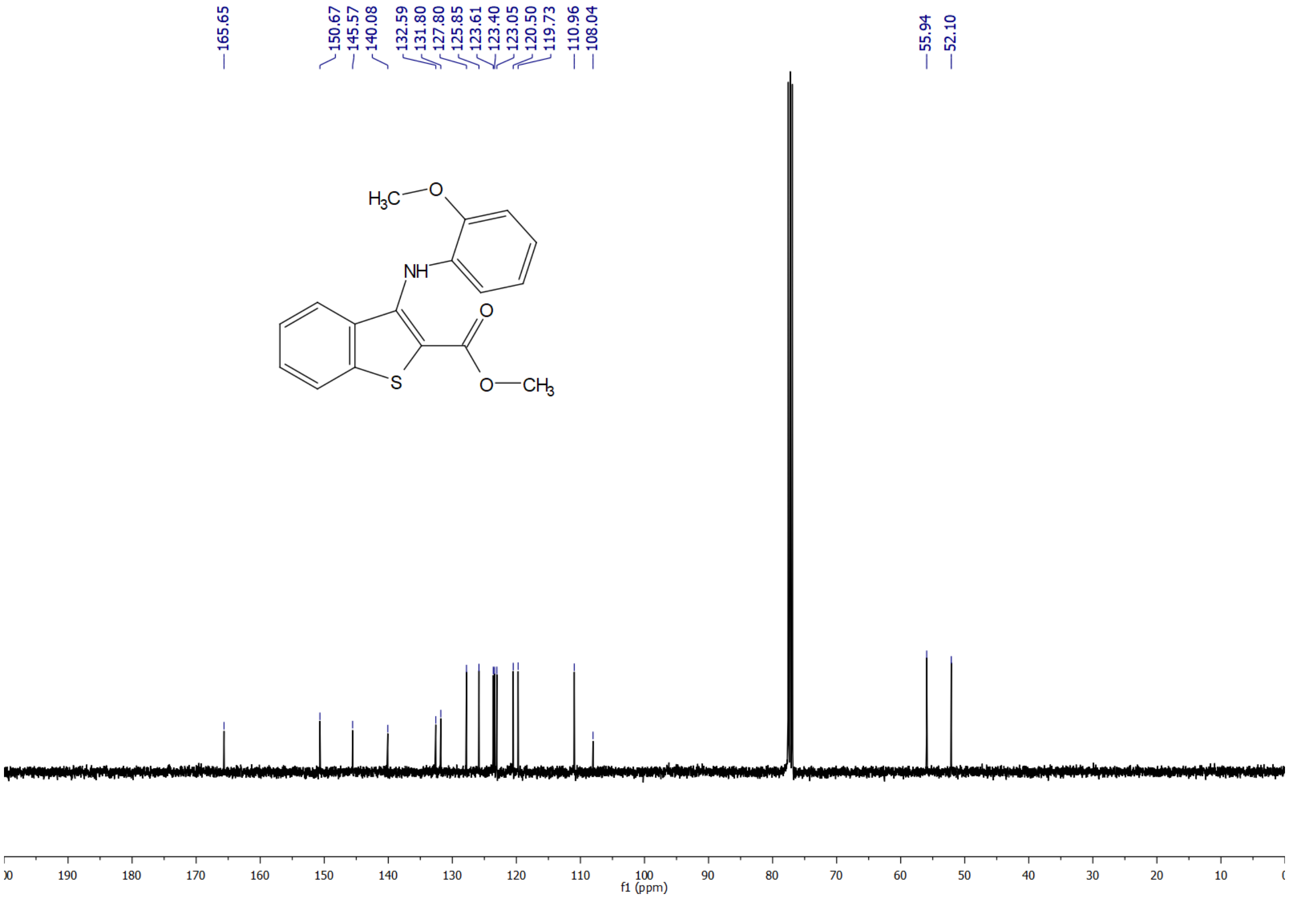

Figure S7. Methyl 3-[(2-methoxyphenyl)amino]-1-benzothiophene-2-carboxylate (3b). ^{13}C NMR spectrum (100 MHz, $CDCl_3$).

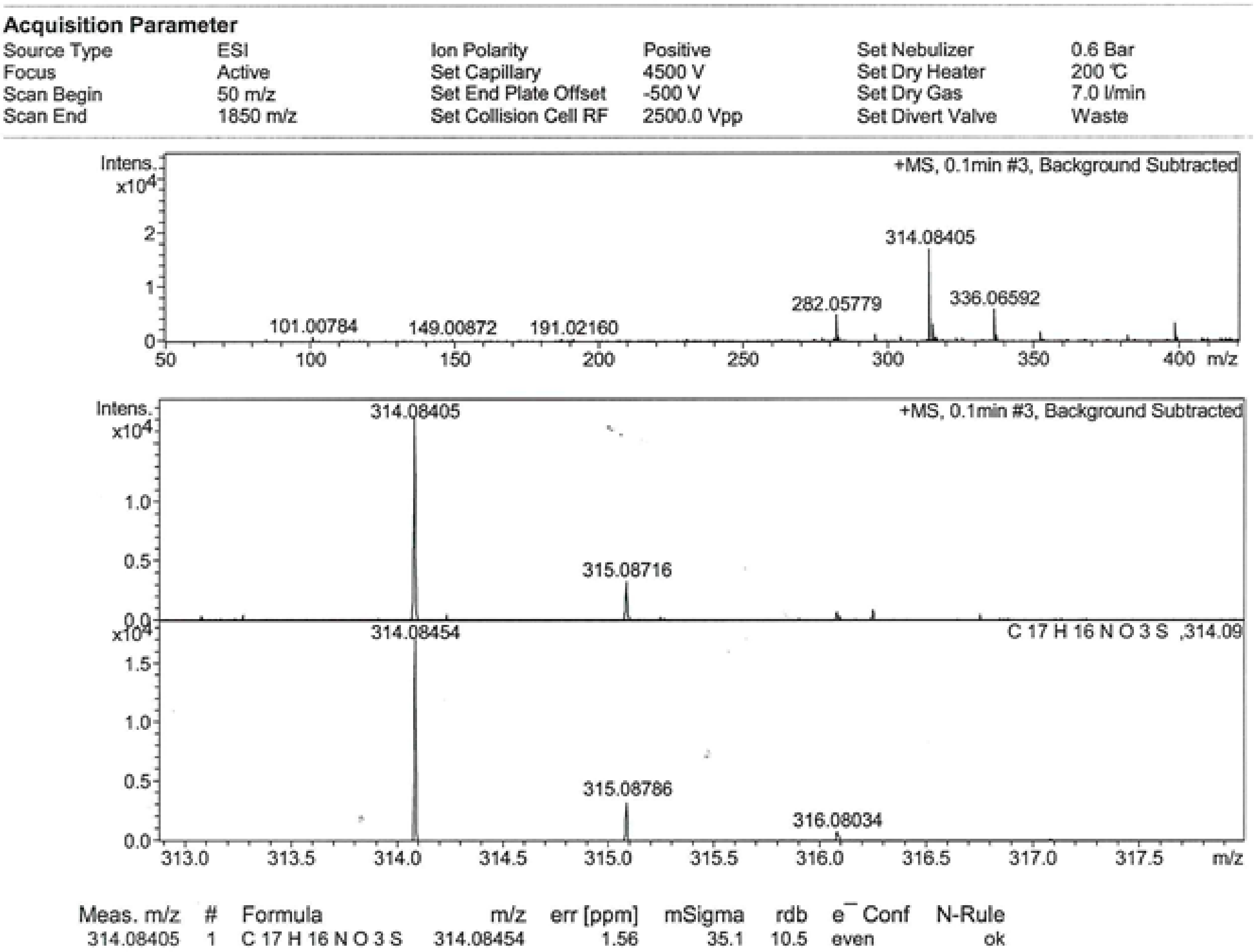

Figure S8. Methyl 3-[(2-methoxyphenyl)amino]-1-benzothiophene-2-carboxylate (3b). HRMS (ESI).

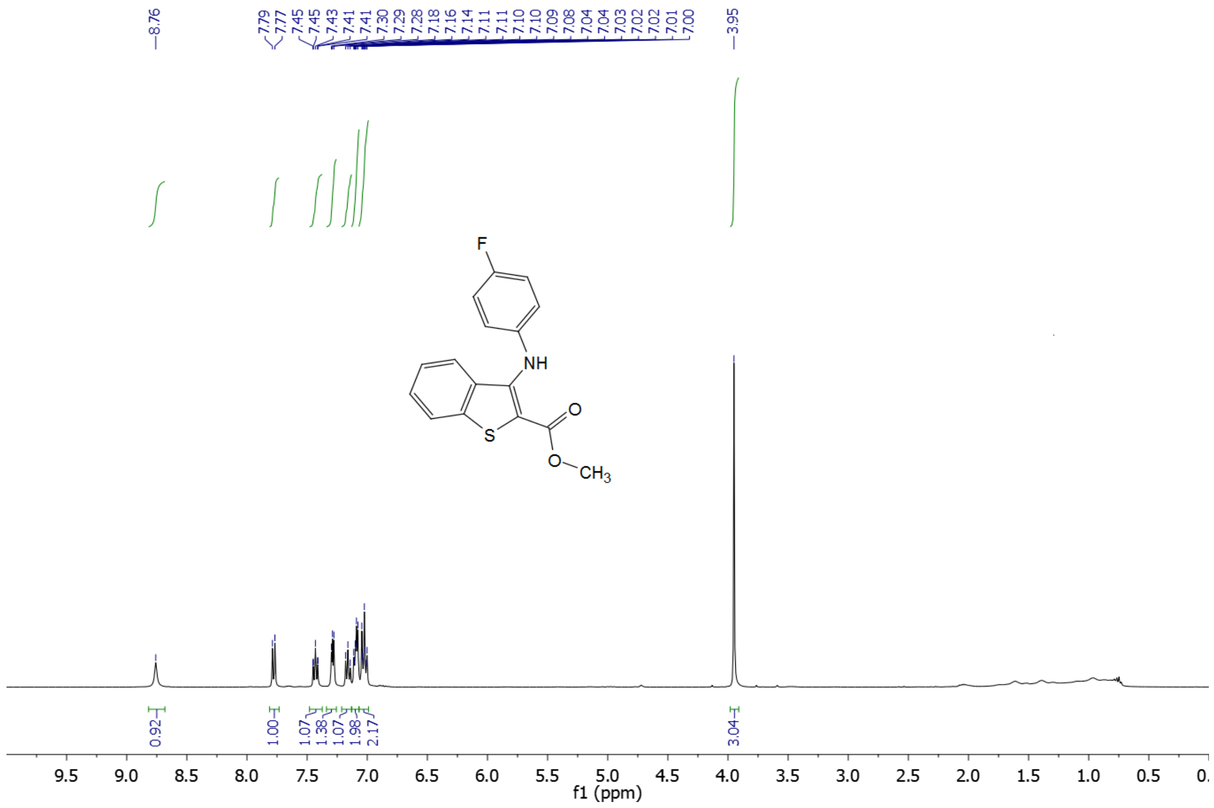

Figure S9. Methyl 3-[(4-fluorophenyl)amino]-1-benzothiophene-2-carboxylate (3c). ^{1}H NMR spectrum (400 MHz, $CDCl_3$).

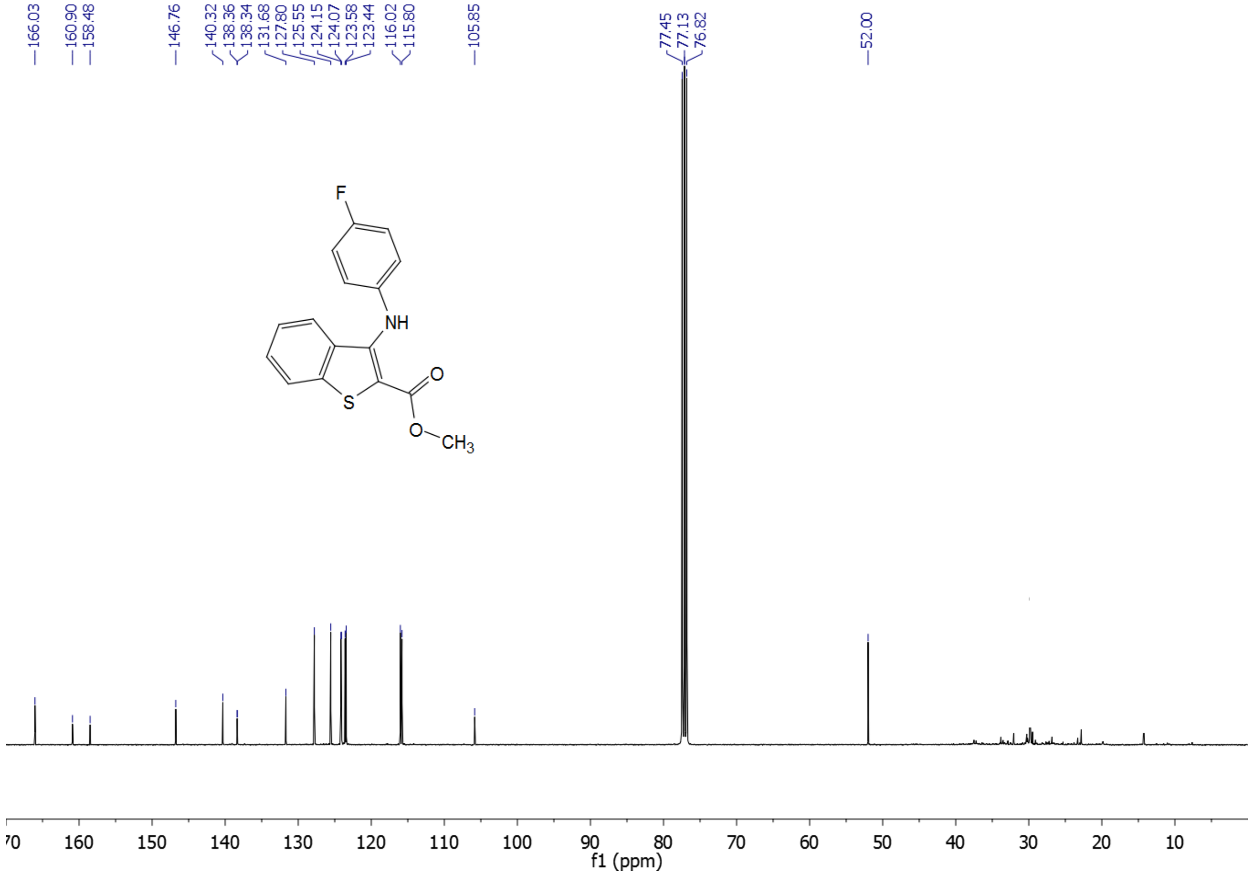

Figure S10. Methyl 3-[(4-fluorophenyl)amino]-1-benzothiophene-2-carboxylate (3c). ^{13}C NMR spectrum (100 MHz, $CDCl_3$).

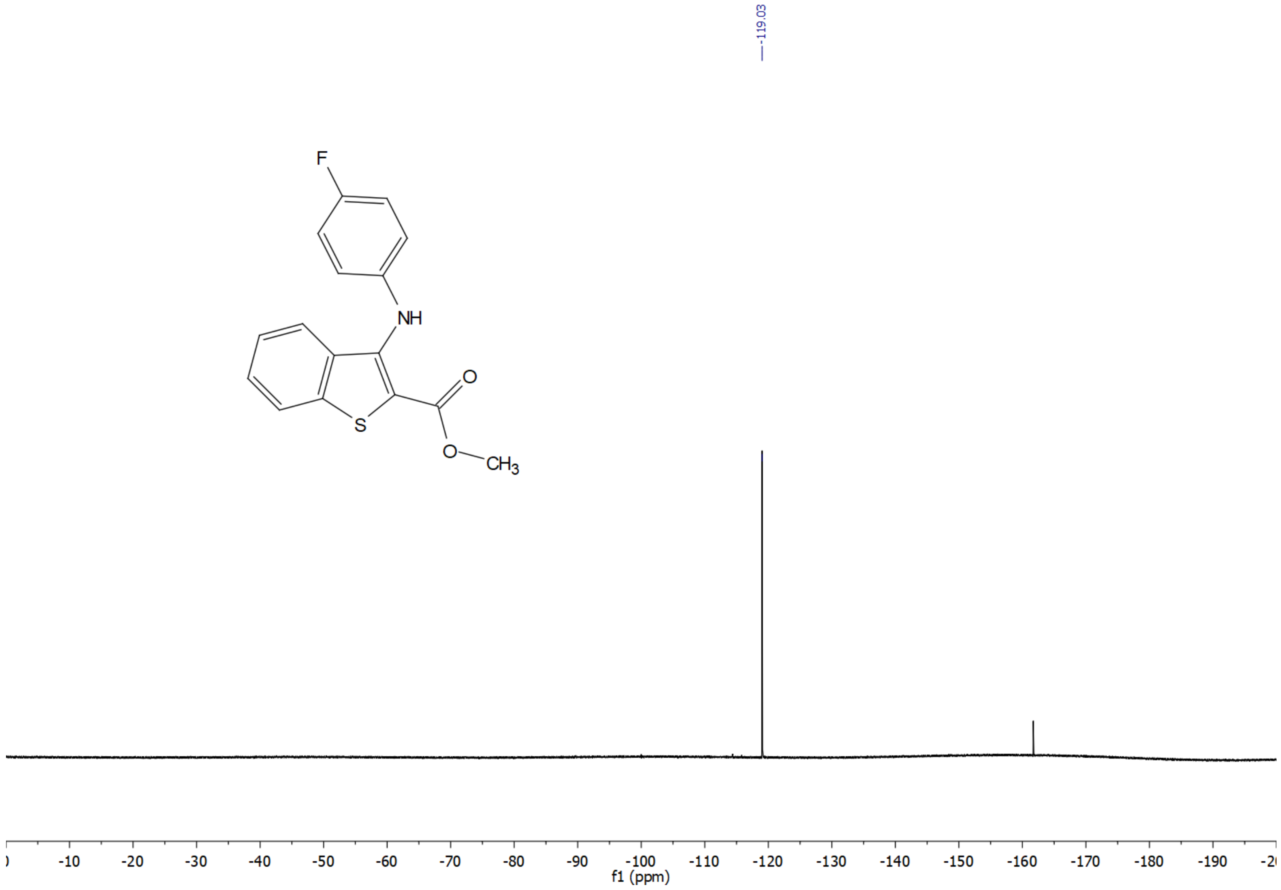

Figure S11. Methyl 3-[(4-fluorophenyl)amino]-1-benzothiophene-2-carboxylate (3c). ¹⁹F NMR spectrum (100 MHz, $CDCl_3$).

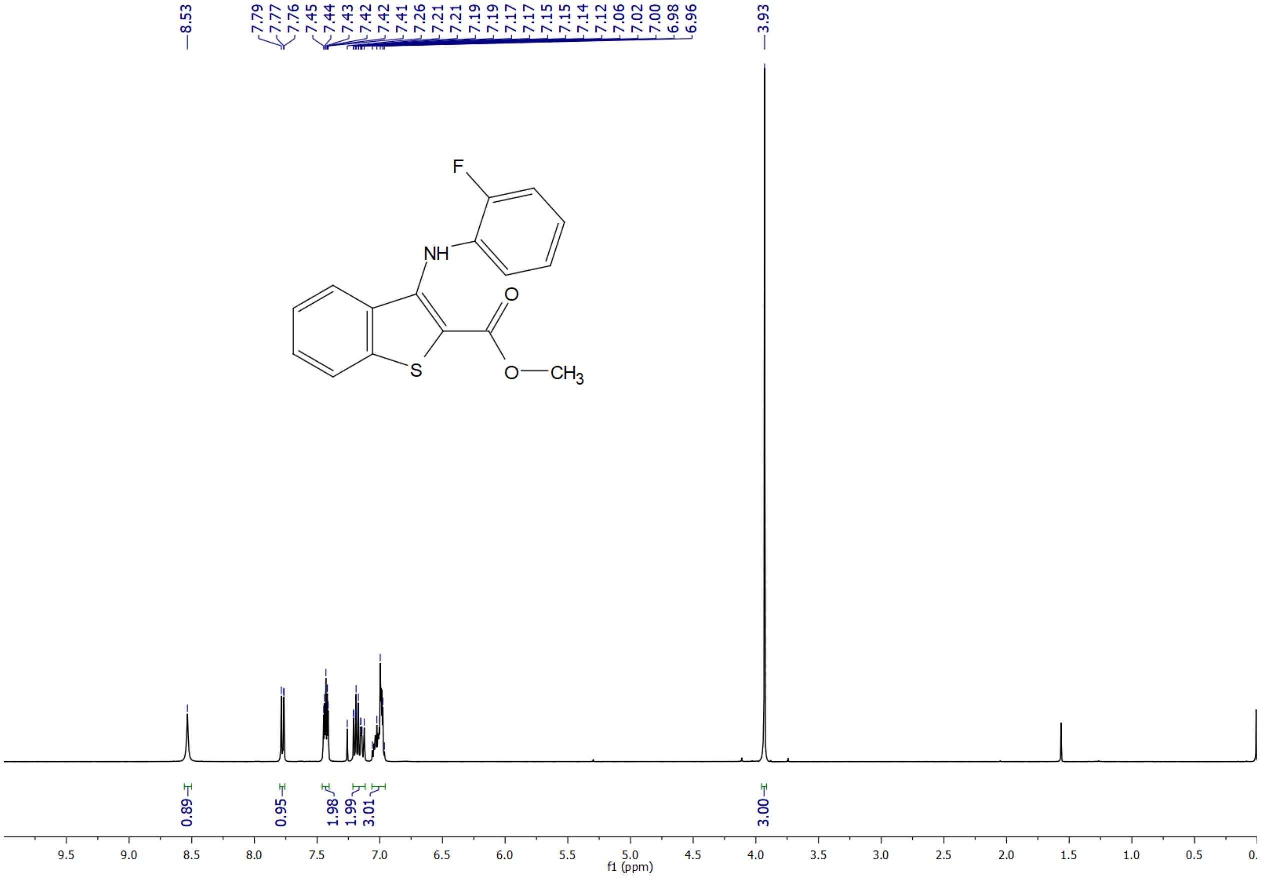

Figure S12. Methyl 3-[(2-fluorophenyl)amino]-1-benzothiophene-2-carboxylate (3d). ¹H NMR spectrum (400 MHz, $CDCl_3$).

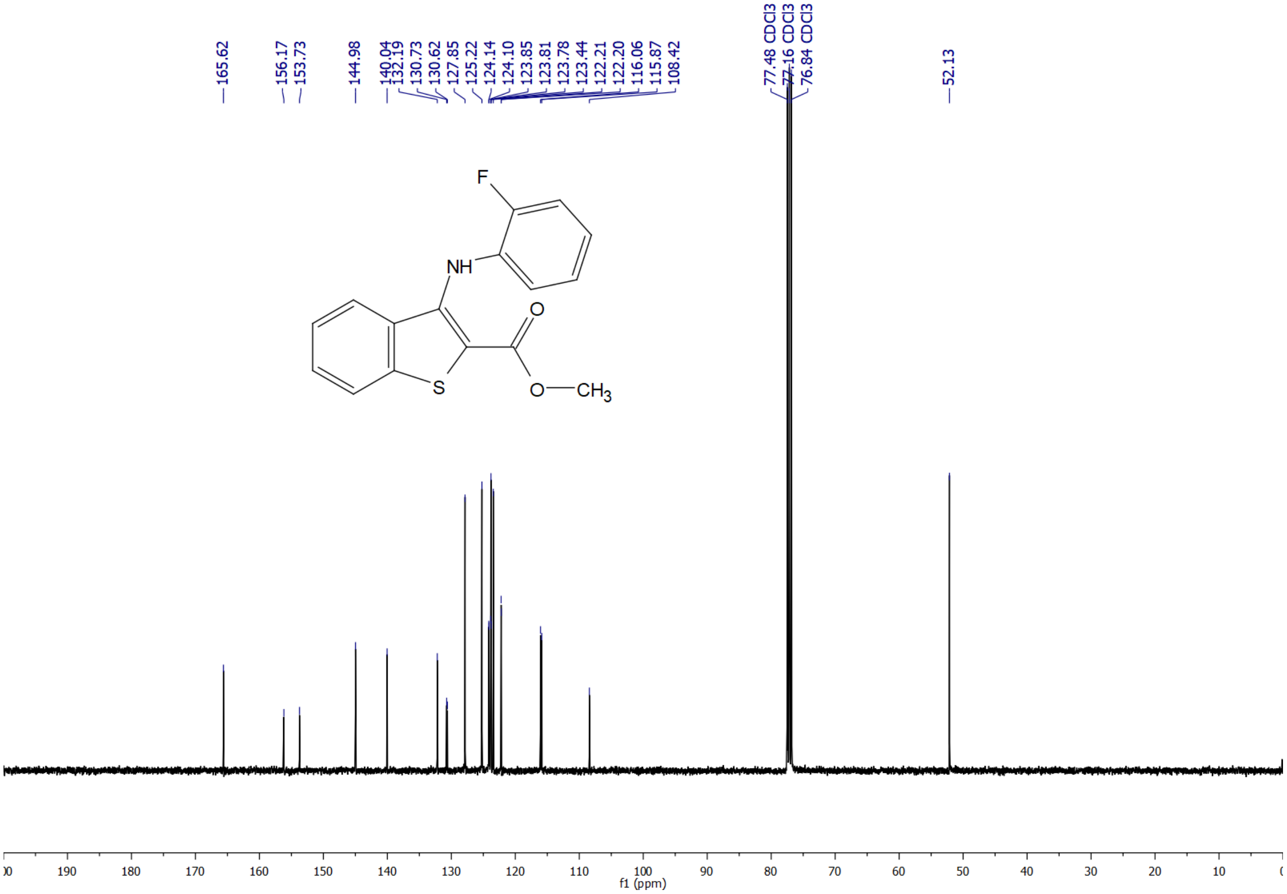

Figure S13. Methyl 3-[(2-fluorophenyl)amino]-1-benzothiophene-2-carboxylate (3d). ^{13}C NMR spectrum (100 MHz, $CDCl_3$).

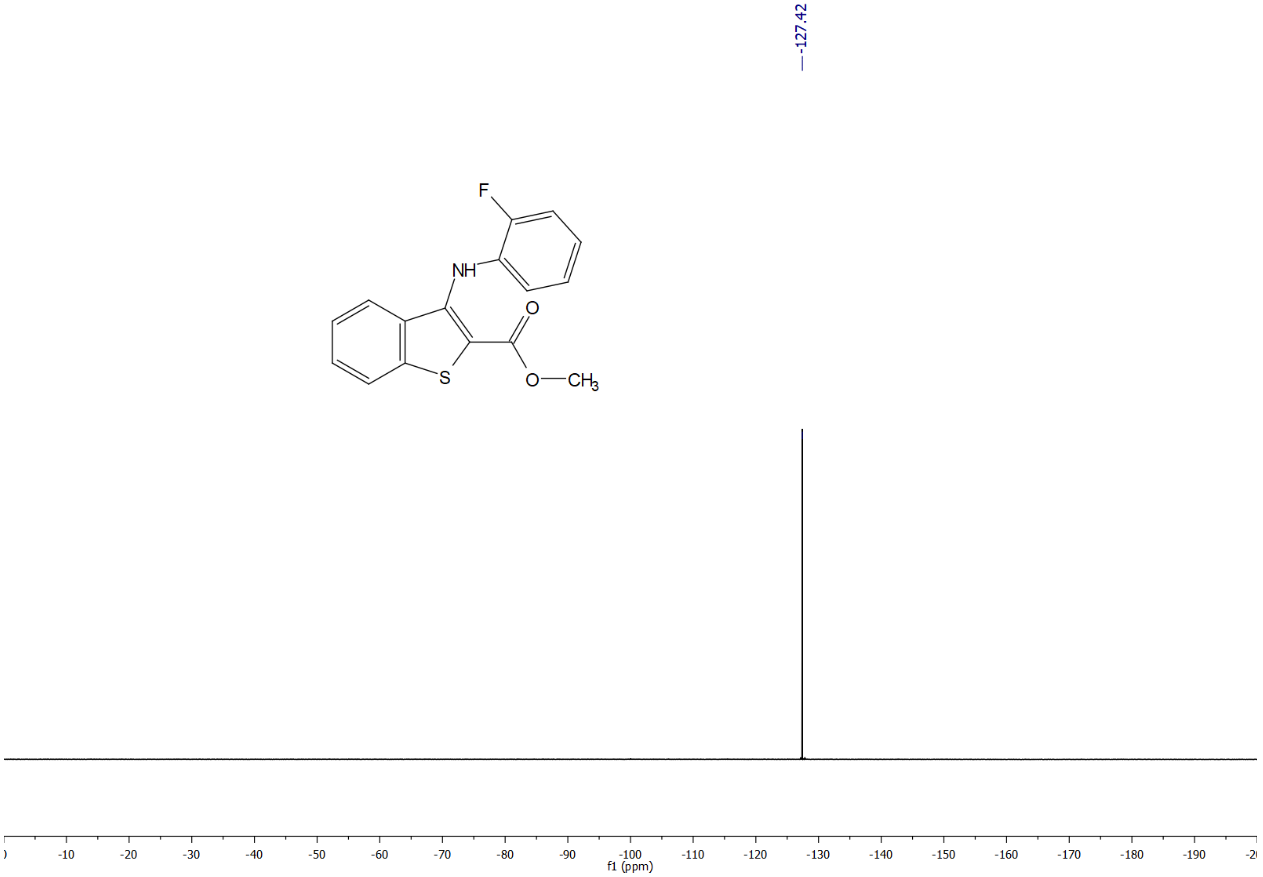

Figure S14. Methyl 3-[(2-fluorophenyl)amino]-1-benzothiophene-2-carboxylate (3d). ^{19}F NMR spectrum (400 MHz, $CDCl_3$).

Acquisition Parameter

Source Type	ESI	Ion Polarity	Positive	Set Nebulizer	0.6 Bar
Focus	Active	Set Capillary	4500 V	Set Dry Heater	200 ℃
Scan Begin	50 m/z	Set End Plate Offset	-500 V	Set Dry Gas	7.0 l/min
Scan End	1850 m/z	Set Collision Cell RF	2500.0 Vpp	Set Divert Valve	Waste

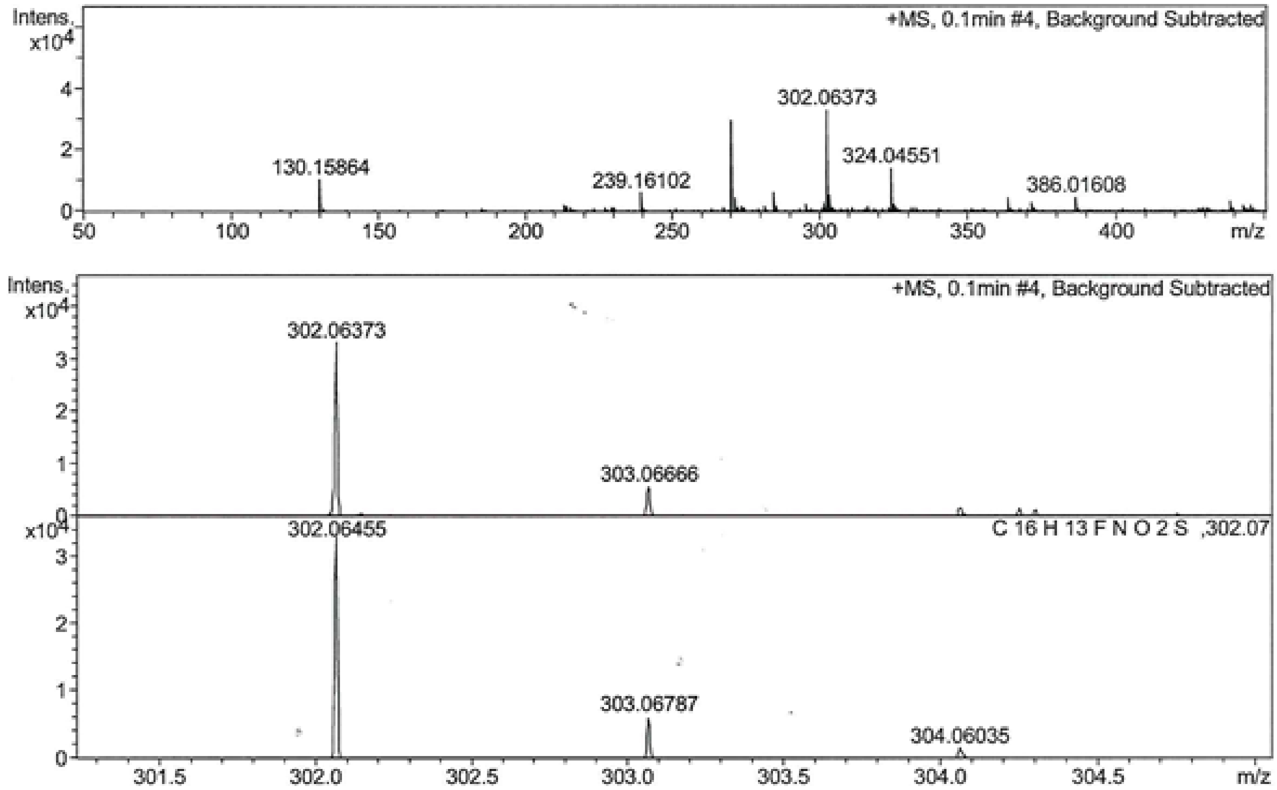

Meas. m/z	#	Formula	m/z	err [ppm]	mSigma	rdb	e¯ Conf	N-Rule
302.06373	1	C 16 H 13 F N O 2 S	302.06455	2.73	33.8	10.5	even	ok

Figure S15. Methyl 3-[(2-fluorophenyl)amino]-1-benzothiophene-2-carboxylate (3d). HRMS (ESI).

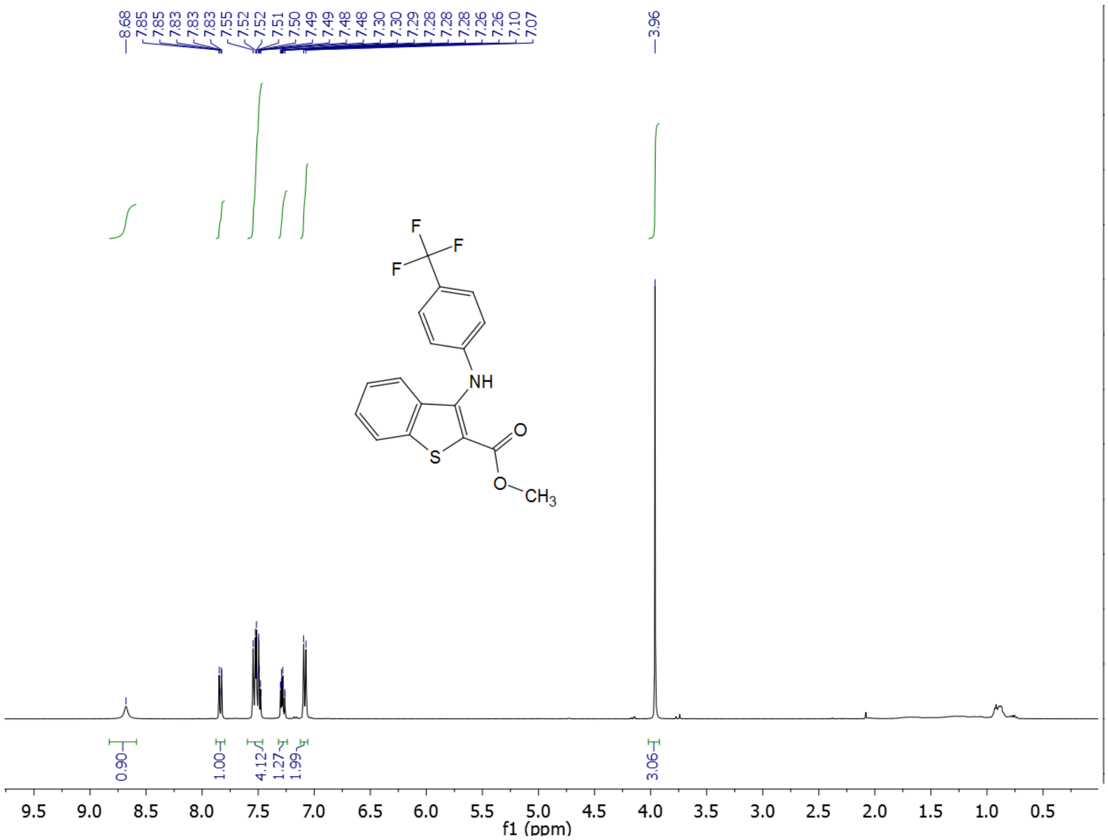

Figure S16. Methyl 3-{[4-(trifluoromethyl)phenyl]amino}-1-benzothiophene-2-carboxylate (3e). ^{1}H NMR spectrum (400 MHz, $CDCl_3$).

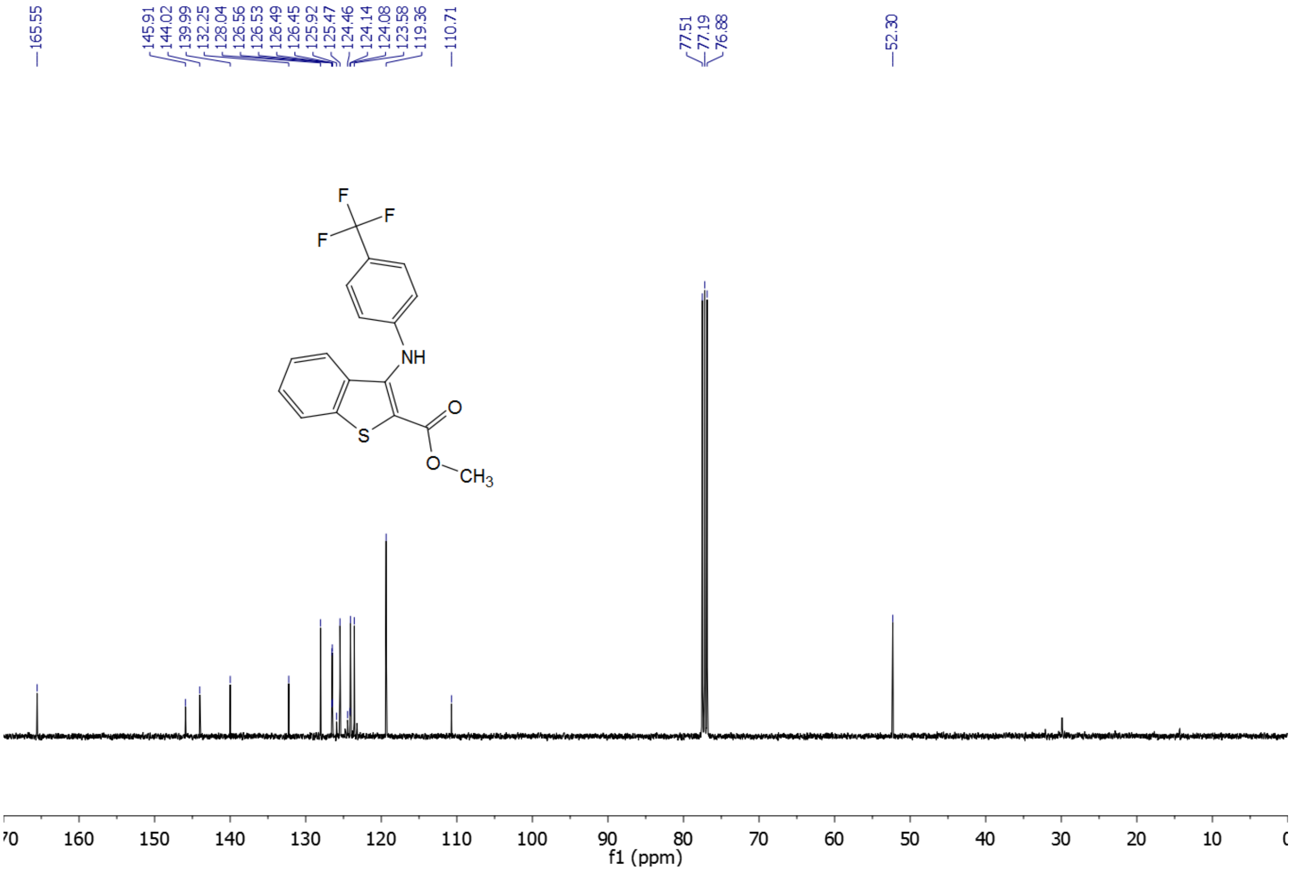

Figure S17. Methyl 3-{[4-(trifluoromethyl)phenyl]amino}-1-benzothiophene-2-carboxylate (3e). ^{13}C NMR spectrum (100 MHz, $CDCl_3$).

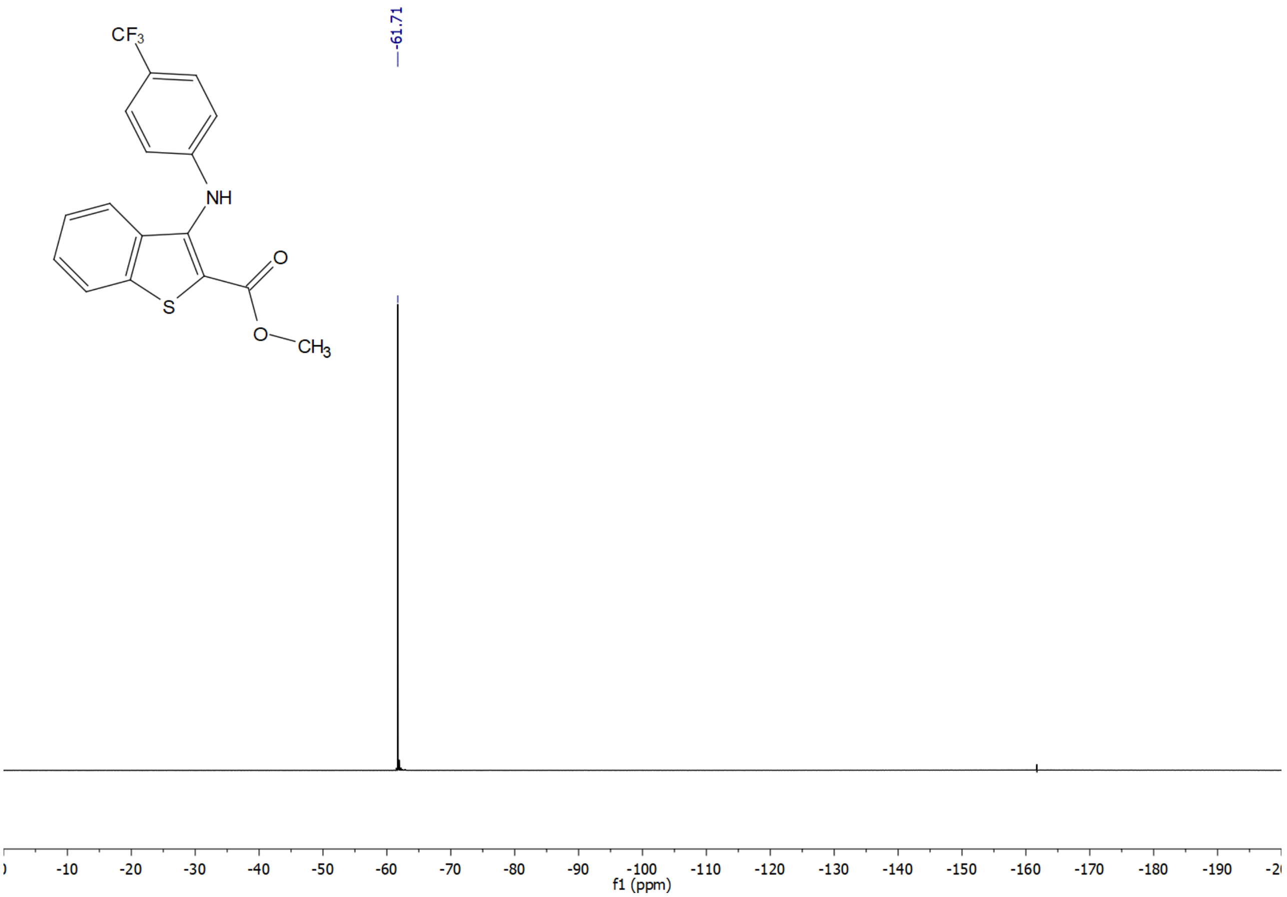

Figure S18. Methyl 3-{[4-(trifluoromethyl)phenyl]amino}-1-benzothiophene-2-carboxylate (3e). ^{19}**F NMR spectrum (100 MHz, $CDCl_3$).**

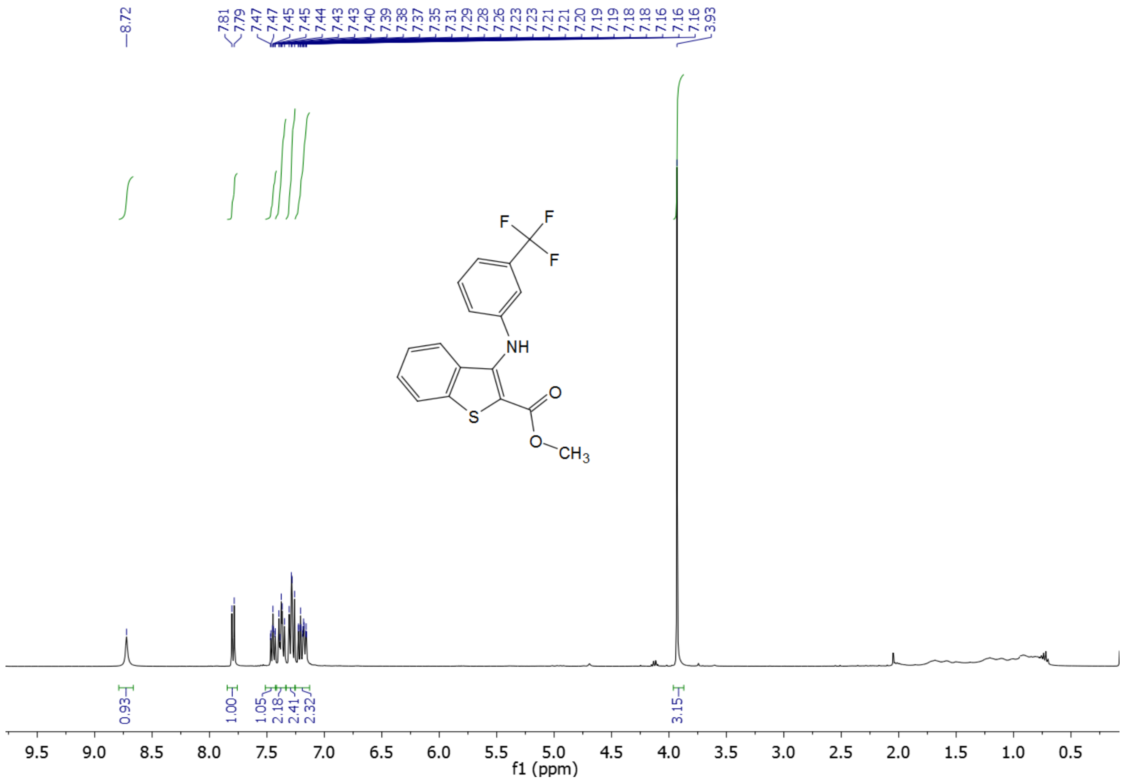

Figure S19. Methyl 3-{[3-(trifluoromethyl)phenyl]amino}-1-benzothiophene-2-carboxylate (3f). ^{1}**H NMR spectrum (400 MHz, $CDCl_3$).**

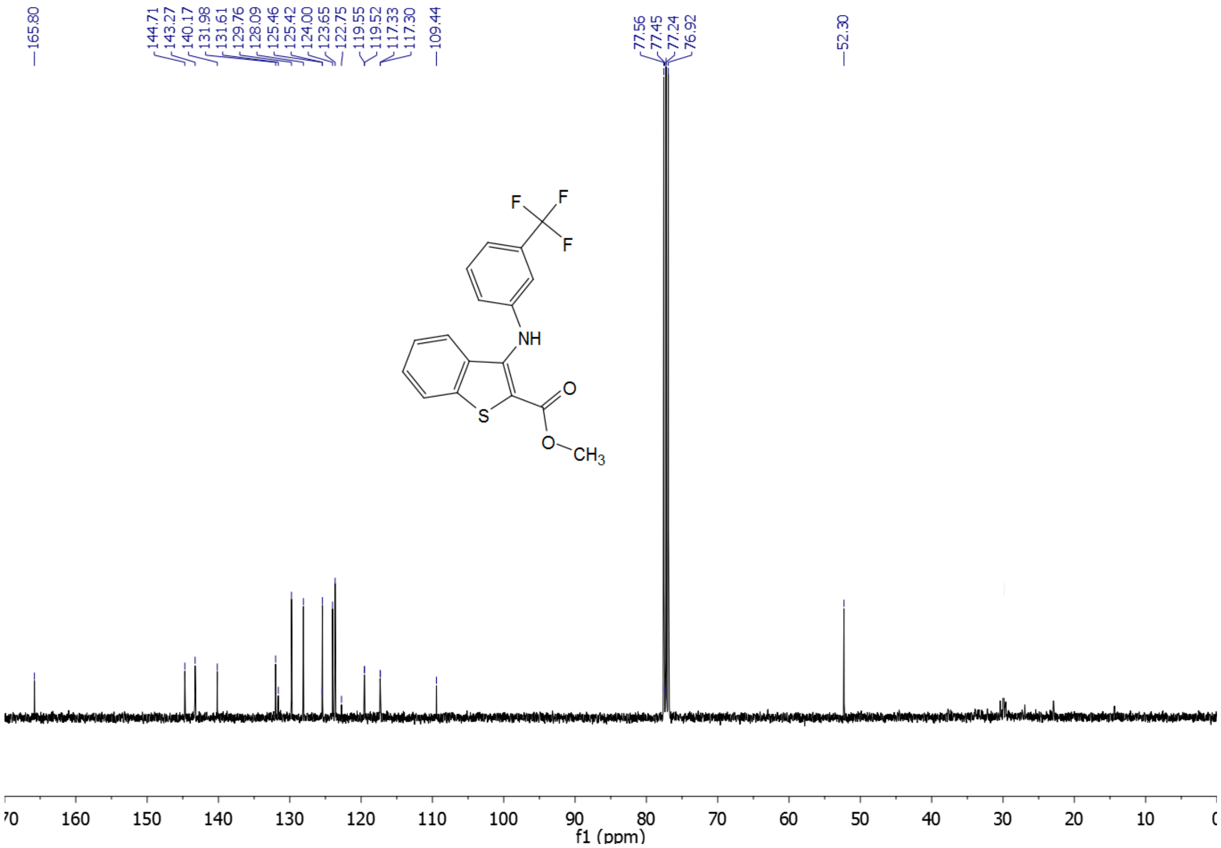

Figure S20. Methyl 3-{[3-(trifluoromethyl)phenyl]amino}-1-benzothiophene-2-carboxylate (3f). ^{13}C NMR spectrum (100 MHz, $CDCl_3$).

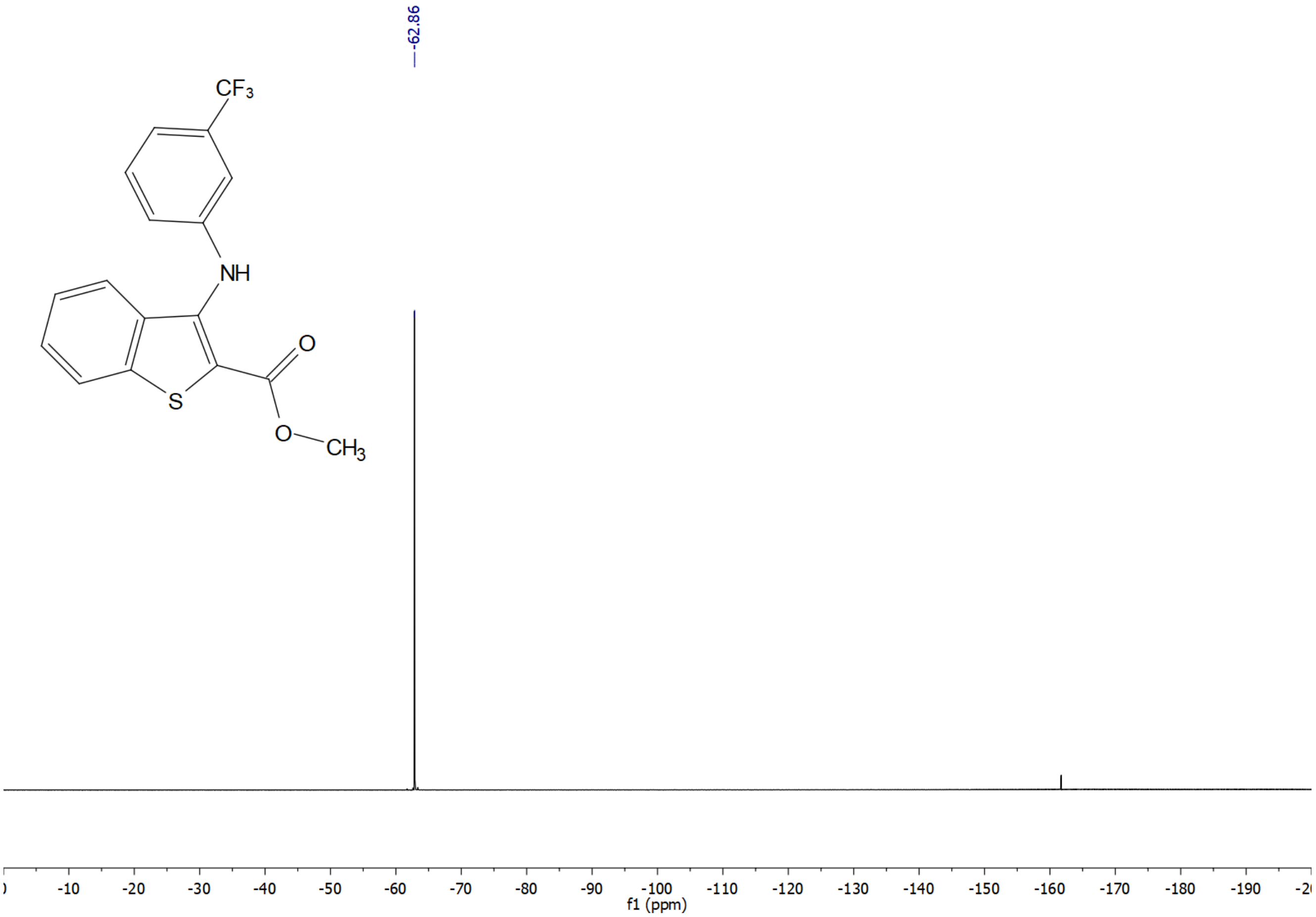

Figure S21. Methyl 3-{[3-(trifluoromethyl)phenyl]amino}-1-benzothiophene-2-carboxylate (3f). ^{19}F NMR spectrum (100 MHz, $CDCl_3$).

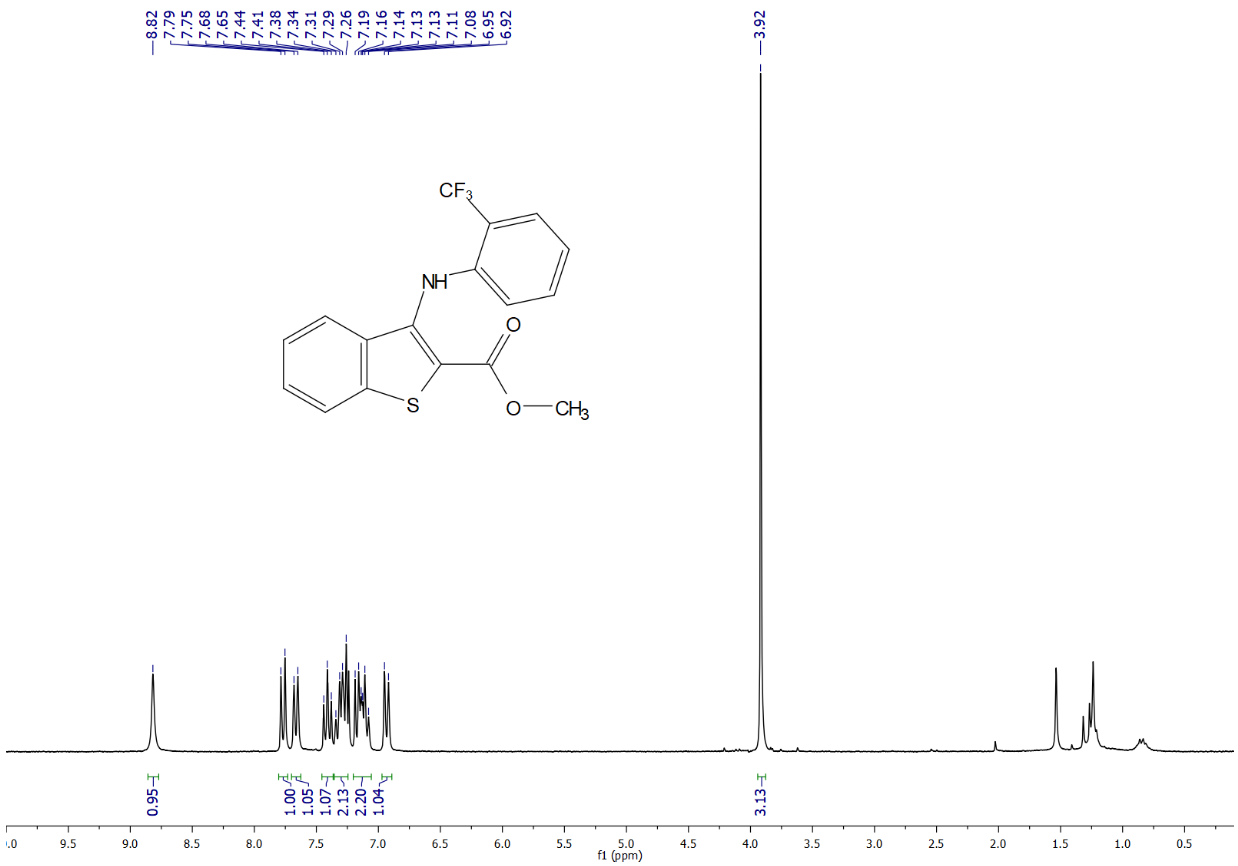

Figure S22. Methyl 3-{[2-(trifluoromethyl)phenyl]amino}-1-benzothiophene-2-carboxylate (3g). ^{1}H NMR spectrum (400 MHz, $CDCl_3$).

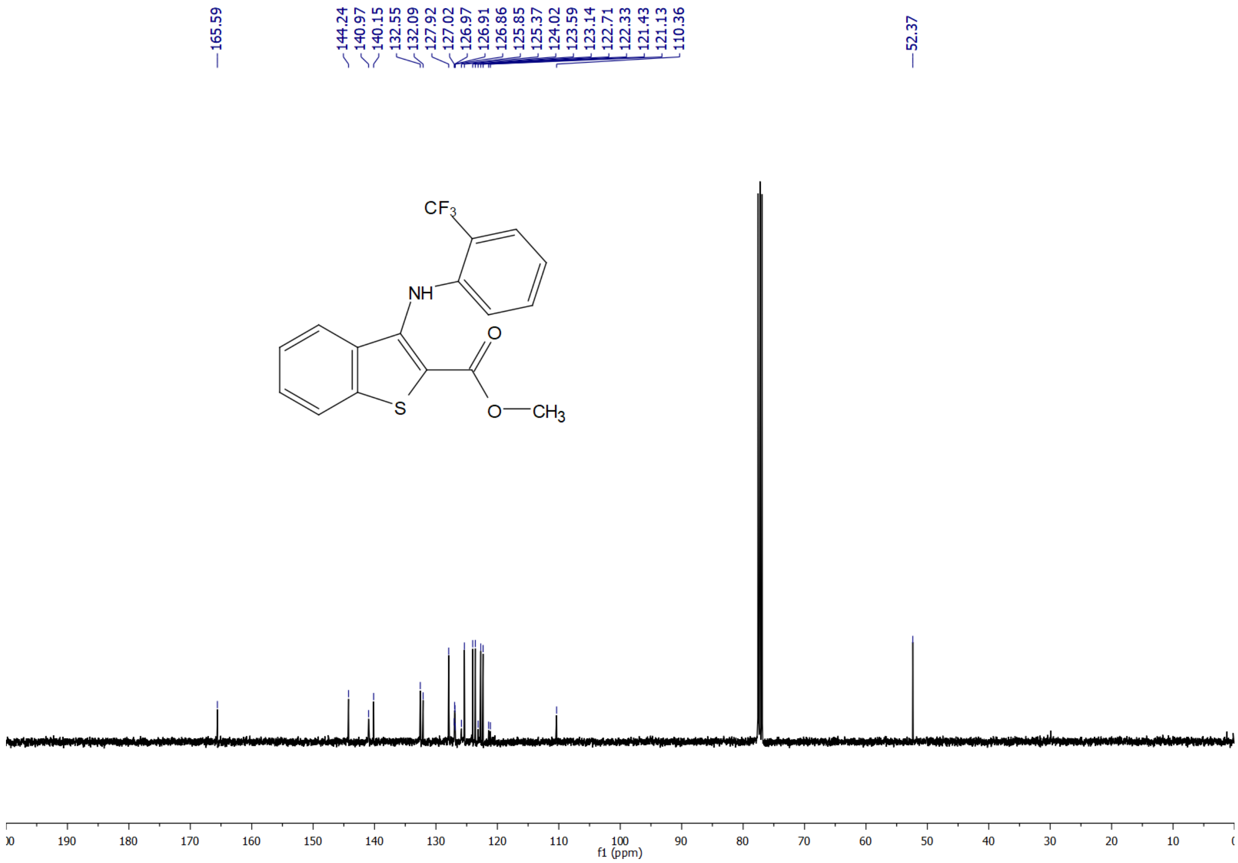

Figure S23. Methyl 3-{[2-(trifluoromethyl)phenyl]amino}-1-benzothiophene-2-carboxylate (3g). ^{13}C NMR spectrum (100 MHz, $CDCl_3$).

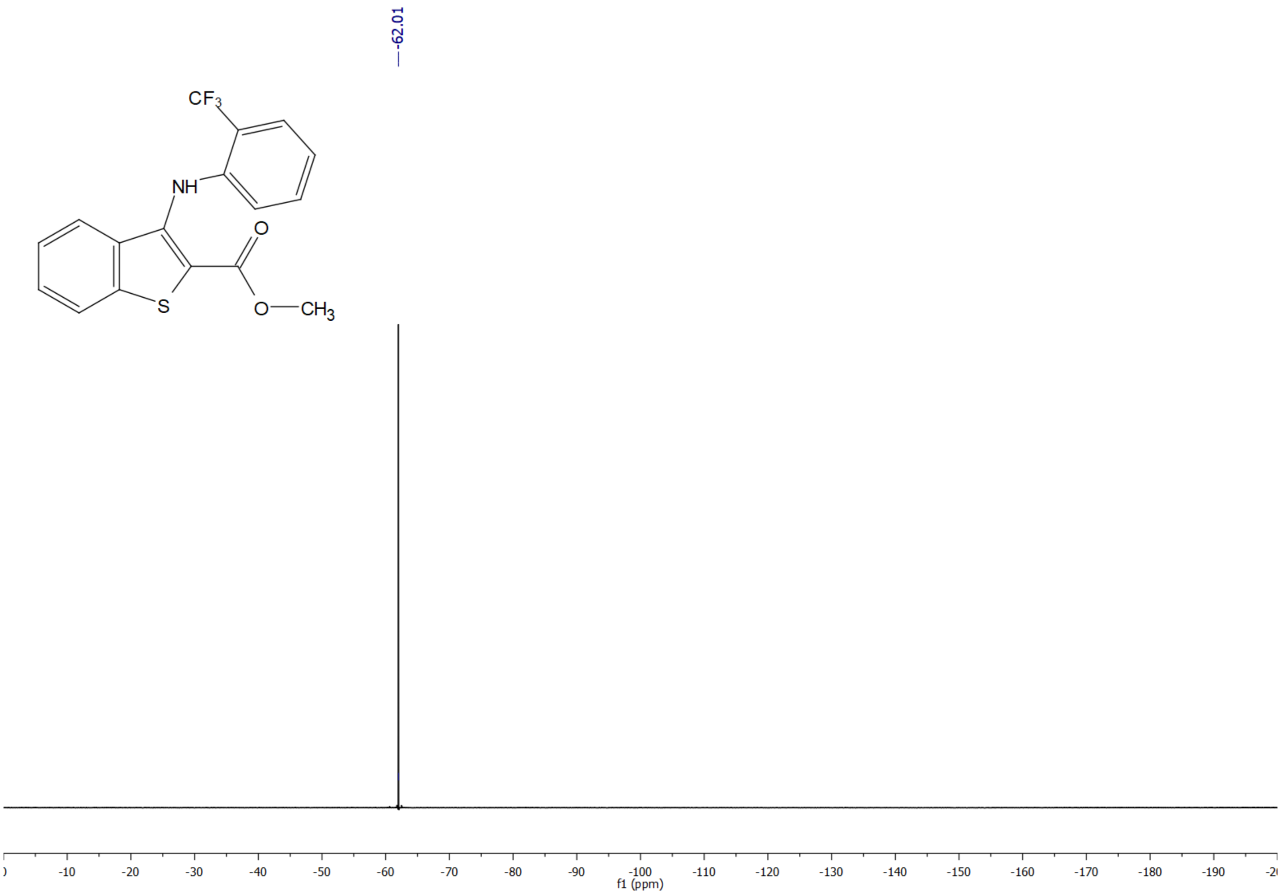

Figure S24. Methyl 3-{[2-(trifluoromethyl)phenyl]amino}-1-benzothiophene-2-carboxylate (3g). ^{19}F NMR spectrum (100 MHz, $CDCl_3$).

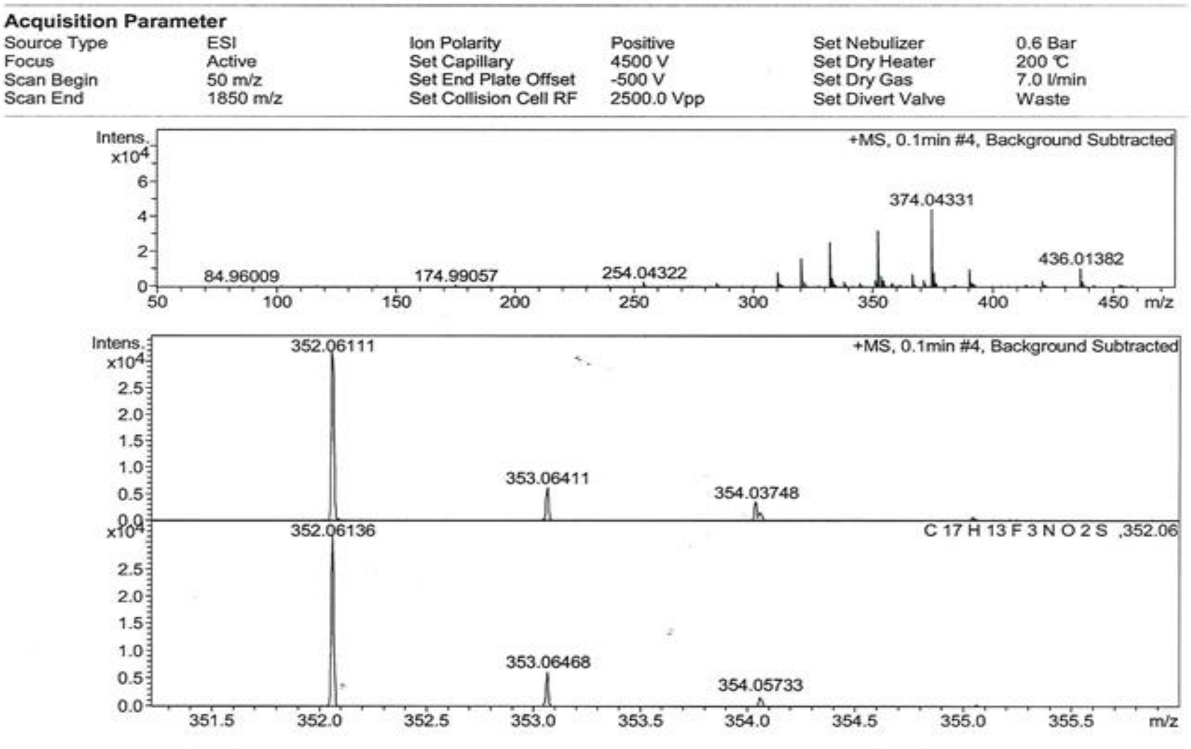

Meas. m/z	#	Formula	m/z	err [ppm]	mSigma	rdb	e⁻ Conf	N-Rule
352.06111	1	C 17 H 13 F 3 N O 2 S	352.06136	0.72	34.0	10.5	even	ok
374.04331	1	C 17 H 12 F 3 N Na O 2 S	374.04331	-0.02	10.6	10.5	even	ok
390.01729	1	C 17 H 12 F 3 K N O 2 S	390,01724	-0.14	120.7	10.5	even	ok

Figure S25. Methyl 3-{[2-(trifluoromethyl)phenyl]amino}-1-benzothiophene-2-carboxylate (3g). HRMS (ESI).

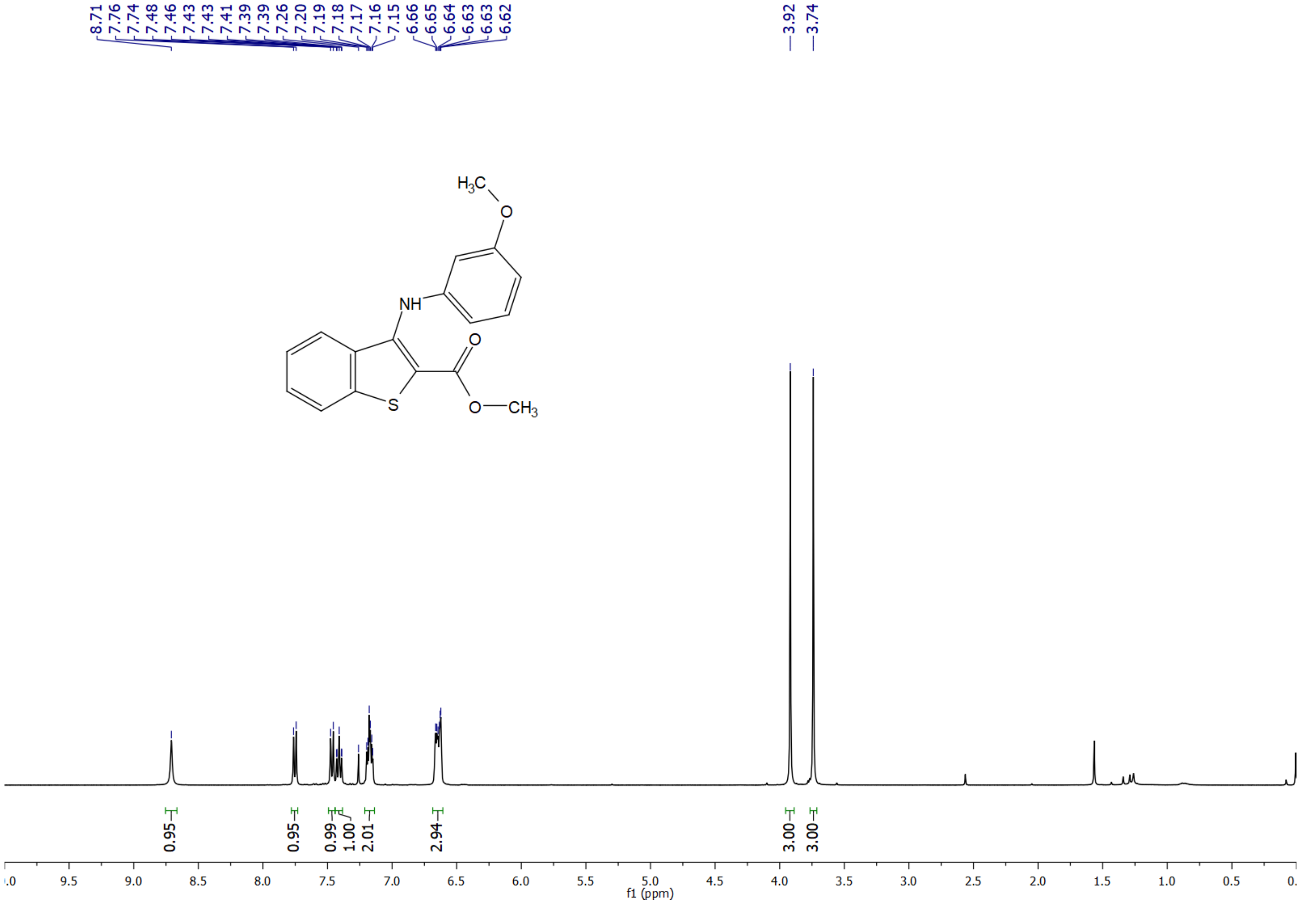

Figure S26. Methyl 3-[(3-methoxyphenyl)amino]-1-benzothiophene-2-carboxylate (3h). ^{1}H NMR spectrum (400 MHz, $CDCl_3$).

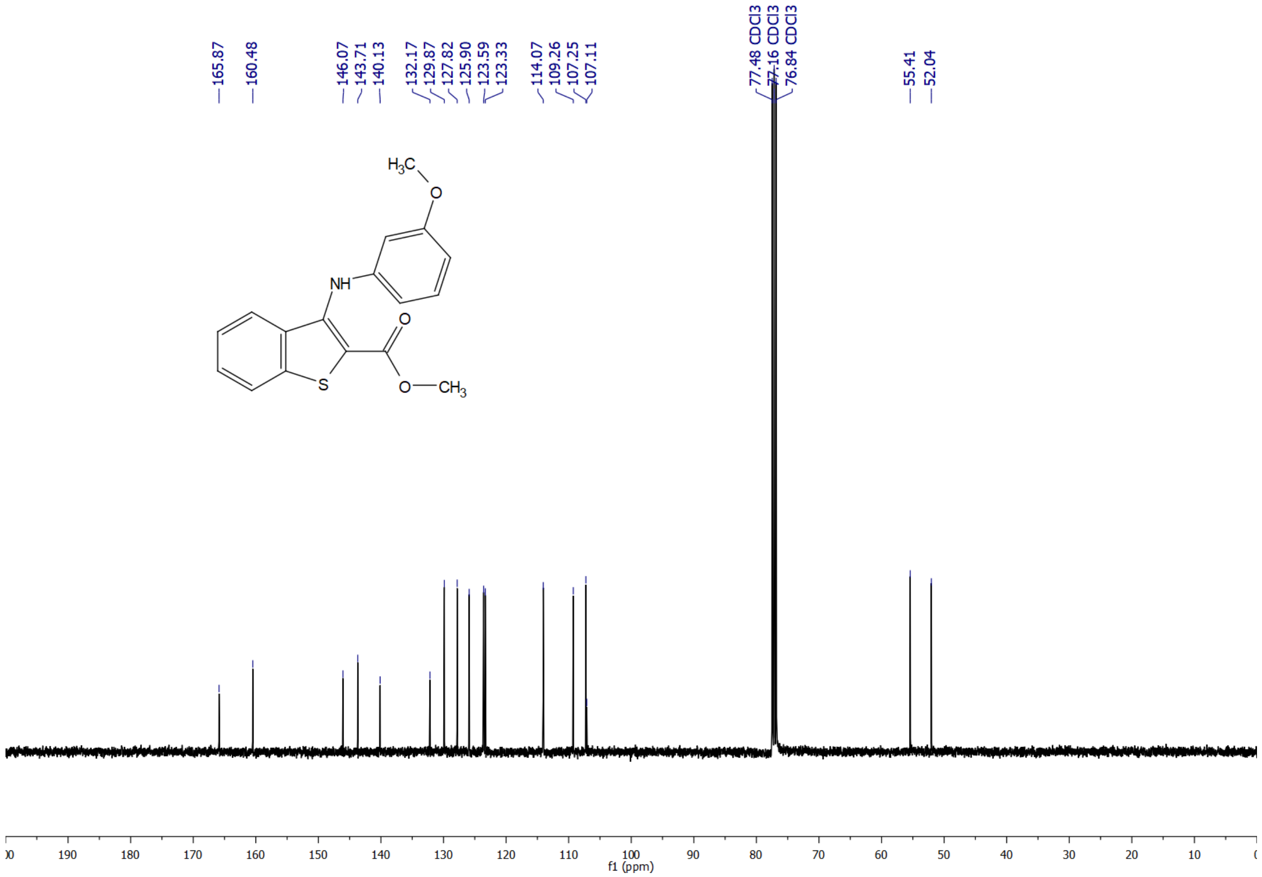

Figure S27. Methyl 3-[(3-methoxyphenyl)amino]-1-benzothiophene-2-carboxylate (3h). ^{13}C NMR spectrum (100 MHz, $CDCl_3$).

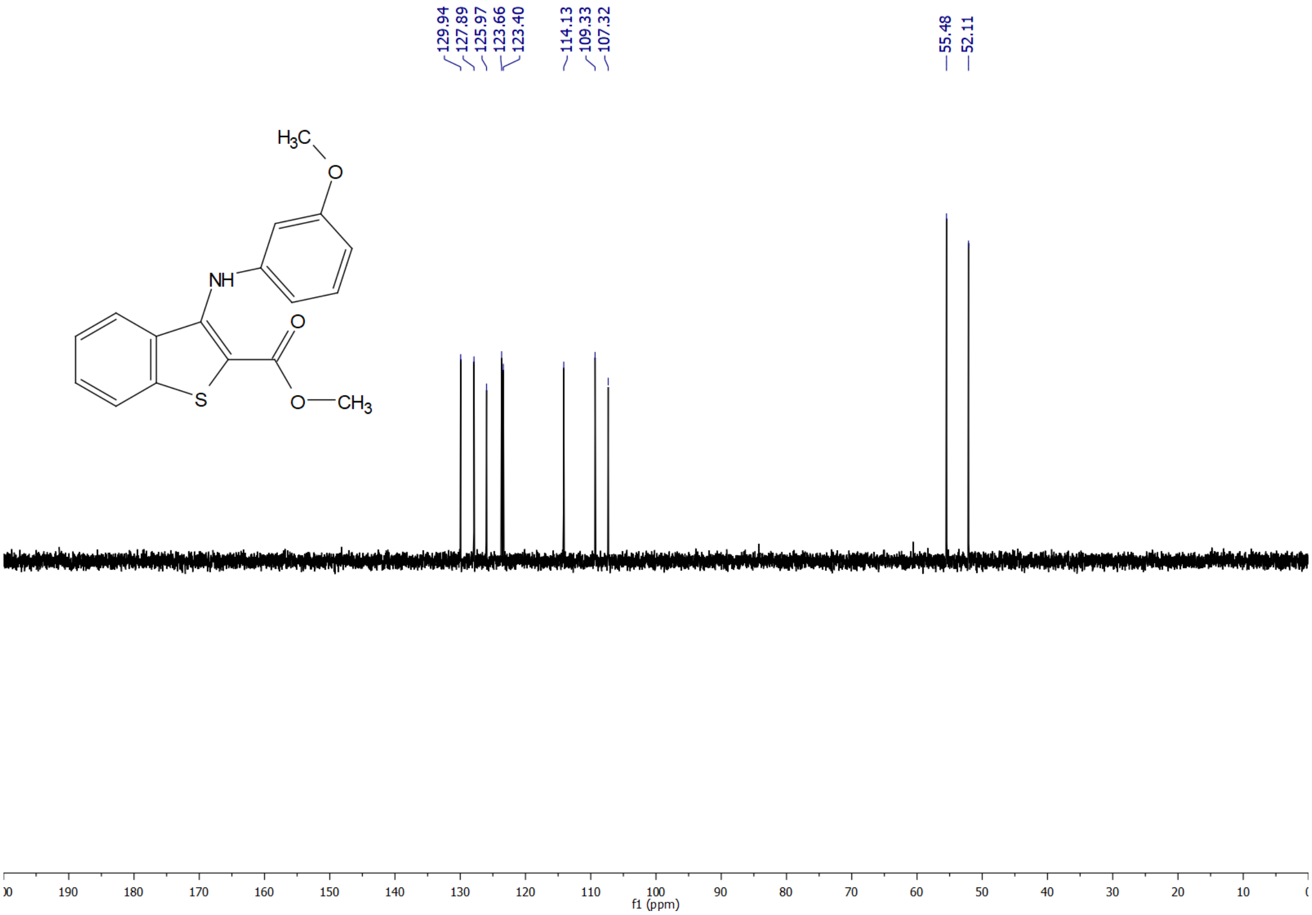

Figure S28. Methyl 3-[(3-methoxyphenyl)amino]-1-benzothiophene-2-carboxylate (3h). DEPT135 spectrum (100 MHz, $CDCl_3$).

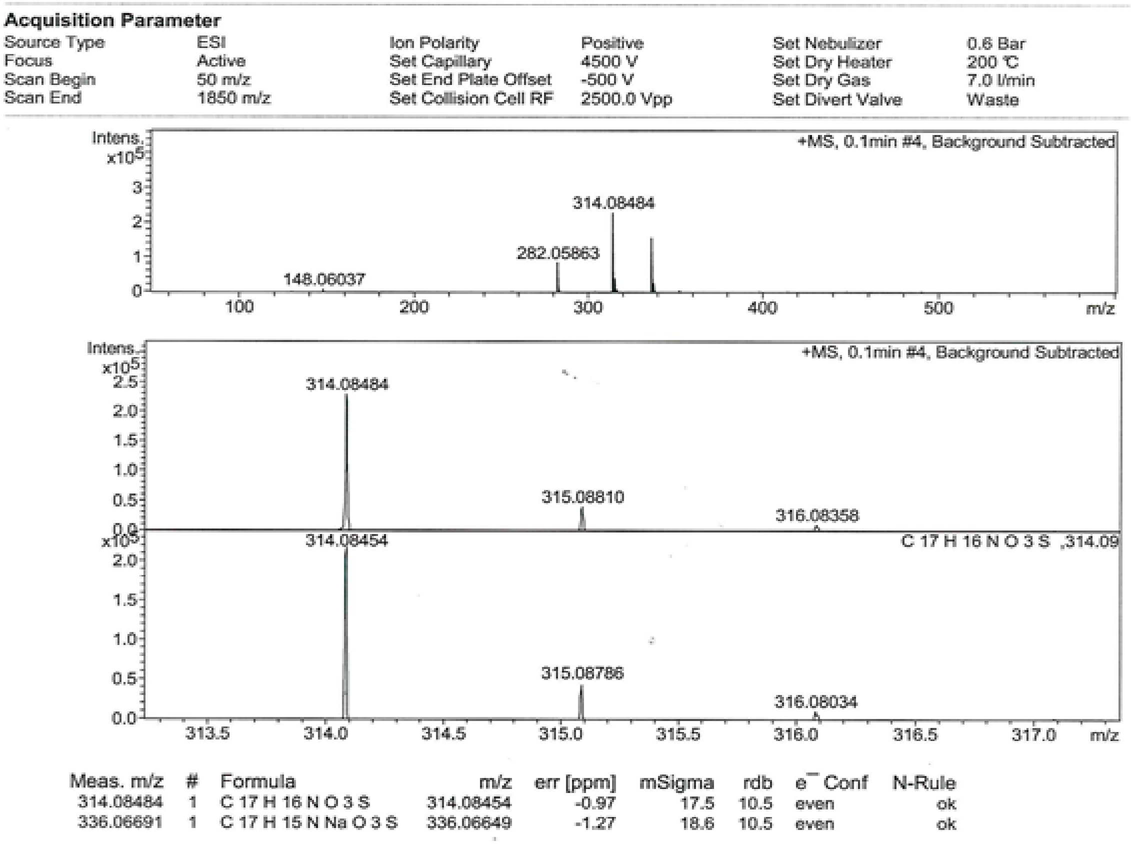

Figure S29. Methyl 3-[(3-methoxyphenyl)amino]-1-benzothiophene-2-carboxylate (3h). HRMS (ESI).

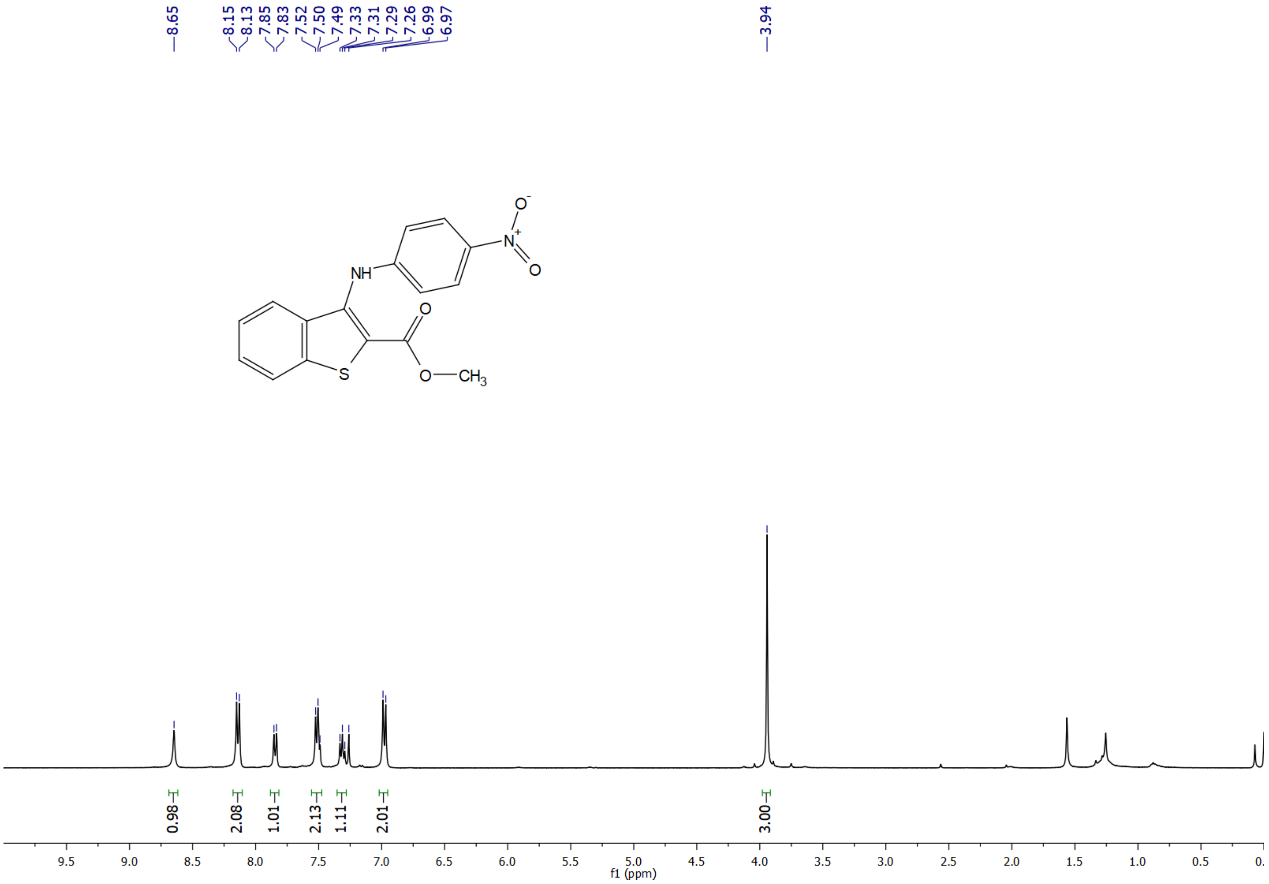

Figure S30. Methyl 3-[(4-nitrophenyl)amino]-1-benzothiophene-2-carboxylate (3i). ^{1}H NMR spectrum (400 MHz, $CDCl_3$).

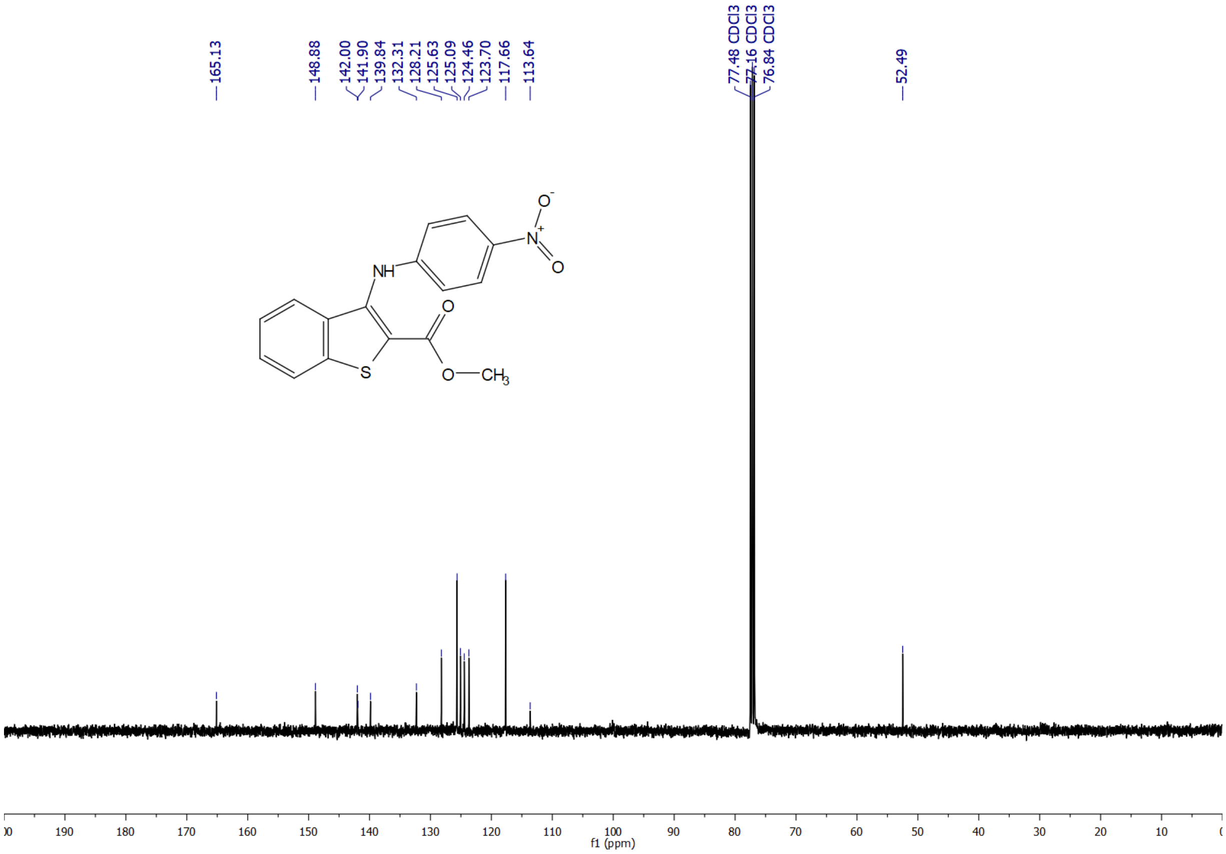

Figure S31. Methyl 3-[(4-nitrophenyl)amino]-1-benzothiophene-2-carboxylate (3i). ^{13}C NMR spectrum (100 MHz, $CDCl_3$).

Acquisition Parameter

Source Type	ESI	Ion Polarity	Positive	Set Nebulizer	0.6 Bar
Focus	Active	Set Capillary	4500 V	Set Dry Heater	200 ℃
Scan Begin	50 m/z	Set End Plate Offset	-500 V	Set Dry Gas	7.0 l/min
Scan End	1850 m/z	Set Collision Cell RF	2500.0 Vpp	Set Divert Valve	Waste

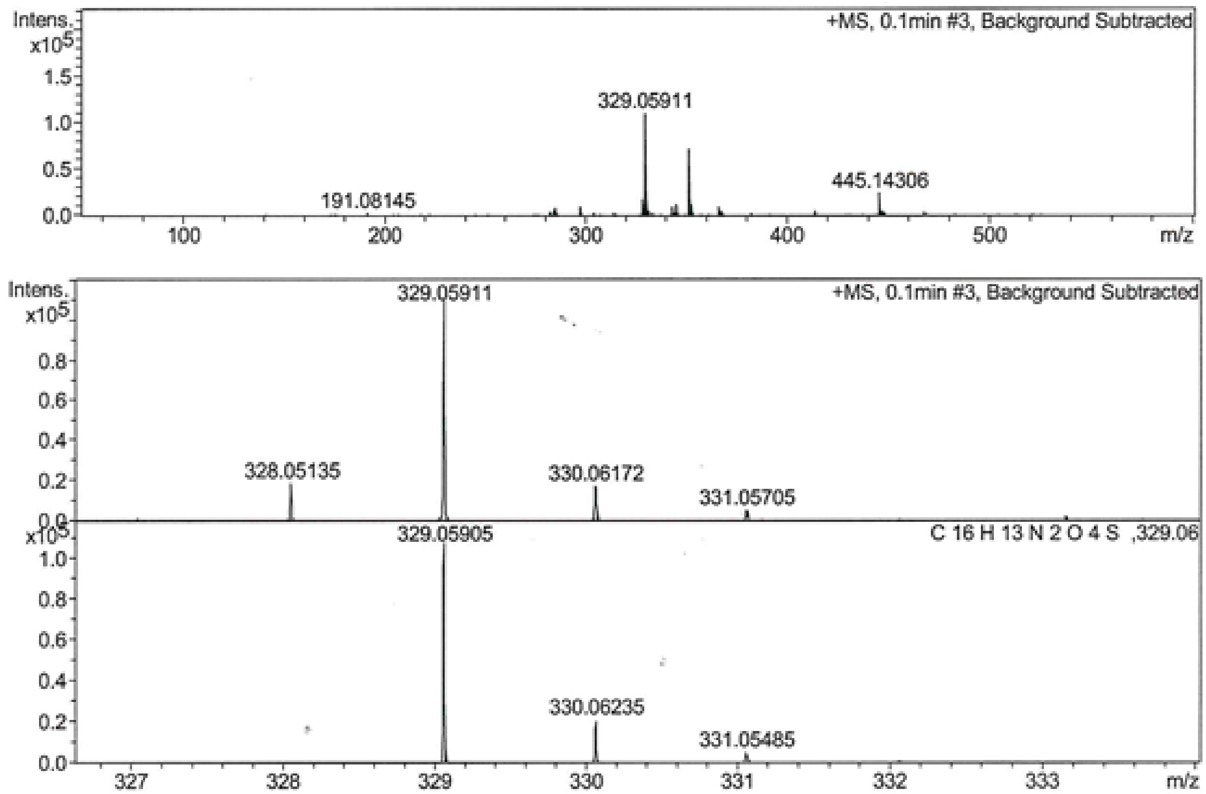

Meas. m/z	#	Formula	m/z	err [ppm]	mSigma	rdb	e⁻ Conf	N-Rule
329.05911	1	C 16 H 13 N 2 O 4 S	329.05905	-0.16	18.6	11.5	even	ok
351.04112	1	C 16 H 12 N 2 Na O 4 S	351.04100	-0.35	7.8	11.5	even	ok

Figure S32. Methyl 3-[(4-nitrophenyl)amino]-1-benzothiophene-2-carboxylate (3i). HRMS (ESI).

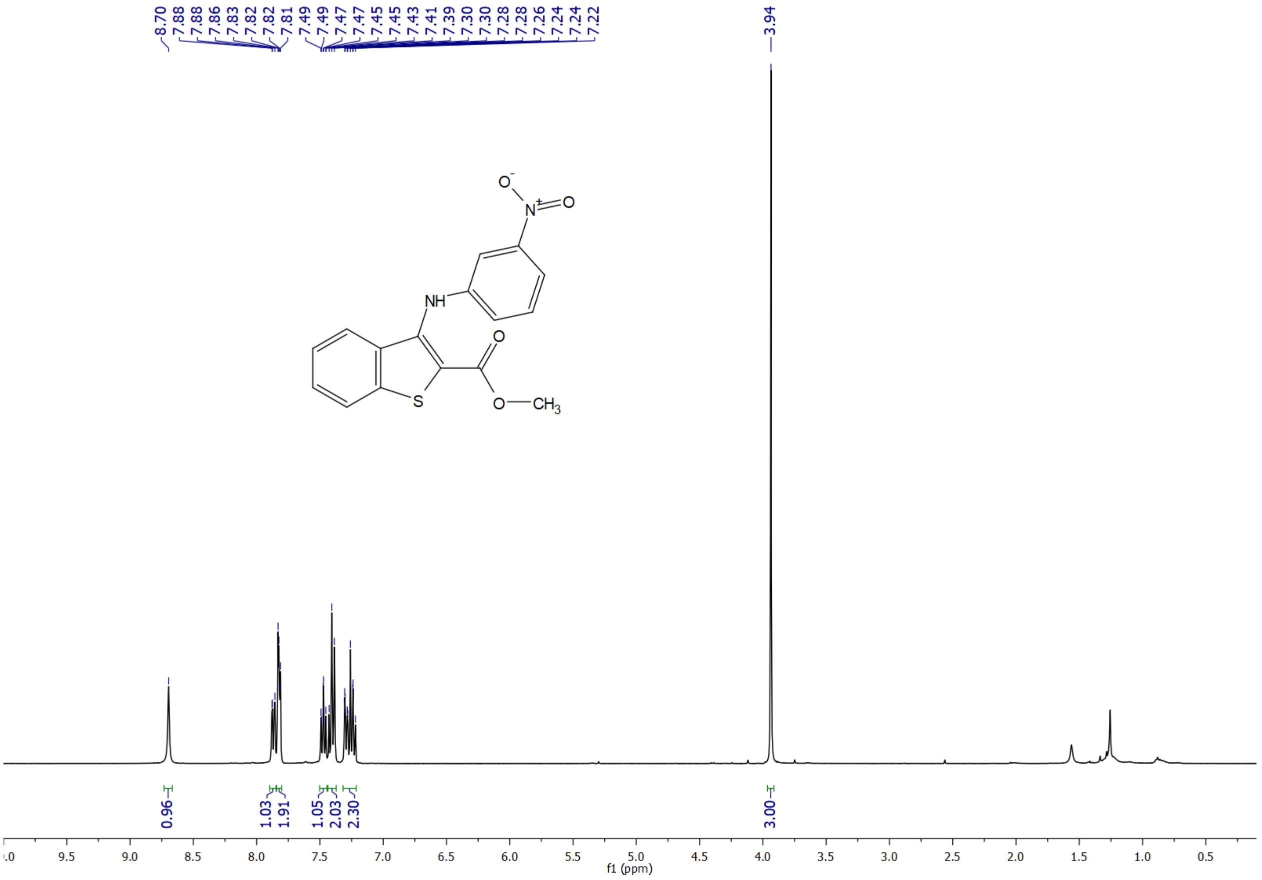

Figure S33. Methyl 3-[(3-nitrophenyl)amino]-1-benzothiophene-2-carboxylate (3j). ^{1}H NMR spectrum (400 MHz, $CDCl_3$).

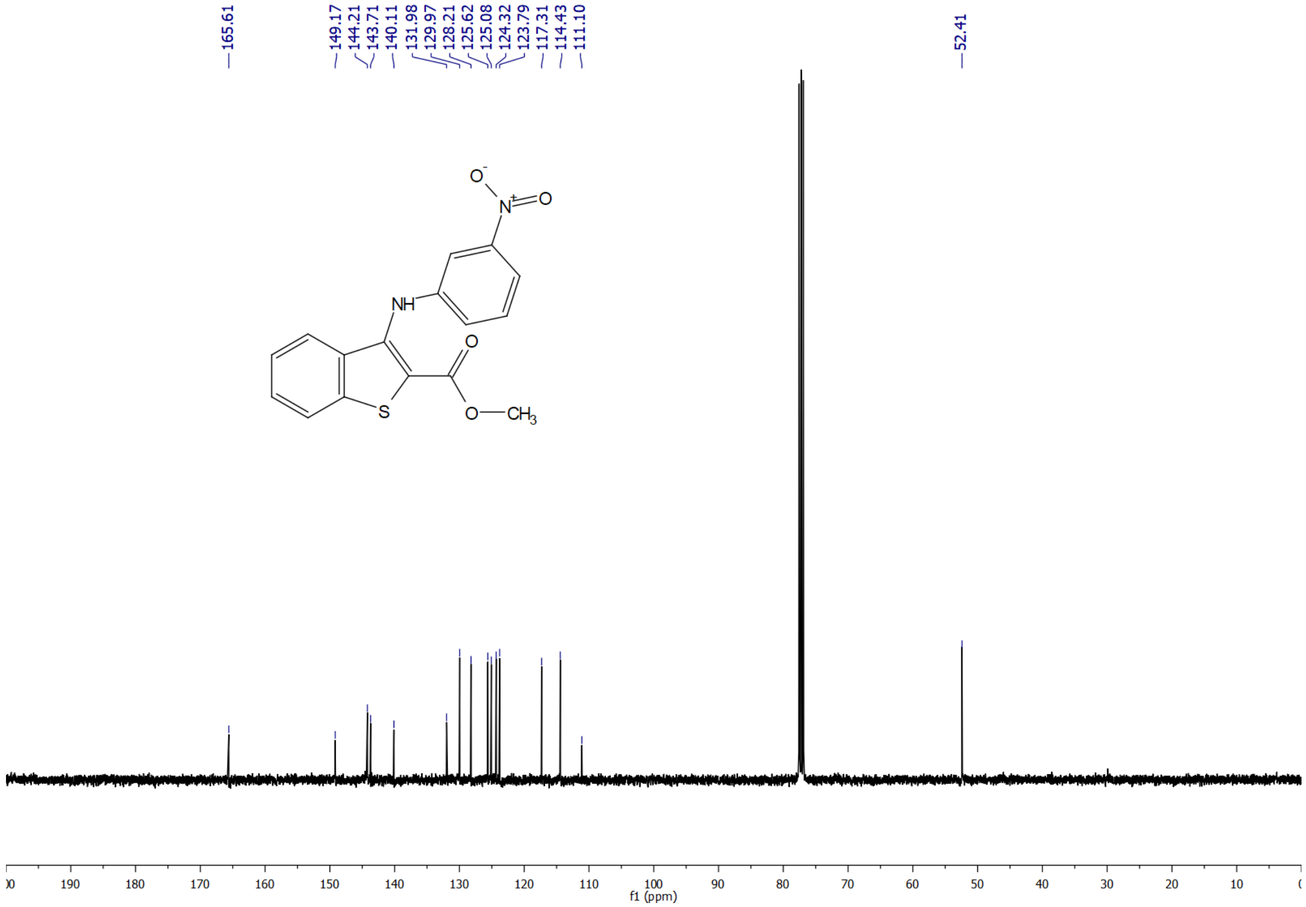

Figure S34. Methyl 3-[(3-nitrophenyl)amino]-1-benzothiophene-2-carboxylate (3j). ^{13}C NMR spectrum (100 MHz, $CDCl_3$).

Acquisition Parameter

Source Type	ESI	Ion Polarity	Positive	Set Nebulizer	0.6 Bar
Focus	Active	Set Capillary	4500 V	Set Dry Heater	200 °C
Scan Begin	50 m/z	Set End Plate Offset	-500 V	Set Dry Gas	7.0 l/min
Scan End	1850 m/z	Set Collision Cell RF	2500.0 Vpp	Set Divert Valve	Waste

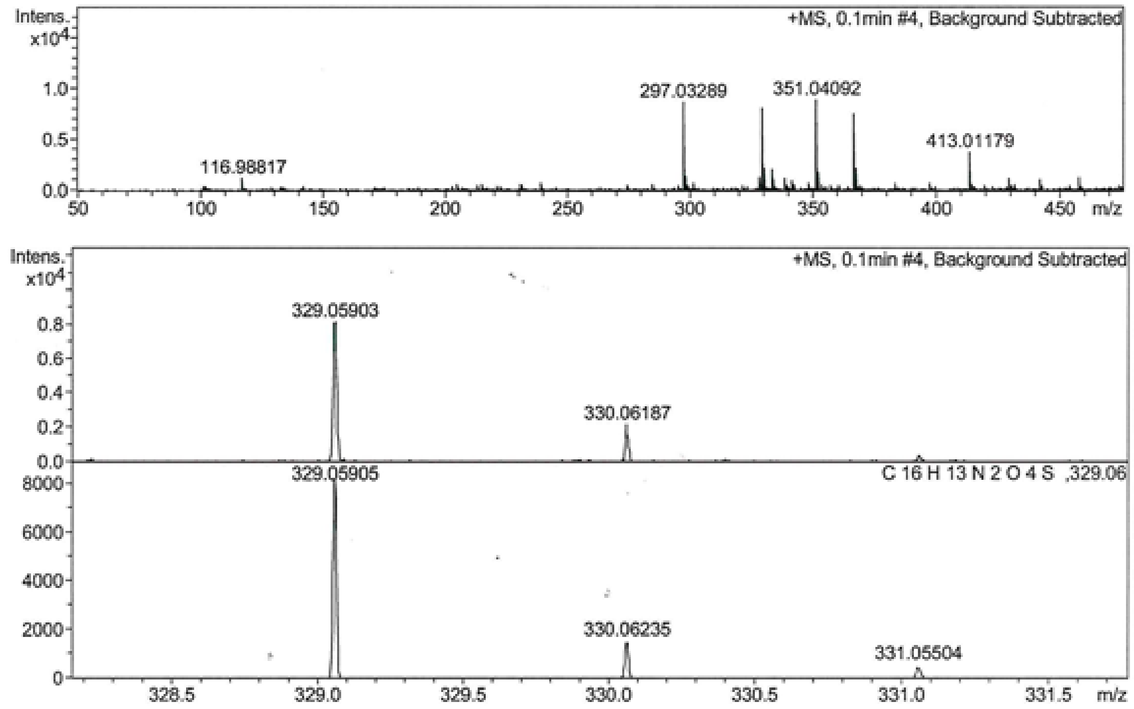

Meas. m/z	#	Formula	m/z	err [ppm]	mSigma	rdb	e⁻ Conf	N-Rule
297.03289	1	C 15 H 9 N 2 O 3 S	297.03284	-0.17	33.9	12.5	even	ok
329.05903	1	C 16 H 13 N 2 O 4 S	329.05905	0.07	53.5	11.5	even	ok
351.04092	1	C 16 H 12 N 2 Na O 4 S	351.04100	0.22	100.7	11.5	even	ok

Figure S35. Methyl 3-[(3-nitrophenyl)amino]-1-benzothiophene-2-carboxylate (3j). HRMS (ESI).

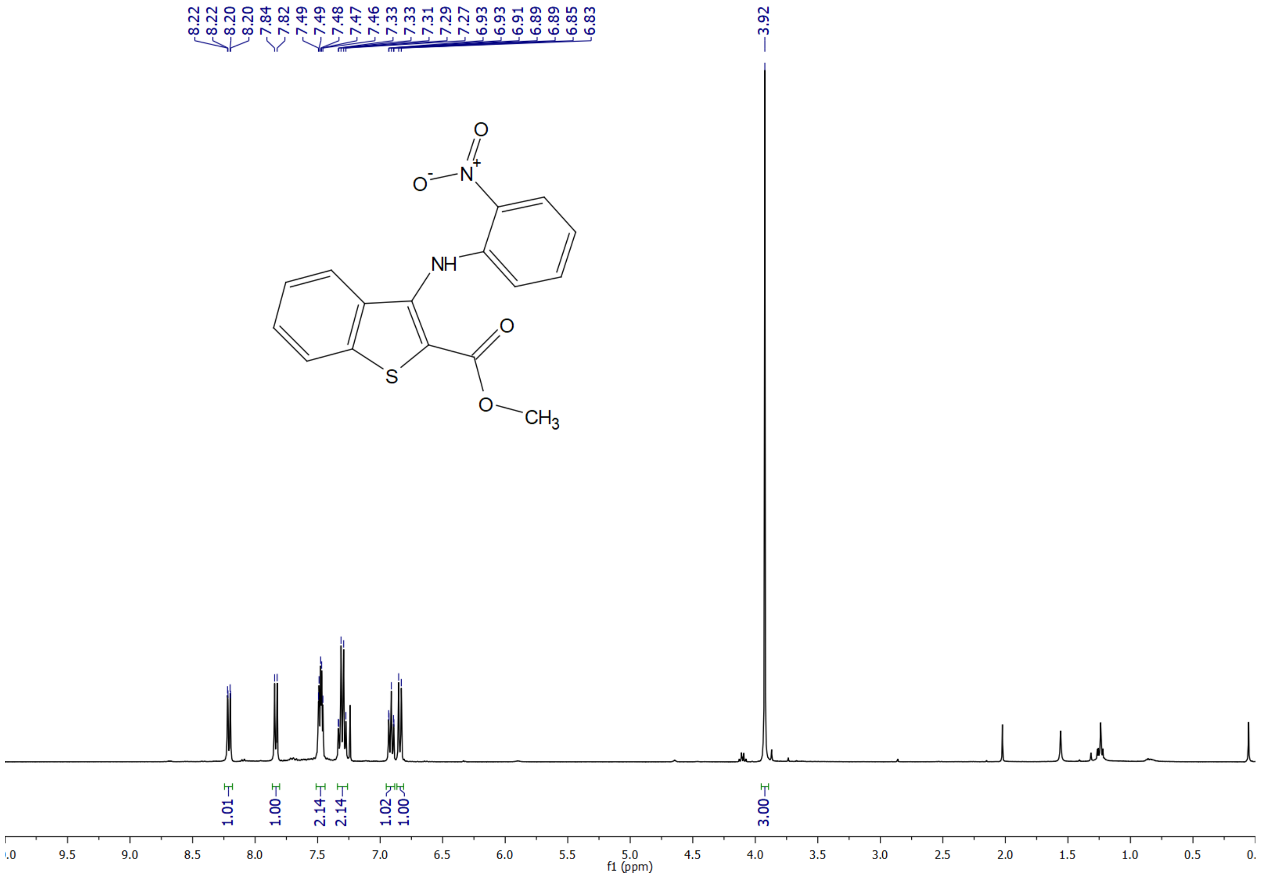

Figure S36. Methyl 3-[(2-nitrophenyl)amino]-1-benzothiophene-2-carboxylate (3k). ^{1}H NMR spectrum (400 MHz, $CDCl_3$).

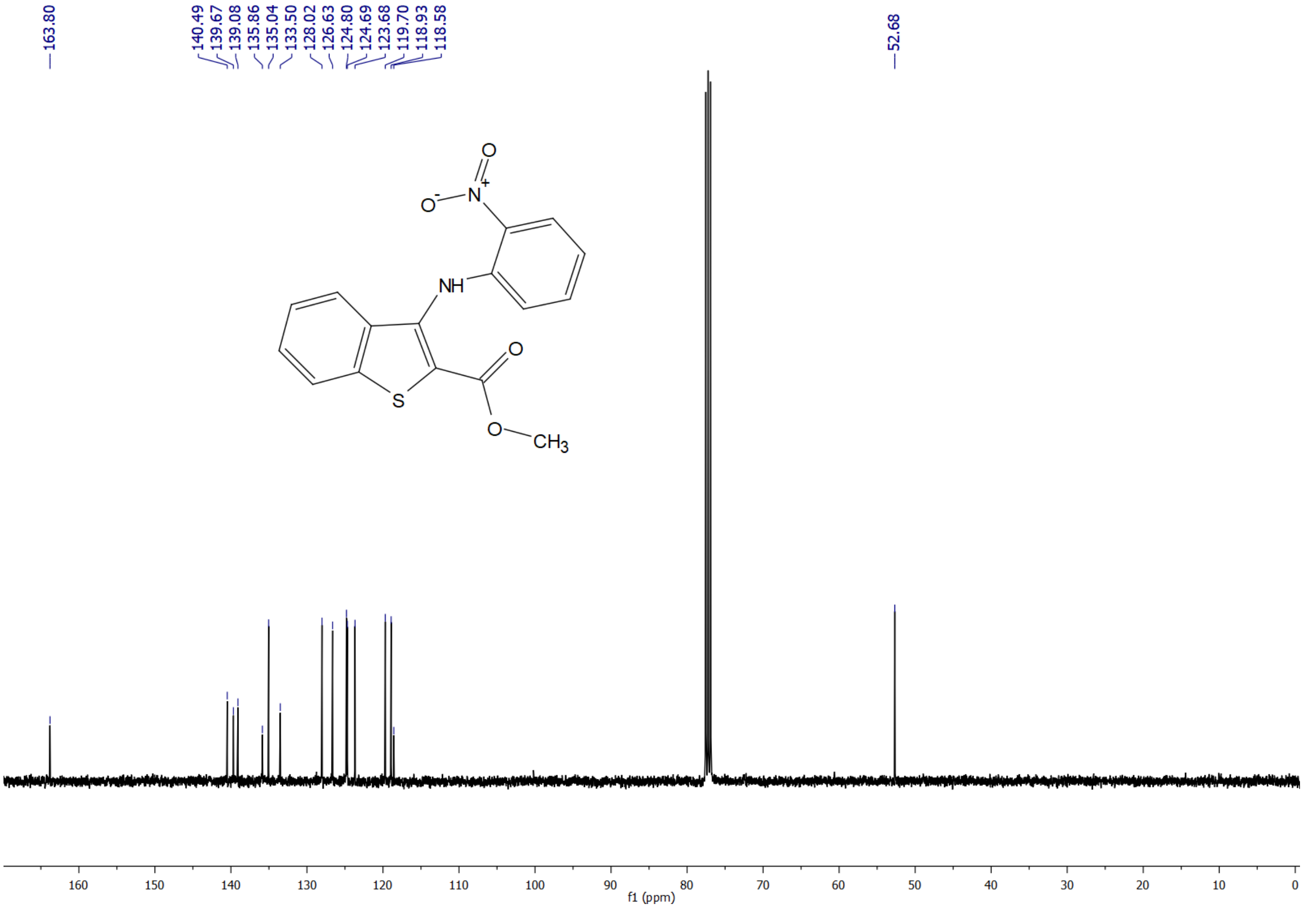

Figure S37. Methyl 3-[(2-nitrophenyl)amino]-1-benzothiophene-2-carboxylate (3k). ^{13}C NMR spectrum (100 MHz, $CDCl_3$).

Acquisition Parameter

Source Type	ESI	Ion Polarity	Positive	Set Nebulizer	0.6 Bar
Focus	Active	Set Capillary	4500 V	Set Dry Heater	200 °C
Scan Begin	50 m/z	Set End Plate Offset	-500 V	Set Dry Gas	7.0 l/min
Scan End	1850 m/z	Set Collision Cell RF	2500.0 Vpp	Set Divert Valve	Waste

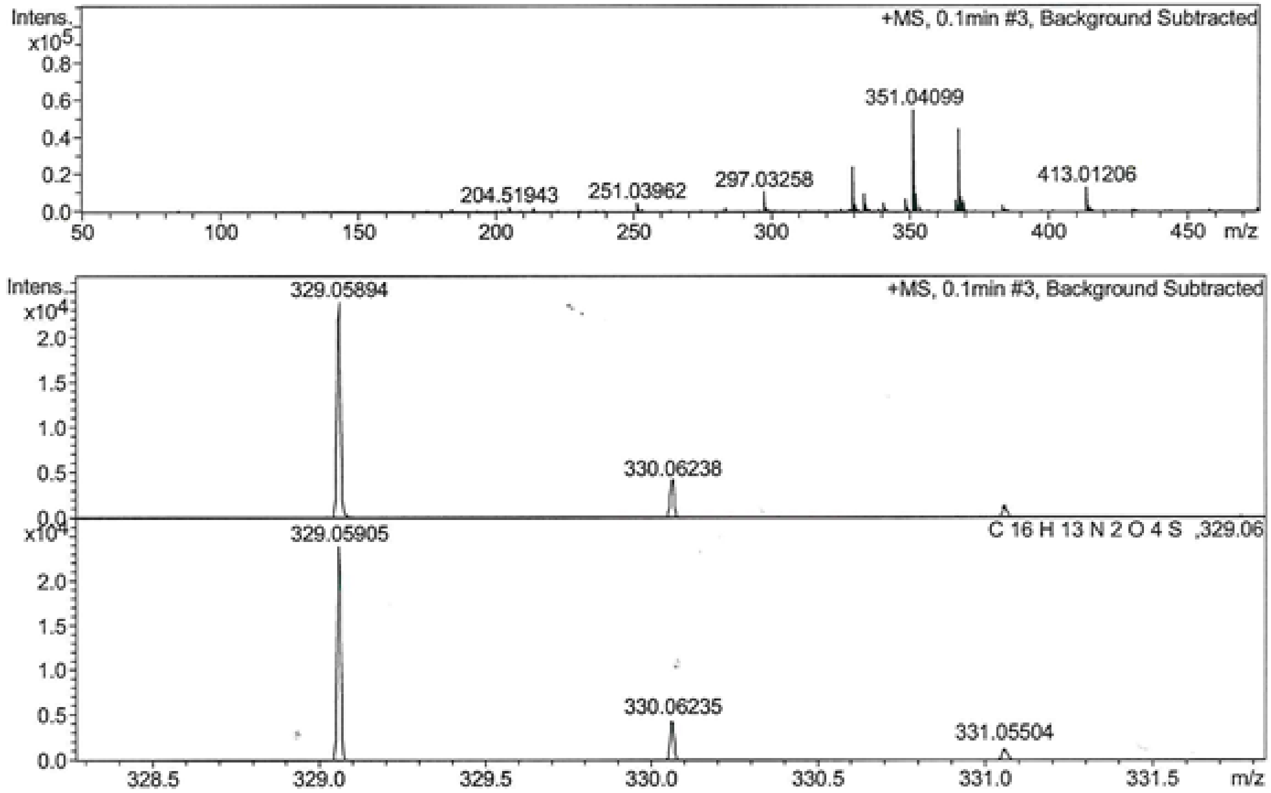

Meas. m/z	#	Formula	m/z	err [ppm]	mSigma	rdb	e⁻ Conf	N-Rule
329.05894	1	C 16 H 13 N 2 O 4 S	329.05905	0.36	36.6	11.5	even	ok
351.04099	1	C 16 H 12 N 2 Na O 4 S	351.04100	0.03	12.7	11.5	even	ok
367.01501	1	C 16 H 12 K N 2 O 4 S	367.01494	-0.20	18.9	11.5	even	ok

Figure S38. Methyl 3-[(2-nitrophenyl)amino]-1-benzothiophene-2-carboxylate (3k). HRMS (ESI).

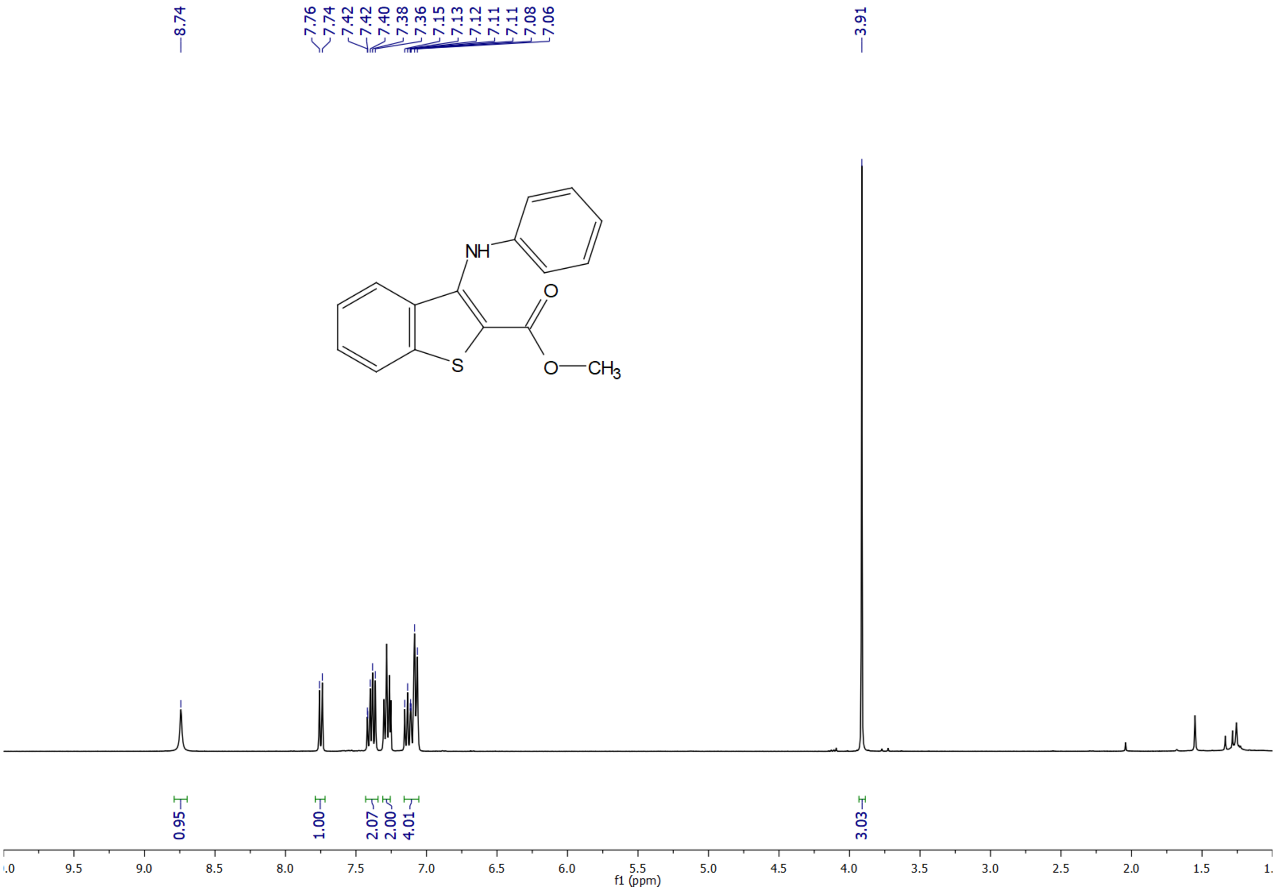

Figure S39. Methyl 3-(phenylamino)-1-benzothiophene-2-carboxylate (3l). ^{1}H NMR spectrum (400 MHz, $CDCl_3$).

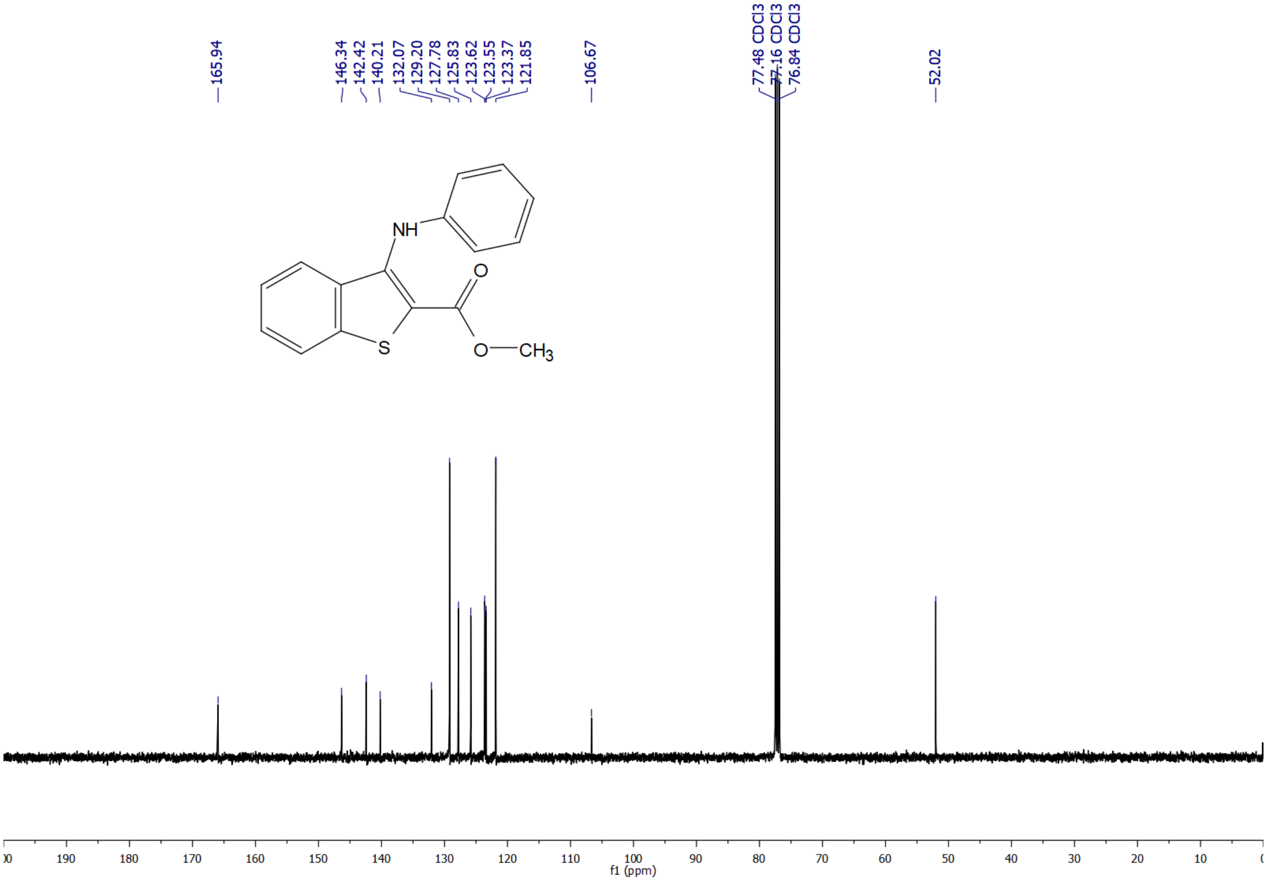

Figure S40. Methyl 3-(phenylamino)-1-benzothiophene-2-carboxylate (3l). ^{13}C NMR spectrum (100 MHz, $CDCl_3$).

Acquisition Parameter

Source Type	ESI	Ion Polarity	Positive	Set Nebulizer	0.6 Bar
Focus	Active	Set Capillary	4500 V	Set Dry Heater	200 °C
Scan Begin	50 m/z	Set End Plate Offset	-500 V	Set Dry Gas	7.0 l/min
Scan End	1850 m/z	Set Collision Cell RF	2500.0 Vpp	Set Divert Valve	Waste

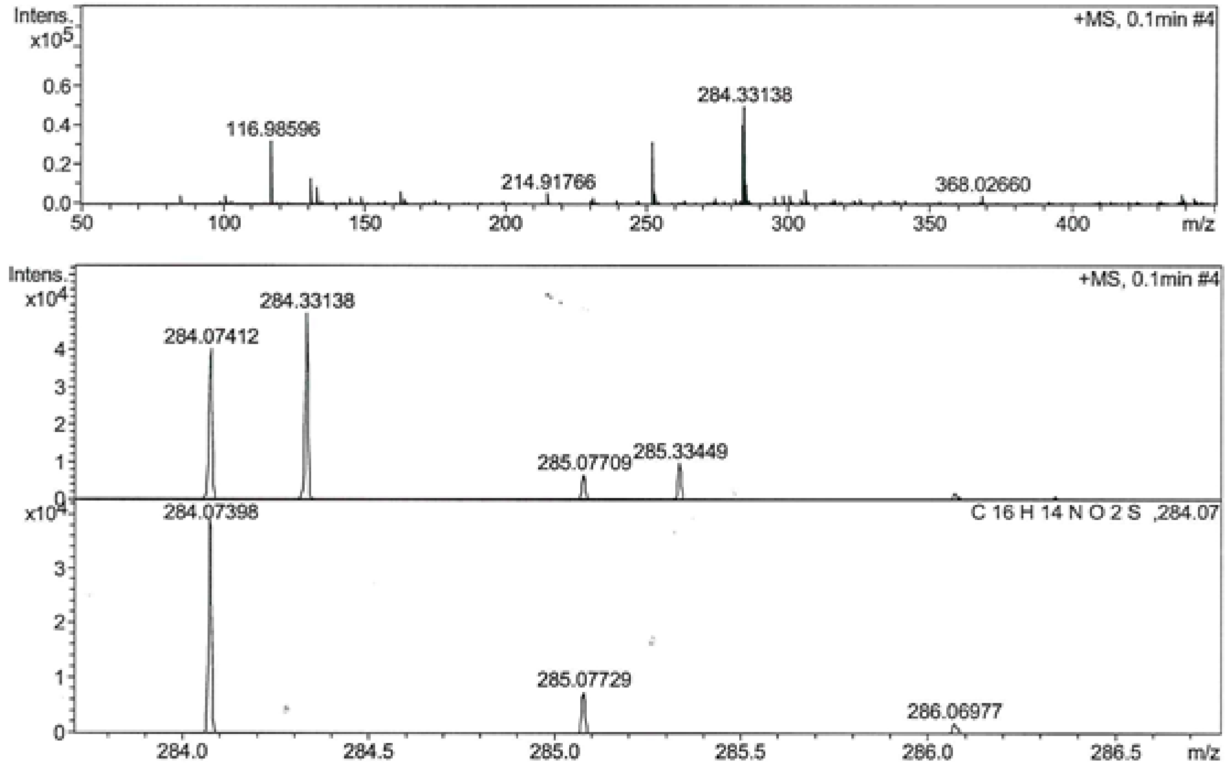

Meas. m/z	#	Formula	m/z	err [ppm]	mSigma	rdb	e⁻ Conf	N-Rule
284.07412	1	C 16 H 14 N O 2 S	284.07398	-0.52	34.1	10.5	even	ok

Figure S41. Methyl 3-(phenylamino)-1-benzothiophene-2-carboxylate (3l). HRMS (ESI).

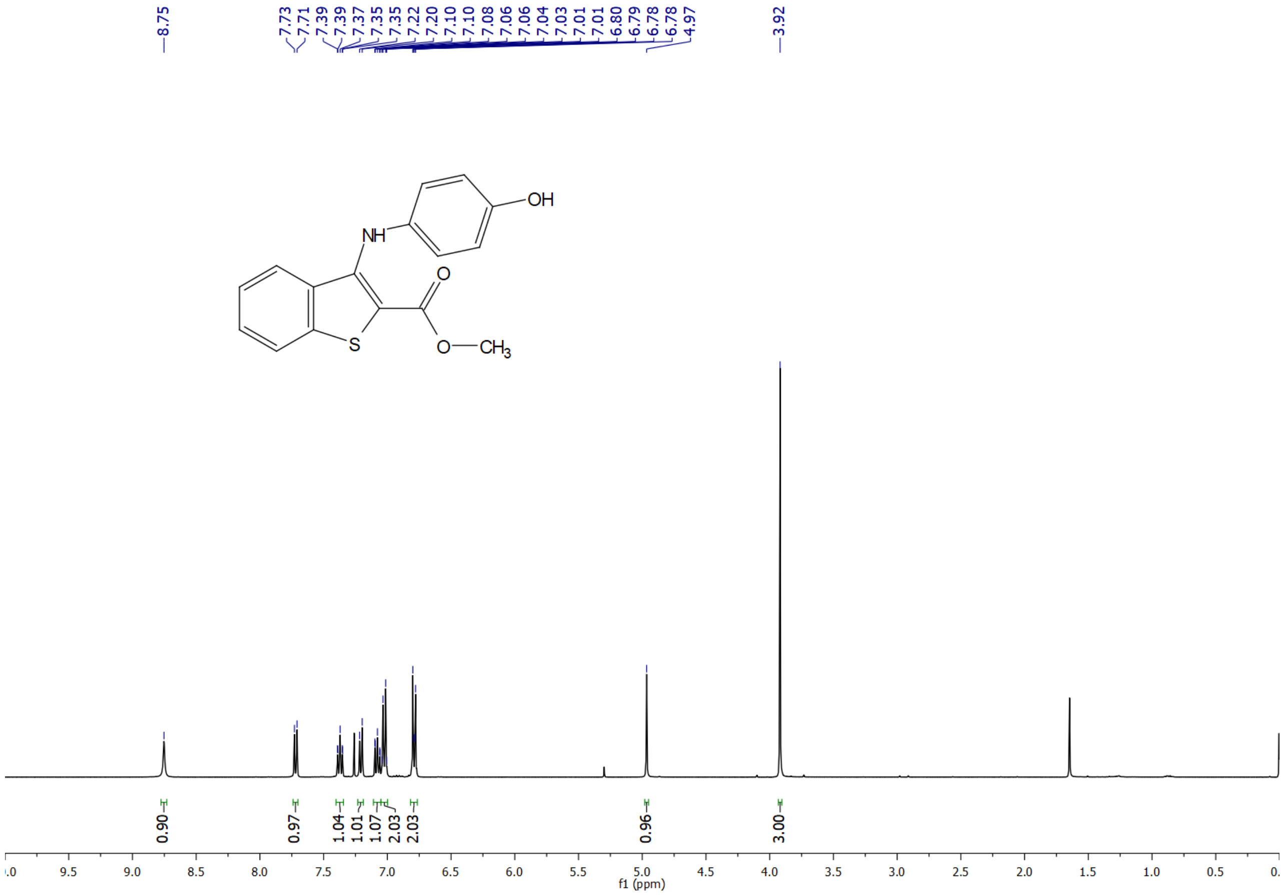

Figure S42. Methyl 3-[(4-hydroxyphenyl)amino]-1-benzothiophene-2-carboxylate (3m). ^{1}H NMR spectrum (400 MHz, $CDCl_3$).

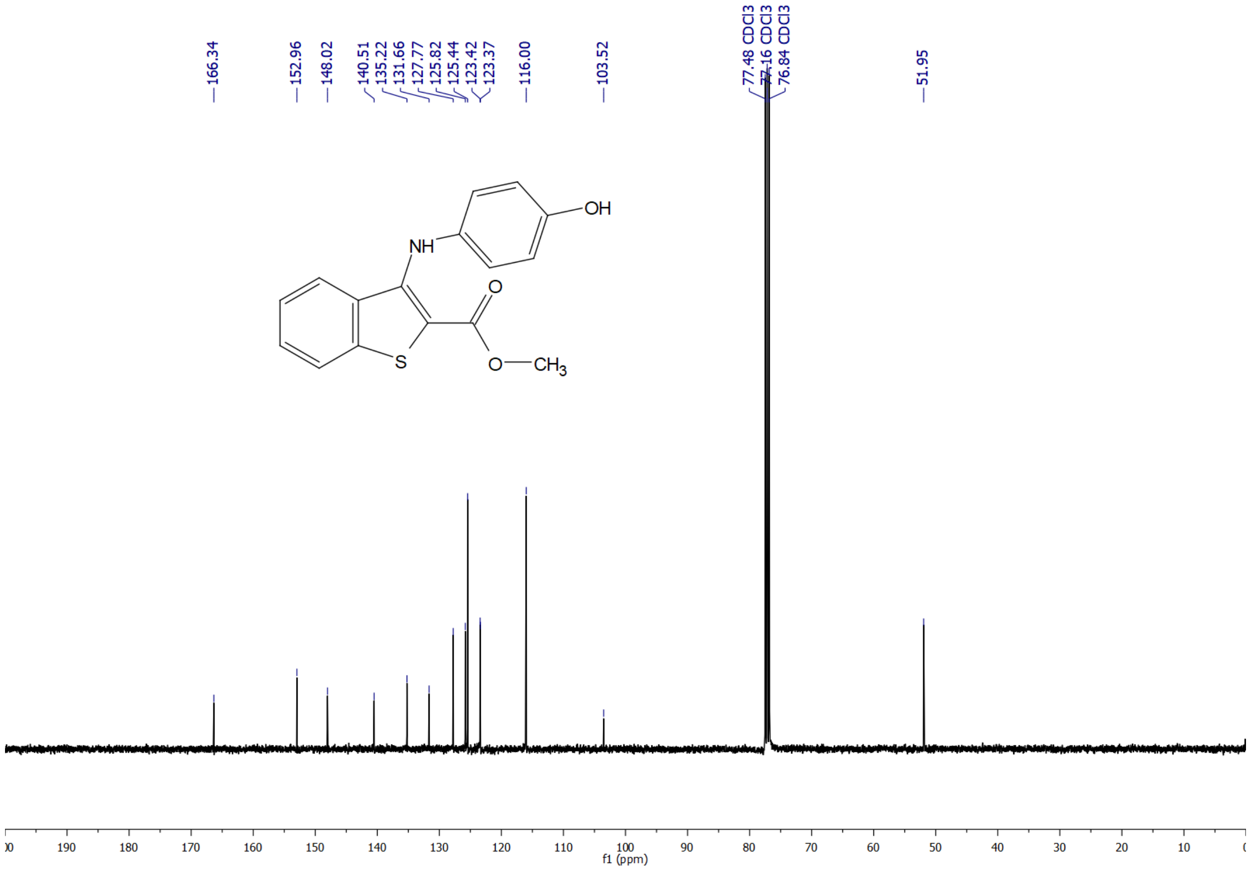

Figure S43. Methyl 3-[(4-hydroxyphenyl)amino]-1-benzothiophene-2-carboxylate (3m). ^{13}C NMR spectrum (100 MHz, $CDCl_3$).

Acquisition Parameter

Source Type	ESI	Ion Polarity	Positive	Set Nebulizer	0.6 Bar		
Focus	Active	Set Capillary	4500 V	Set Dry Heater	200 ℃		
Scan Begin	50 m/z	Set End Plate Offset	-500 V	Set Dry Gas	7.0 l/min		
Scan End	1850 m/z	Set Collision Cell RF	2500.0 Vpp	Set Divert Valve	Waste		

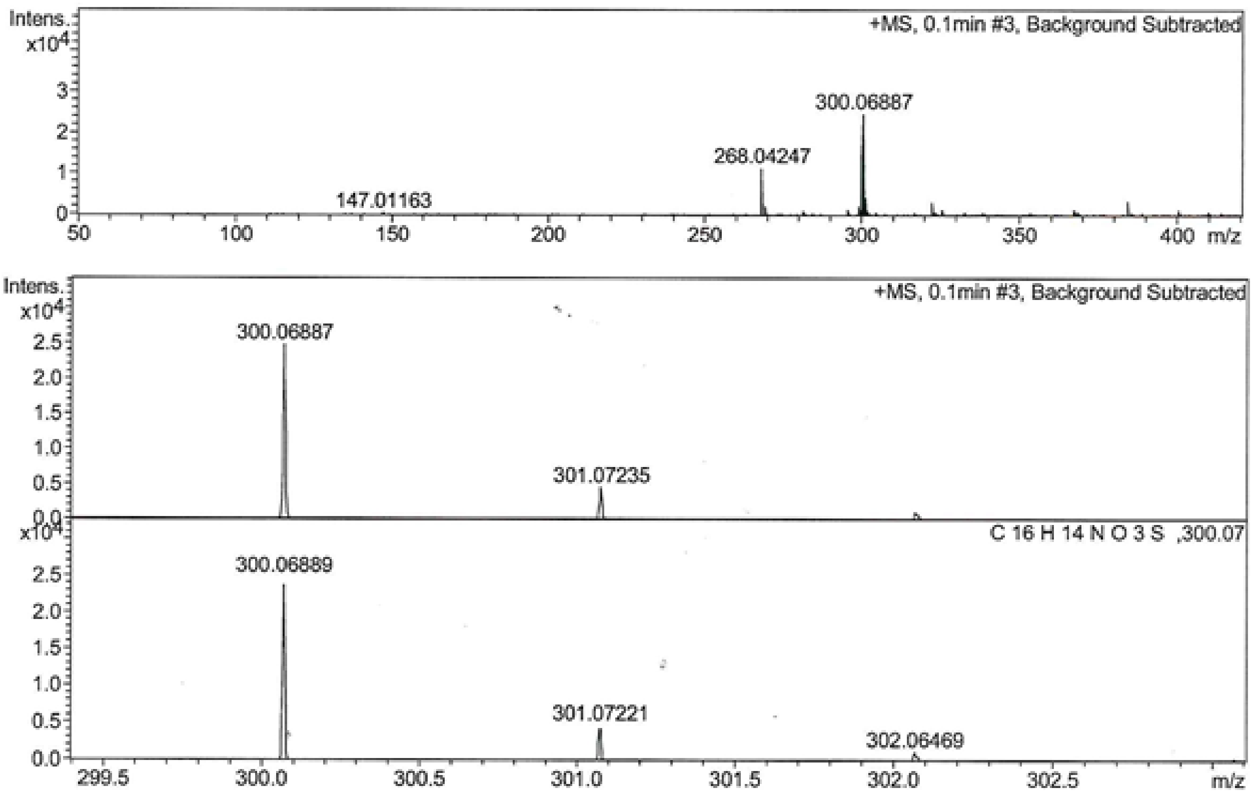

Meas. m/z	#	Formula	m/z	err [ppm]	mSigma	rdb	e⁻ Conf	N-Rule
268.04247	1	C 15 H 10 N O 2 S	268.04268	0.78	32.3	11.5	even	ok
300.06887	1	C 16 H 14 N O 3 S	300.06889	0.07	34.1	10.5	even	ok
322.05069	1	C 16 H 13 N Na O 3 S	322.05084	0.46	98.4	10.5	even	ok

Figure S44. Methyl 3-[(4-hydroxyphenyl)amino]-1-benzothiophene-2-carboxylate (3m). HRMS (ESI).

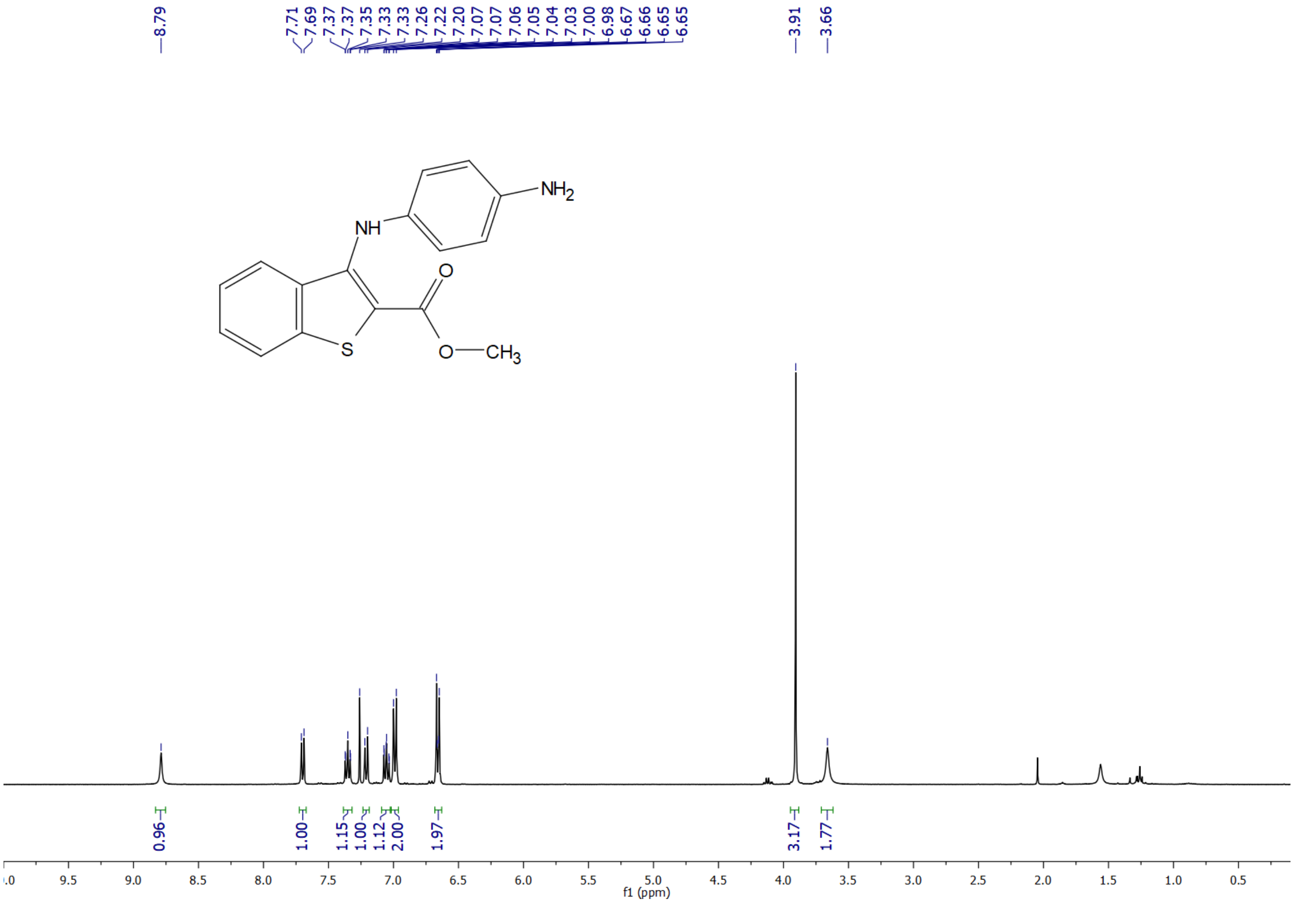

Figure S45. Methyl 3-[(4-aminophenyl)amino]-1-benzothiophene-2-carboxylate (3n). ^{1}H NMR spectrum (400 MHz, $CDCl_3$).

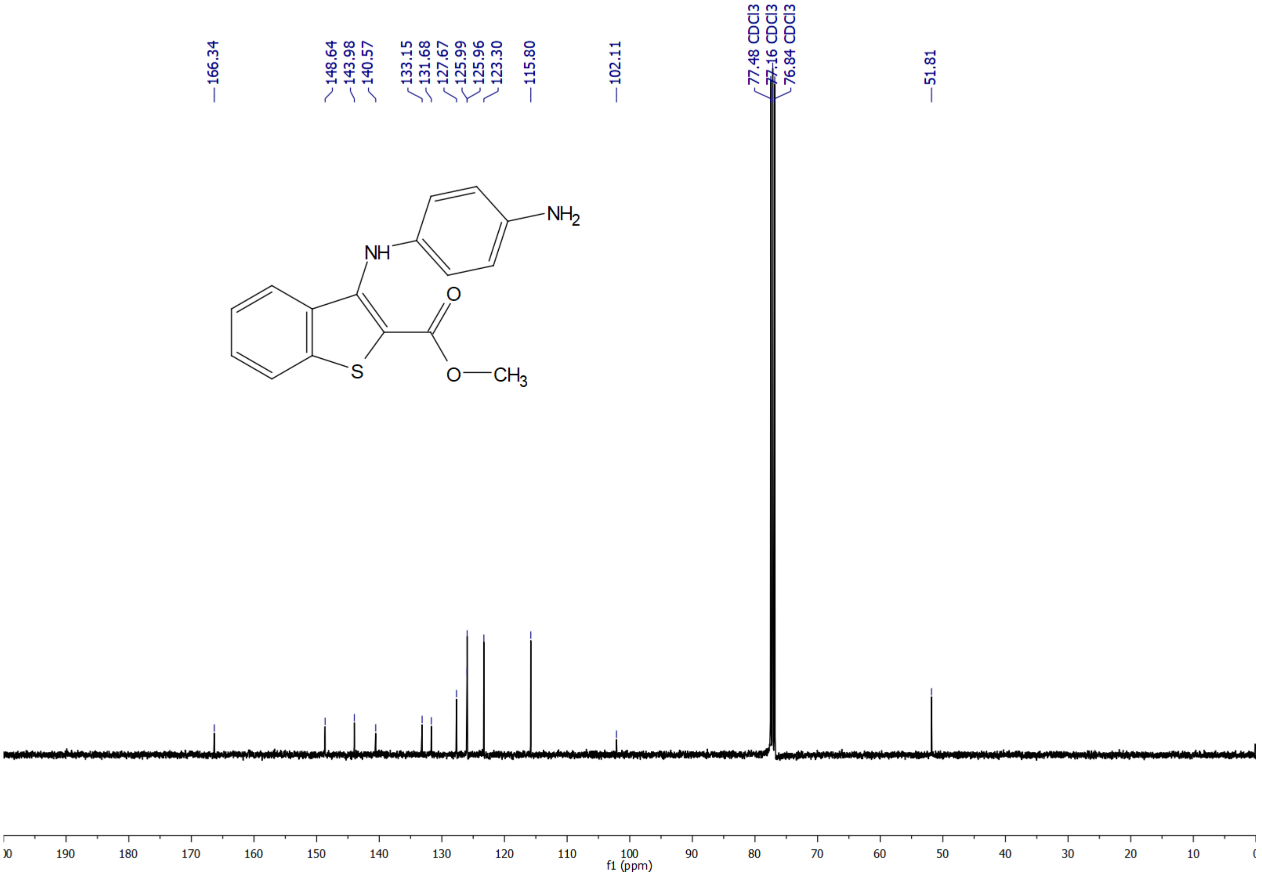

Figure S46. Methyl 3-[(4-aminophenyl)amino]-1-benzothiophene-2-carboxylate (3n). ^{13}C NMR spectrum (100 MHz, $CDCl_3$).

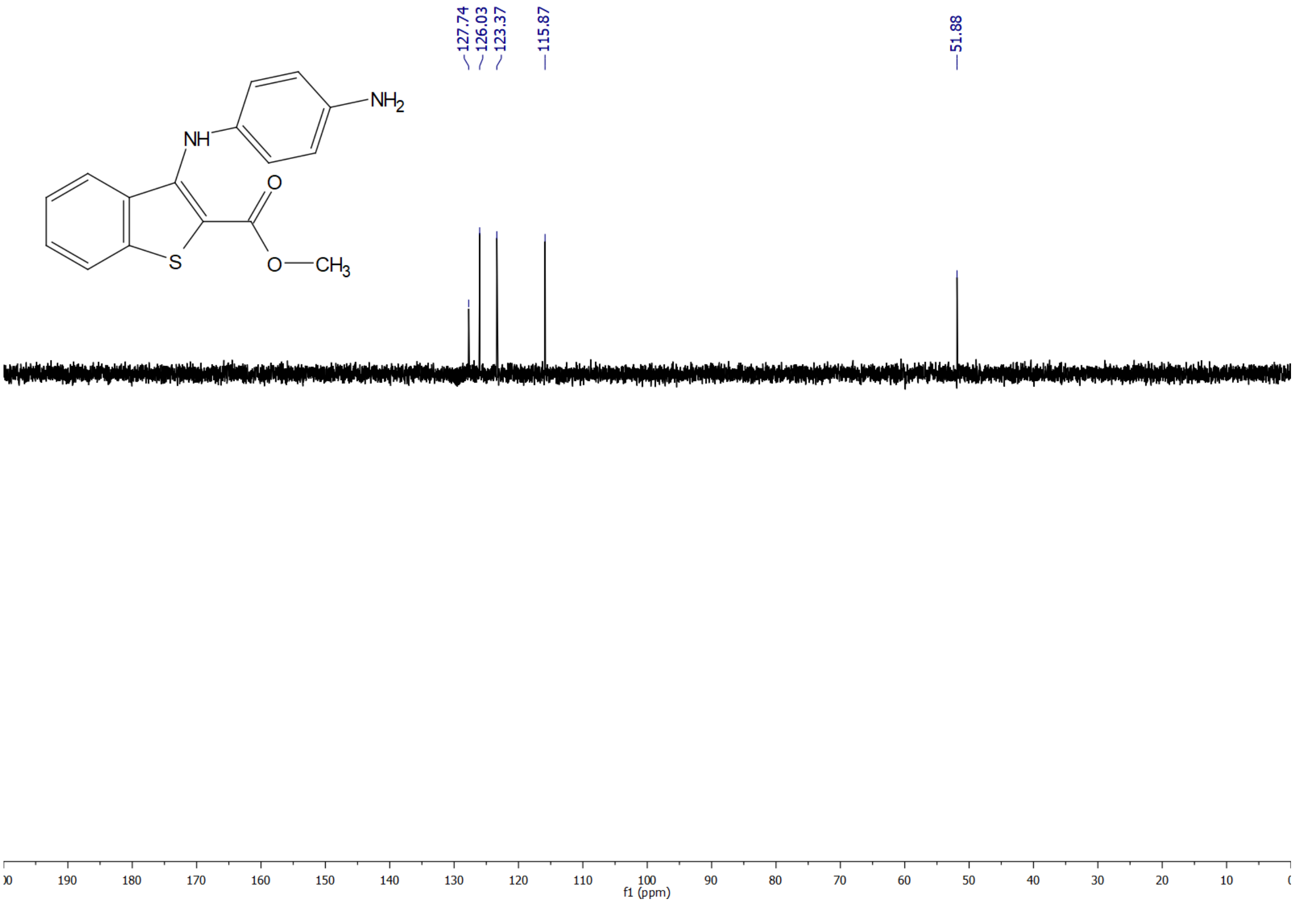

Figure S47. Methyl 3-[(4-aminophenyl)amino]-1-benzothiophene-2-carboxylate (3n). DEPT135 spectrum (100 MHz, $CDCl_3$).

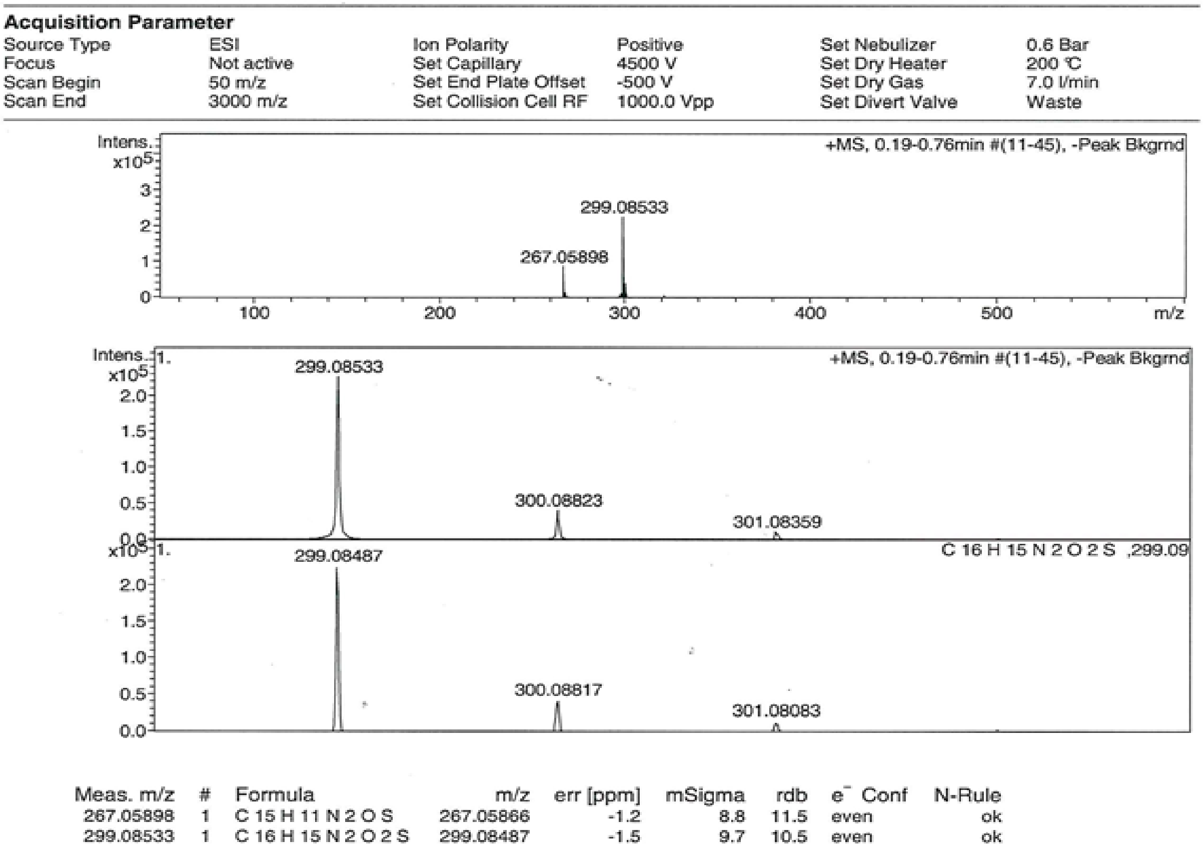

Figure S48. Methyl 3-[(4-aminophenyl)amino]-1-benzothiophene-2-carboxylate (3n). HRMS (ESI).

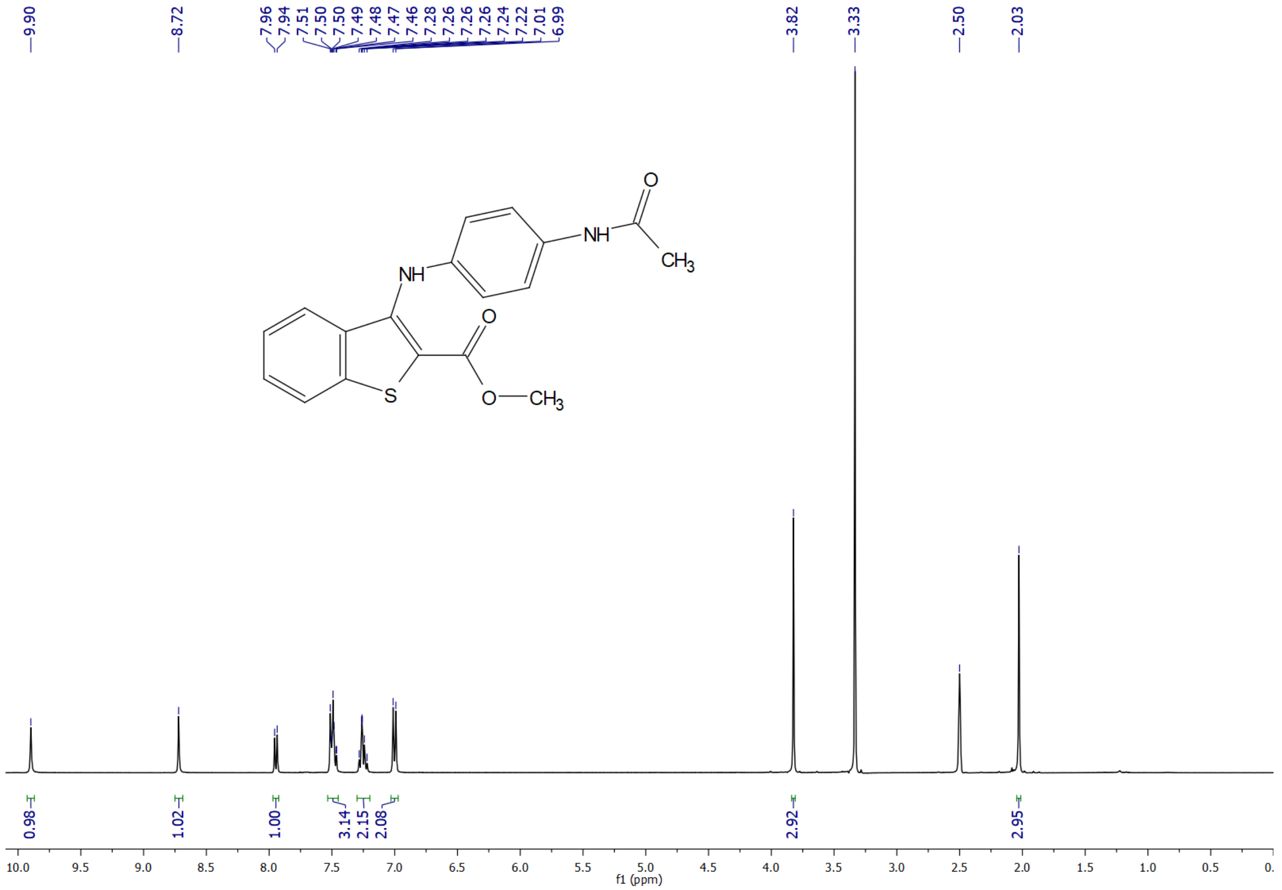

Figure S49. Methyl 3-{[4-(acetylamino)phenyl]amino}-1-benzothiophene-2-carboxylate (3o). ^{1}H NMR spectrum (400 MHz, DMSO-d_6).

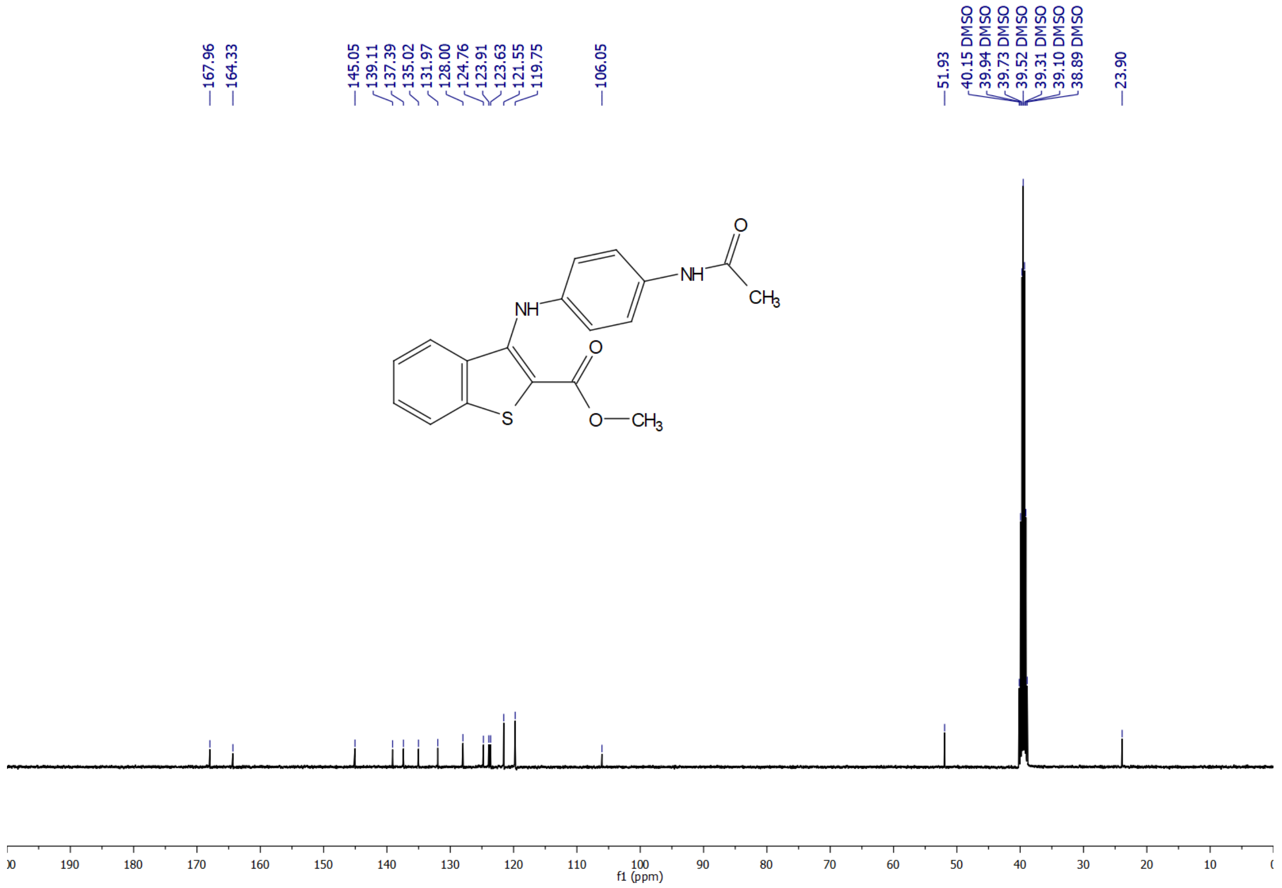

Figure S50. Methyl 3-{[4-(acetylamino)phenyl]amino}-1-benzothiophene-2-carboxylate (3o). ^{13}C NMR spectrum (100 MHz, DMSO-d_6).

Acquisition Parameter

Source Type	ESI	Ion Polarity	Positive	Set Nebulizer	0.6 Bar
Focus	Not active	Set Capillary	4500 V	Set Dry Heater	200 °C
Scan Begin	50 m/z	Set End Plate Offset	-500 V	Set Dry Gas	7.0 l/min
Scan End	3000 m/z	Set Collision Cell RF	1000.0 Vpp	Set Divert Valve	Waste

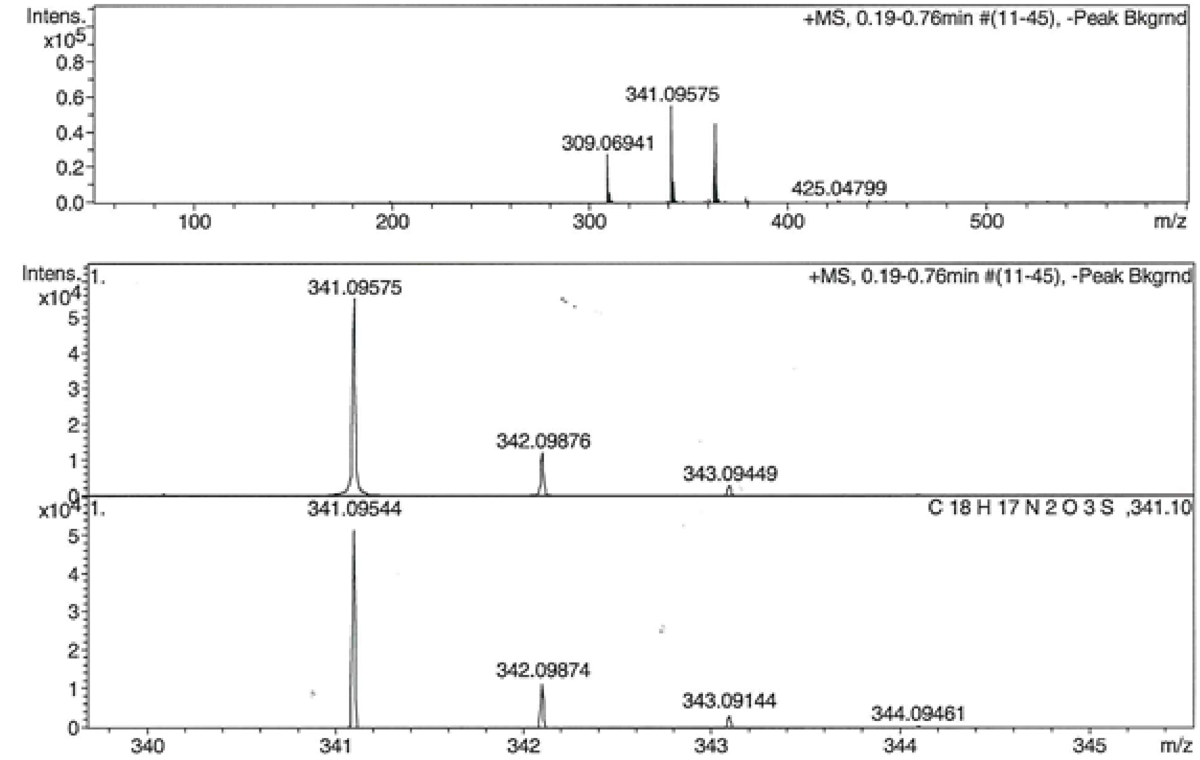

Meas. m/z	#	Formula	m/z	err [ppm]	mSigma	rdb	e⁻ Conf	N-Rule
309.06941	1	C 17 H 13 N 2 O 2 S	309.06922	-0.6	11.6	12.5	even	ok
341.09575	1	C 18 H 17 N 2 O 3 S	341.09544	-0.9	12.2	11.5	even	ok
363.07767	1	C 18 H 16 N 2 Na O 3 S	363.07738	-0.8	14.9	11.5	even	ok

Figure S51. Methyl 3-{[4-(acetylamino)phenyl]amino}-1-benzothiophene-2-carboxylate (3o). HRMS (ESI).

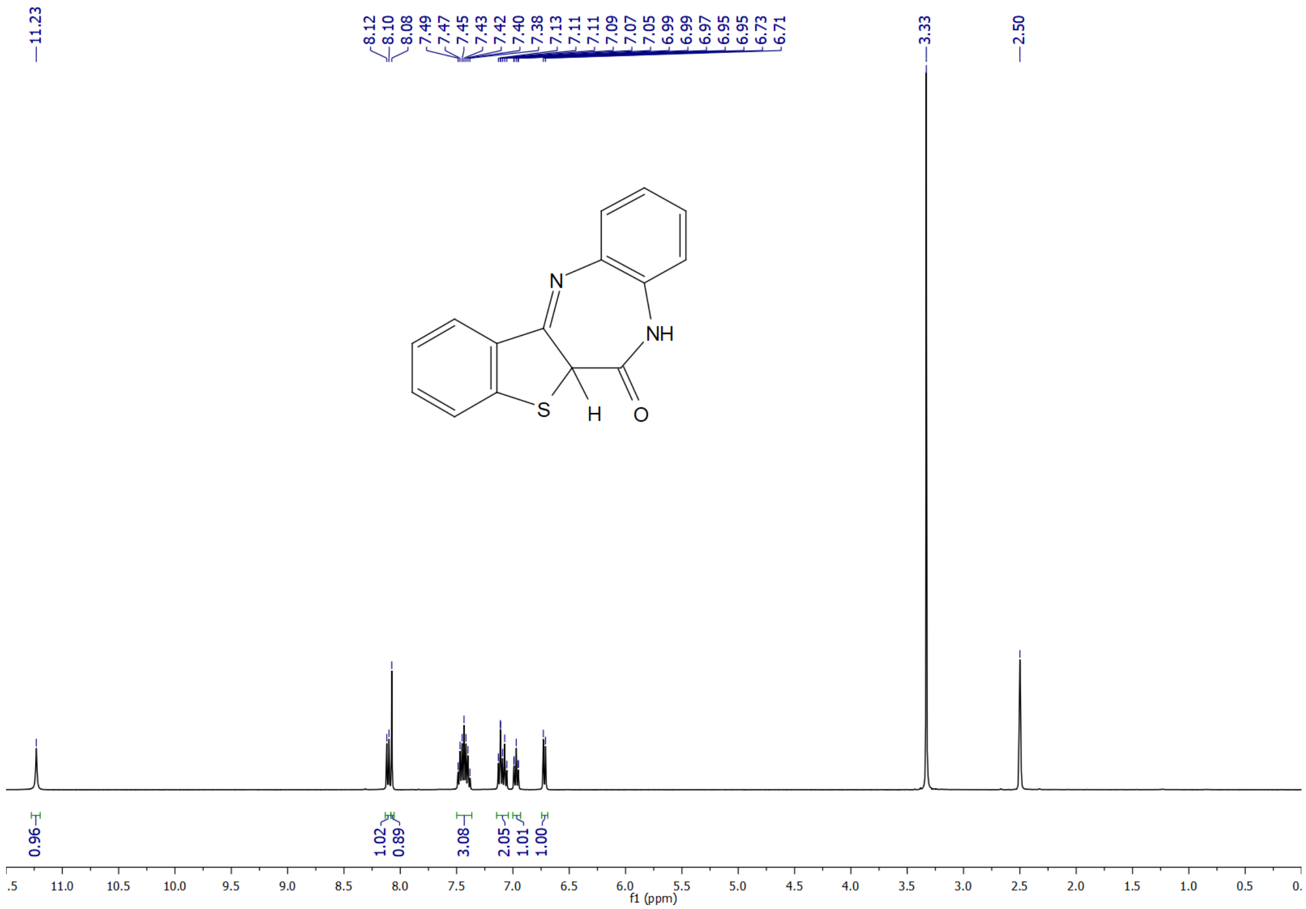

Figure S52. **5a,7-dihidro-6*H*-[1]benzothieno[2,3-*b*][1,5]benzodiazepin-6-one (4). ^{1}H NMR spectrum (400 MHz, DMSO-d_6).**

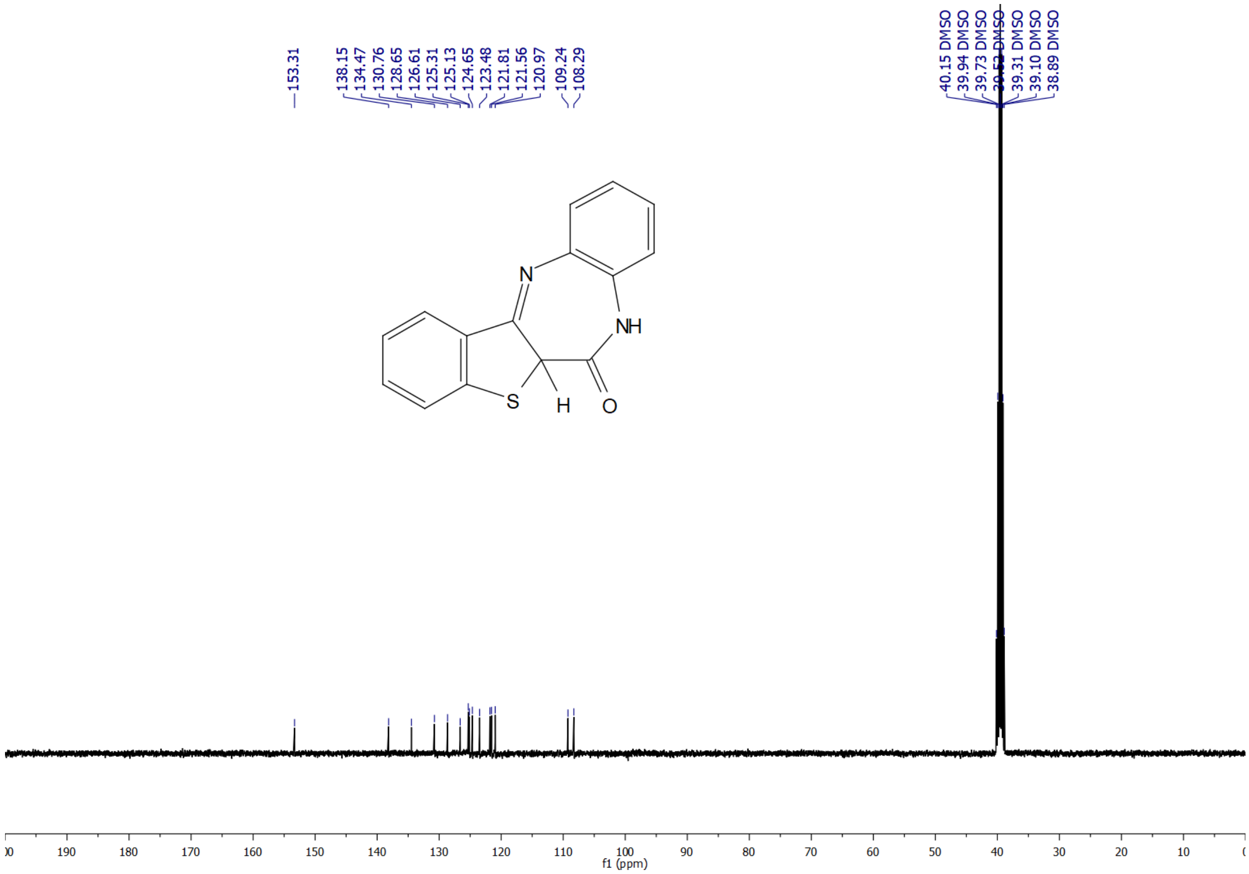

Figure S53. **5a,7-dihidro-6*H*-[1]benzothieno[2,3-*b*][1,5]benzodiazepin-6-one (4). ^{13}C NMR spectrum (100 MHz, DMSO-d_6).**

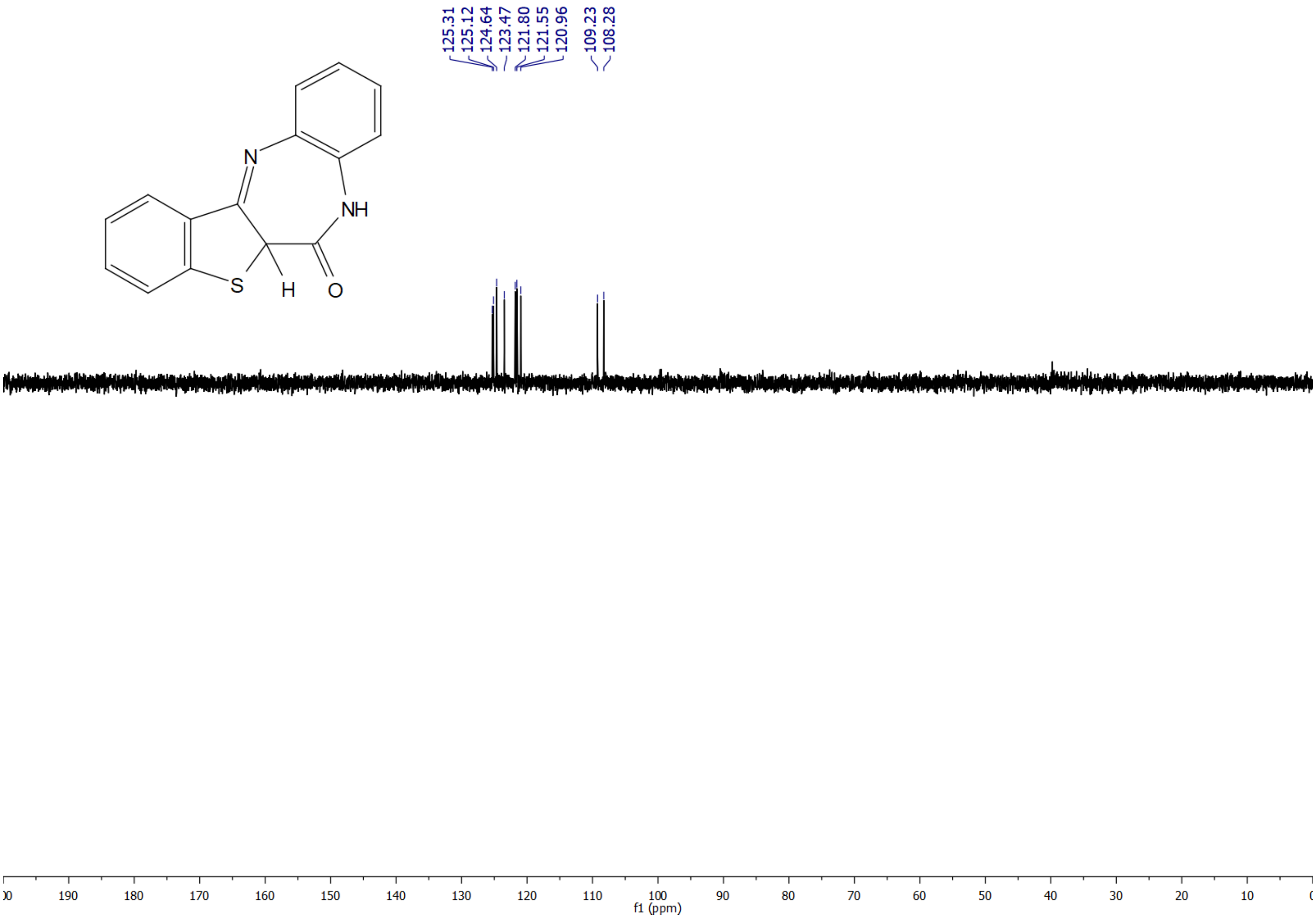

Figure S54. **5a,7-dihidro-6*H*-[1]benzothieno[2,3-*b*][1,5]benzodiazepin-6-one (4). DEPT135 spectrum (100 MHz, DMSO-d_6).**

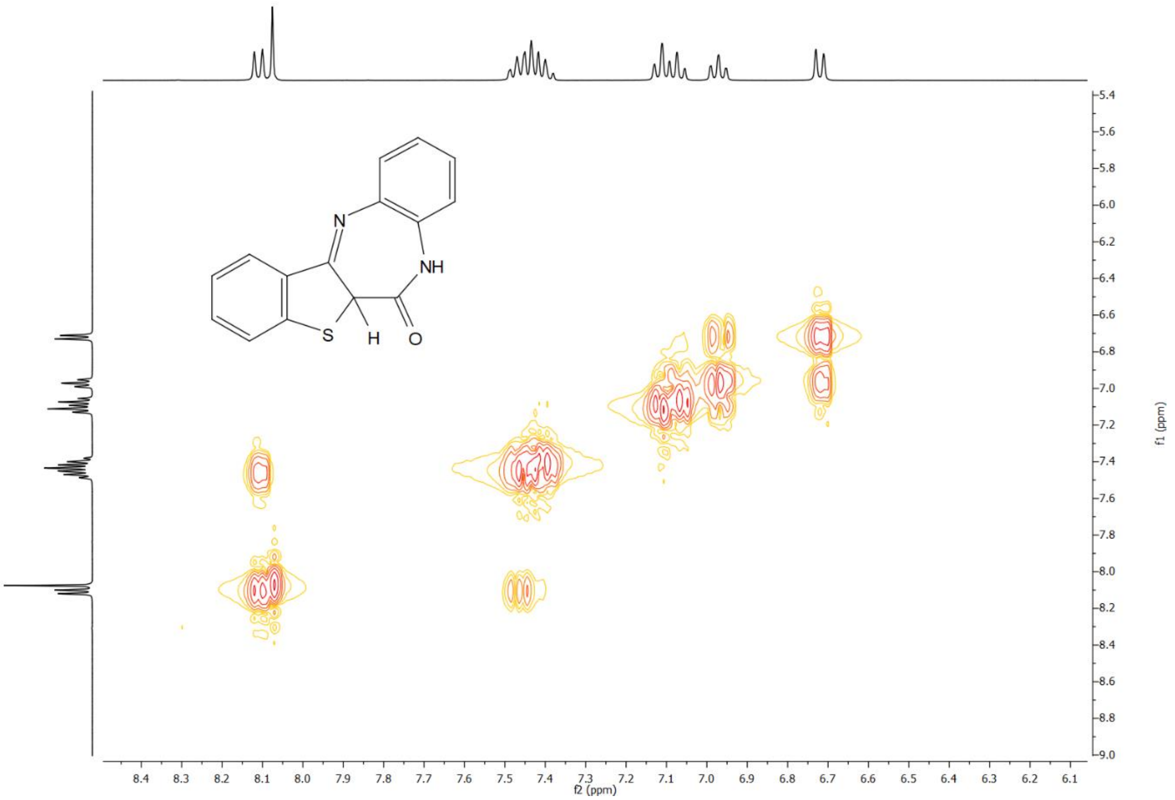

Figure S55. **5a,7-dihidro-6*H*-[1]benzothieno[2,3-*b*][1,5]benzodiazepin-6-one (4). COSY.**

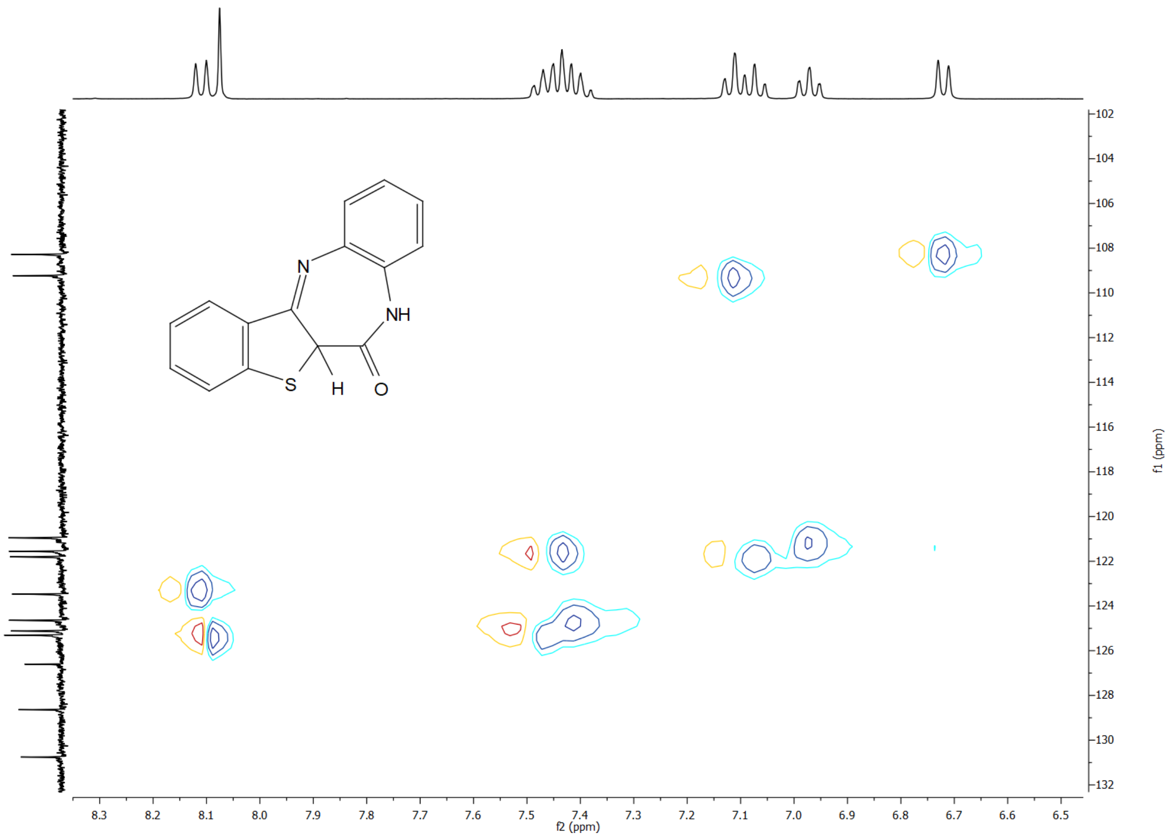

Figure S56. 5a,7-dihidro-6*H*-[1]benzothieno[2,3-*b*][1,5]benzodiazepin-6-one (4). HSQC.

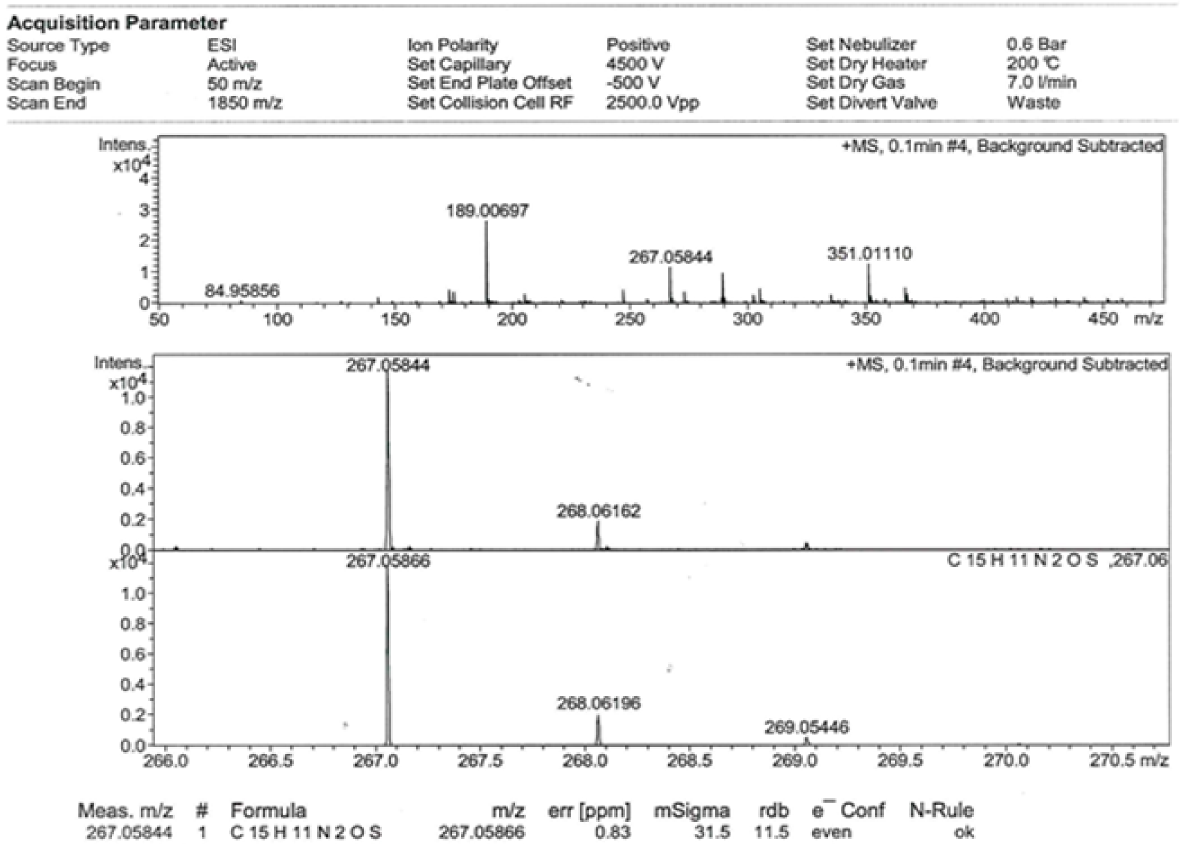

Figure S57. 5a,7-dihidro-6*H*-[1]benzothieno[2,3-*b*][1,5]benzodiazepin-6-one (4). HRMS (ESI).

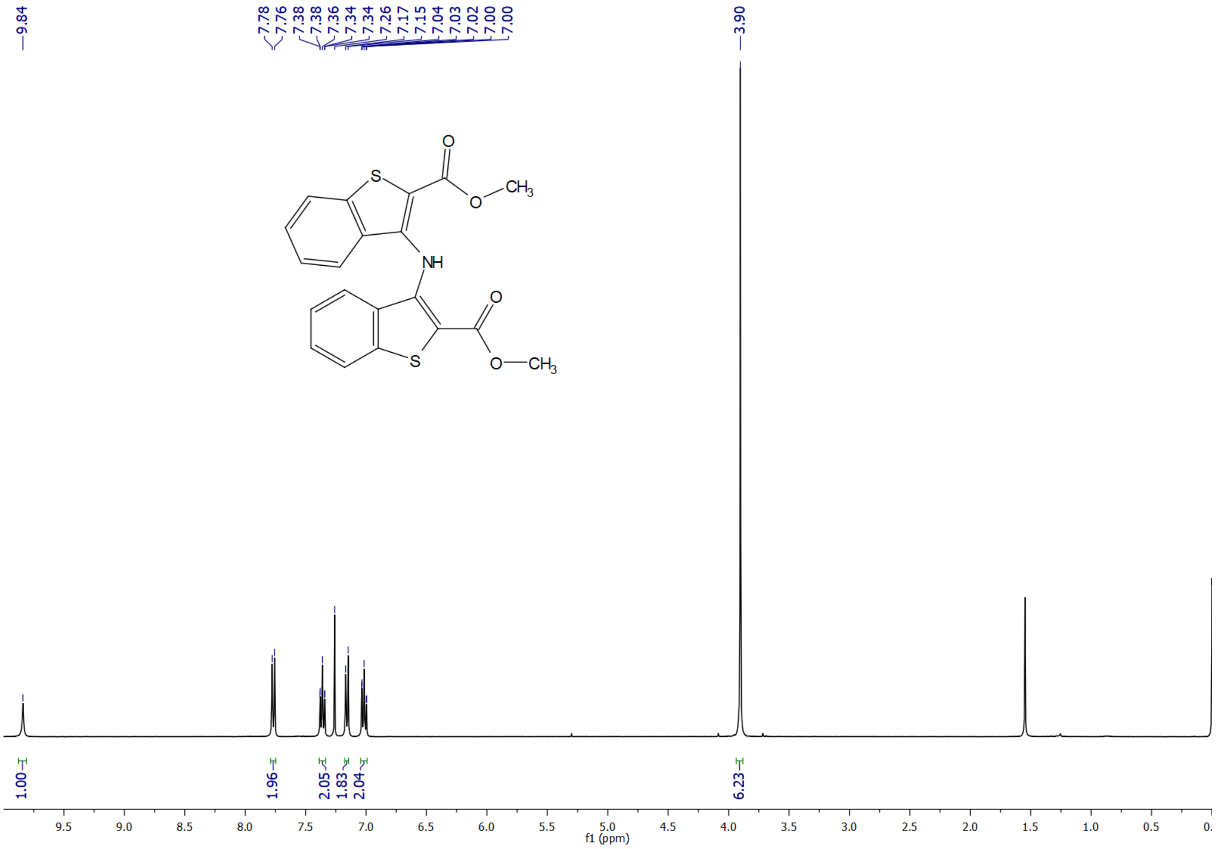

Figure S58. Dimethyl 3,3′-iminobis(benzo[*b*]thiophene-2-carboxylate) (5). ^{1}H NMR spectrum (400 MHz, $CDCl_3$).

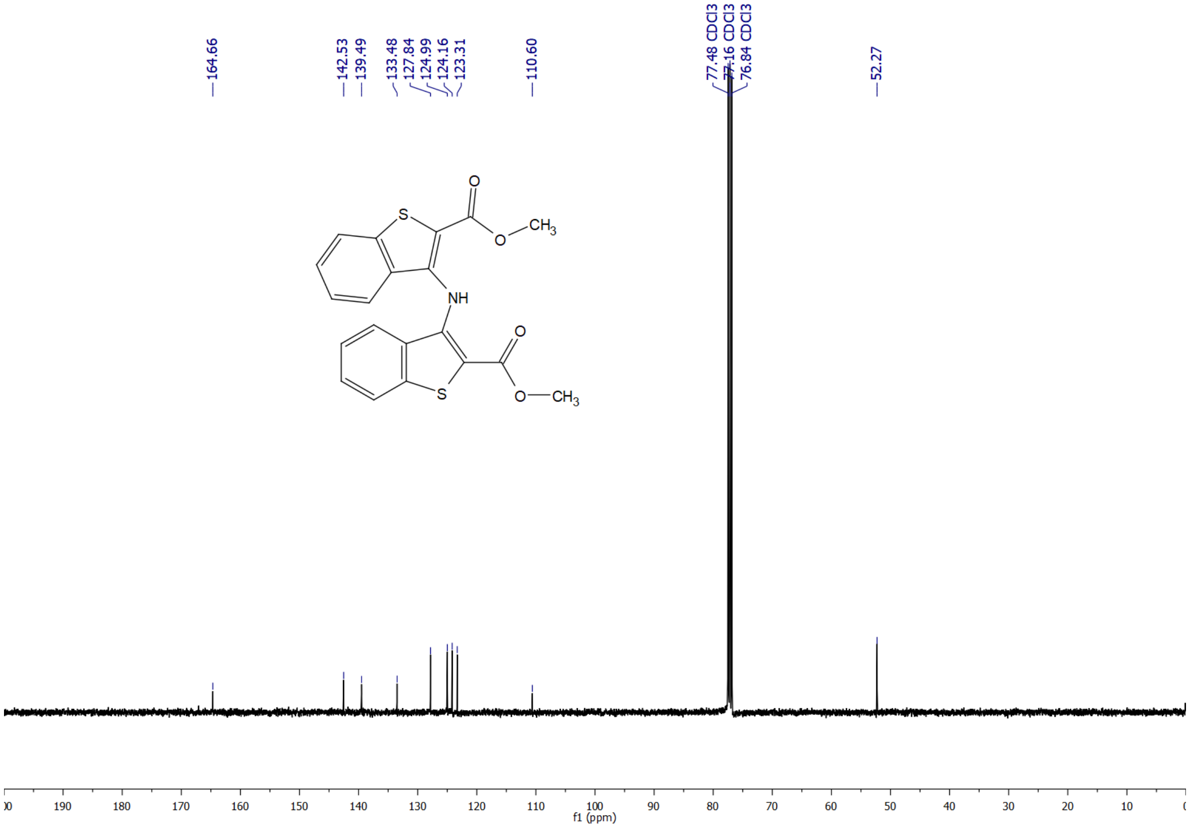

Figure S59. Dimethyl 3,3′-iminobis(benzo[*b*]thiophene-2-carboxylate) (5). ^{13}C NMR spectrum (100 MHz, $CDCl_3$).

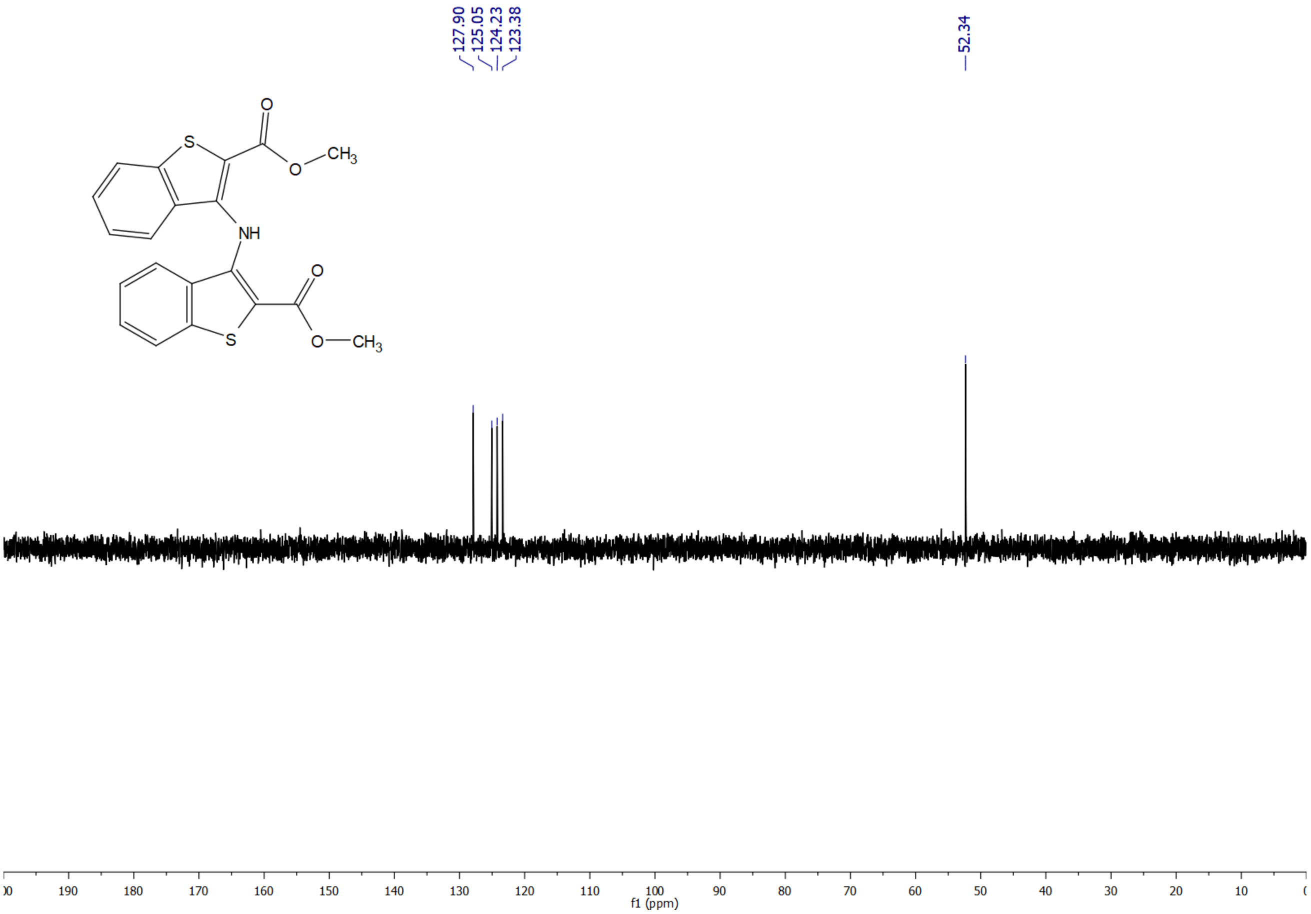

Figure S60. Dimethyl 3,3′-iminobis(benzo[*b*]thiophene-2-carboxylate) (5). DEPT135 spectrum (100 MHz, $CDCl_3$).

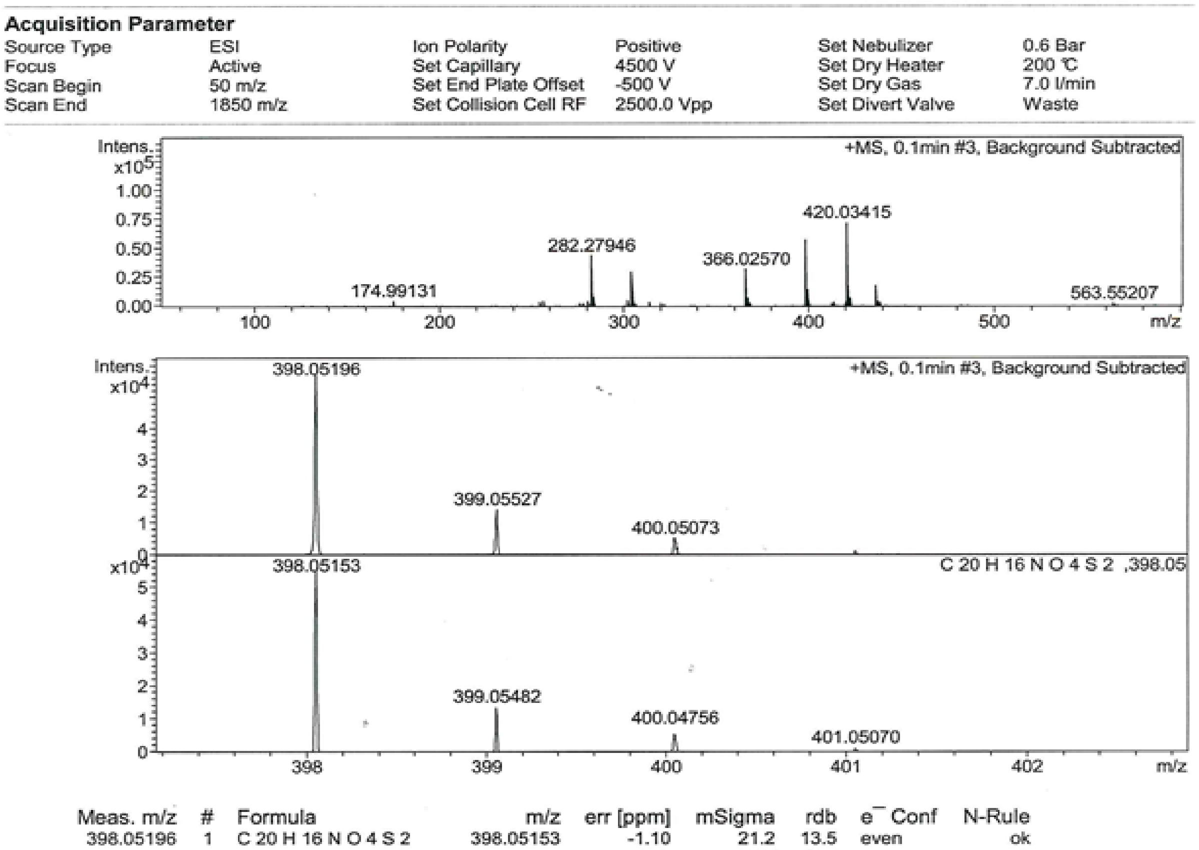

Figure S61. Dimethyl 3,3′-iminobis(benzo[*b*]thiophene-2-carboxylate) (5). HRMS (ESI).
